# Apelin-13-Mediated Upregulation of METTL3 Ameliorates Alzheimer’s Disease via Inhibiting Neuroinflammation Through m6A-Dependent Regulation of lncRNA BDNF-AS

**DOI:** 10.3390/biom15081188

**Published:** 2025-08-18

**Authors:** Li Han, Siwen Wei, Rong Wang, Yiran Liu, Yi Zhong, Juan Fu, Huaiqing Luo, Meihua Bao

**Affiliations:** 1 Hunan Provincial University Key Laboratory of the Fundamental and Clinical Research on Functional Nucleic Acid, the First Clinical College, Changsha Medical University, Changsha 410219, China; hanlidemeng@126.com (L.H.); td20230611@hunnu.edu.cn (Y.Z.); 2 Hunan Provincial Key Laboratory of the Traditional Chinese Medicine Agricultural Biogenomics, Changsha Medical University, Changsha 410219, China; 3 Department of Immunology, School of Medicine, Jishou University, Jishou 416000, China; weisiwen01@gmail.com; 4 Department of Physiology, School of Basic Medical Sciences, Hunan Normal University, Changsha 410013, China; wangrongrongzyk@163.com (R.W.); 202470193650@hunnu.edu.cn (Y.L.); 5 College of Biological and Pharmaceutical Engineering, Shandong University of Aeronautics, Binzhou 256603, China; sparrowfj@126.com; 6 Key Laboratory of Model Animals and Stem Cell Biology in Hunan Province, School of Medicine, Hunan Normal University, Changsha 410013, China

**Keywords:** Alzheimer’s disease, Apelin-13, METTL3, lncRNA BDNF-AS, m6A methylation, neuroinflammation

## Abstract

Apelin-13, a neuropeptide, has been recognized for its neuroprotective properties. Our previous study found apelin-13 improves cognitive function in Alzheimer’s disease (AD) rats by inhibiting neuroinflammation through upregulation of BDNF/TrkB signaling pathway. However, the precise mechanism by which apelin-13 modulates BDNF remains unclear. Thus, this study aimed to unravel the specific regulatory mechanism by which apelin-13 regulates BDNF. Bilaterally intracerebroventricular injection with Aβ25–35 was used to establish an in vivo model of AD. For the generation of METTL3 KO rats, the Crispr/Cas9 method was applied. PC12 cells were treated with Aβ25–35 to establish an in vitro model of AD. The cognitive function of the rats was evaluated with the Morris water maze and the novel object recognition test. Hippocampal damage and neuron loss were detected through H&E and immunofluorescent staining. METTL3, BDNF, TrkB, and p-TrkB were examined by Western blotting. Inflammation-related cytokines, IBA1, GFAP, IL-1β, and TNF-α were detected by Western blotting, immunofluorescent staining, ELISA, and qRT-PCR. m6A modification level was evaluated through MeRIP. A flow cytometer was applied to evaluate cell apoptosis. Cell proliferation was examined using MTT. m6A methylation inhibitor DAA reverses the improvement effect of apelin-13 on cognitive function, hippocampal nerve damage, neuron loss, and neuroinflammation in Aβ25–35-treated rats. Further results showed that apelin-13 upregulated METTL3, BDNF-AS m6A methylation, inhibited BDNF-AS expression, and subsequently upregulated BDNF/TrkB signaling pathway and reduced neuroinflammation in in vivo and in vitro AD models in a dose-dependent manner. Knockdown of METTL3 abolished apelin-13’s improvement effect in AD rats. Apelin-13-mediated upregulation of METTL3 enhances neuroinflammation inhibition and BDNF/TrkB signaling pathway via m6A-dependent downregulation of lncRNA BDNF-AS, thus ameliorating AD. Our study offers novel insights into the pathogenesis of AD and identifies potential drug targets for its treatment.

## 1. Introduction

Alzheimer’s disease (AD) is a progressive neurodegenerative disorder characterized by the accumulation of amyloid-beta plaques and neurofibrillary tangles (NFTs), which lead to synaptic dysfunction and brain cell loss [1,2]. These pathological features subsequently cause significant cognitive decline, memory impairment, and behavioral changes, bringing enormous pain and pressure to patients and their families [3]. With the increasingly serious aging of the population in China, the number of AD patients has become one of the highest in the world [4]. Currently available pharmacological treatments for AD offer limited benefits, mainly focusing on symptom management. These medications, such as cholinesterase inhibitors and NMDA receptor antagonists, may provide modest relief but do not halt the progression of the disease [5,6]. Therefore, in-depth exploration of the pathogenesis of AD and the search for new, effective efficacious preventive and therapeutic agents have become the focus of current research.

Brain-derived neurotrophic factor (BDNF), widely distributed in the brain, is a key neurotrophic factor [7]. Previous studies have consistently shown that BDNF expression is decreased in AD patients [8,9]. Our analysis using the AlzData database showed that BDNF expression was significantly reduced in the hippocampus, temporal cortex, and prefrontal cortex of AD patients (Figure S1). Studies have shown that activation of BDNF can reduce memory impairment in AD mice [10,11]. Our previous study showed that the upregulation of BDNF/TrkB signaling improved cognitive impairment of AD rats through inhibiting neuroinflammation [12]. These studies suggest that BDNF plays an important role in AD. However, the mechanism by which BDNF is downregulated in AD remains unclear.

Brain-derived neurotrophic factor antisense RNA (BDNF-AS), a long non-coding RNA (lncRNA) transcribed from the opposite strand of the BDNF gene, can inhibit the transcription of BDNF by recruiting EZH2 (H3K27 methylase) in cells [13,14]. Studies have shown that BDNF-AS is upregulated in AD patients [15]. Therefore, the decreased expression of BDNF in AD patients might be due to the increased expression of BDNF-AS.

One study showed that BDNF-AS is related to N6-adenylate (m6A) methylation in Bladder Cancer [16]. Analysis using the SRAMP database identified a high-confidence m6A methylation site on BDNF-AS (Figure S2). Therefore, we speculated that the increased expression of BDNF-AS in AD may be associated with abnormal m6A methylation. m6A is the most common internal modification in RNA. Among the enzymes involved, methyltransferase-like 3 (METTL3), a key m6A methyltransferase, is downregulated in AD [17]. Its low expression is associated with a high risk of AD [18]. Knockdown of METTL3 or 3-deazaadenosine (DAA) treatment, an inhibitor of RNA methylation, led to the disturbance of synaptic plasticity in AD [19]. Therefore, we hypothesized that METTL3 might affect the expression of BDNF-AS by regulating the m6A methylation, thereby participating in the progression of AD.

Apelin, a bioactive neuropeptide, is an endogenous ligand for angiotensin II type 1 receptor (AT1)-related protein (APJ) [19]. Studies have found that APJ regulates the expression of METTL3 [20]. The results of AlzData database analysis showed that APJ was significantly upregulated in the entorhinal cortex, hippocampus, temporal cortex, and prefrontal cortex in early-stage AD patients (Figure S3). Therefore, we hypothesized that APJ may regulate METTL3 expression in AD. This regulation might influence the expression of BDNF-AS and BDNF. Apelin-13 (AP), an active form of apelin, primarily binds to APJ and is widely distributed in brain regions related to cognitive function, including the hippocampus and cerebral cortex [21]. Studies have shown that the level of AP in the serum of AD patients is significantly reduced [22]. Intravenous injection of AP can improve memory dysfunction induced by chronic stress in rats [23]. In addition, our previous research showed that AP can improve cognitive function in AD rats by inhibiting neuroinflammation through upregulating BDNF/TrkB signaling pathway [12]. Therefore, it is speculated that AP may regulate METTL3-mediated BDNF-AS m6A methylation by acting on its receptor APJ, thereby ultimately affecting BDNF expression and AD progression.

Therefore, this study focused on the METTL3/BDNF-AS/BDNF signaling pathway and provided insights into the mechanism of AP in AD. The results of this study may provide novel ideas and potential drug targets for the prevention and treatment of AD.

## 2. Materials and Methods

### 2.1. Reagents

AP (4029110, Bachem, Bubendorf, Switzerland); Aβ25–35 (A4559, Sigma-Aldrich, St. Louis, MO, USA); thiazolyl blue (MTT) (M2128, Sigma-Aldrich, St. Louis, MO, USA); PC12 cell line (Shanghai Cell Bank, Chinese Academy of Sciences, Shanghai, China); microglia bv2 (CL-0493, Pricella, Wuhan, China); PrimeScriptTM RT reagent Kit (RR037A, Takara, Tokyo, Japan); ELISA kit (IL-1β, ERC007QT and TNF-α, ERC102aQT.96, Neobioscience Technology, Shenzhen, China); ANNEXIN V-FITC/PI Apoptosis Detection Kit (CA1020, Solarbio, Beijing, China); protein secondary antibody (A21020 and A21010, Abbkine, Wuhan, China), β3-tubulin (ab18207, Abcam, Cambridge, MA, USA), NeuN (ab177487, Abcam), anti-β-actin (ab8227, Abcam); BDNF (ab108319, Abcam), METTL3 (ab195352, Abcam), IBA 1 (ab178846 m, Abcam), GFAP (ab7260, Abcam), TrkB (ab187041, Abcam), p-TrkB (ab229908, Abcam), DAA (6736-58-9, MCE, Shanghai, China).

### 2.2. Animals and Experimental Groups

Male Sprague-Dawley (SD) rats, weighing 180–200 g, were procured from Hunan Slake Jinda Laboratory Animal Co., Ltd. (Changsha, China), Production License No.: SCXK (Hunan) 2016–0002. All rats were maintained in a specific pathogen-free (SPF) environment with controlled temperature (20 ± 2 °C), humidity (50–70%), and a 12 h light–dark cycle. Complied with the 3R principle, the experimental procedures were approved by the Animal Ethics Committee of Changsha Medical University.

Aβ25-35 Aging treatment: According to the previous research for the preparation of the Aβ25-35 modeling drug [24], Aβ25-35 powder was configured into a masterbatch with a concentration of 1 mmol/mL using sterile double-distilled water and placed into a 37 °C, 5% CO2 incubator for aging for 7 d.

METTL3 gene knockout: tThe Crisper/Cas9 method was used to construct METTL3 knockout rats (KO). The sgRNA1 and sgRNA2 were designed for the upstream and downstream of METTL3, respectively. These two sgRNAs were constructed into a vector and packaged into an adenovirus. Five-week-old male Cas9 transgenic rats were from Cyagen Biosciences Inc. (Suzhou, China) These rats have carried the Cas9 gene and expressed Cas9 protein. The sgRNA adenovirus(titer: 1.2 × 10^12^ GC/mL), packaged in the first step, was injected into the Cas9 tool rats via bilateral hippocampus (2 μL/site). Western blotting was employed to assess the knockout efficiency.

Grouping and treatment: Wild-type rats were randomly assigned to the Control WT, sham WT, AD WT, AD+AP-L, M, H (0.5, 1, 2 μg/each) groups [25], AD+AP+DAA (methylation inhibitor) group, and 8 per group. The sample size was calculated based on the pre-experiment (effect size d = 0.8, α = 0.05, β = 0.2) by G*Power 3.1. METTL3 knockout rats were randomly grouped into sham KO, AD KO, and AD+AP KO (2 μg/each) groups, and 8 per group. After one week of acclimation, AD rat model was induced in all groups, excluding the Control and sham group. Rats in each group underwent intraperitoneal injection of 60 mg·kg^−1^ sodium pentobarbital for anesthesia. Subsequently, the anesthetized rats were secured in a brain stereotaxic apparatus. Bilateral targeting of the hippocampus was performed at 3.5 mm behind the fontanel, 2 mm beside the midline, and 2.9 mm below the dura mater. After a 3-day recovery, except for the sham group, rats received a slow infusion of 5 μL (10 μg) of Aβ25-35 into both hippocampal tissues over 5 min using a micro syringe pump. The sham group received an equivalent volume of sterile saline. AP was injected into the hippocampus once a day for 4 weeks. For AD+AP+DAA group, DAA was administered bilaterally in the hippocampus 30 min before AP injection. After behavioral assessments, rats were euthanized with sodium pentobarbital. From each group, three brain tissues were randomly chosen and submerged in 4% paraformaldehyde for section staining, while the hippocampus tissues from the remaining samples were removed and preserved at −80 °C.

### 2.3. Behavioral Experiments

#### 2.3.1. The Novel Object Recognition Test (NOR)

The apparatus was composed of a black Plexiglas box measuring 60 × 60 × 60 cm and positioned on the floor. The light intensity was adjusted to 80 ± 10 lux. For the test, objects made of glass and plastic, varying in shape, color, and texture, were utilized. Specifically, two such objects were placed in the rear corners of the box. To prevent any potential biases toward a particular corner or object, the locations and objects were counterbalanced across each group. The behavioral procedure encompassed two phases: a training session and a subsequent testing session. During the training session, the rat had a 5 min period to explore two identical objects, and the total exploration time for both was recorded. Exploration was defined as the act of sniffing the object within a 1-cm radius or touching it. Between trials, both the box and objects were meticulously cleaned using a 20% alcoholic solution to eliminate olfactory cues, followed by wiping with dry paper. After a 24 h interval, the testing session commenced, where the rat was exposed to two objects for 5 min—one familiar from the previous session and one novel. A discrimination index (DI) was calculated to differentiate between the novel and familiar object, using the formula: [(time spent exploring the novel object − time spent exploring the familiar object)/(time spent exploring the novel object + time spent exploring the familiar object)] × 100%.

#### 2.3.2. Morris Water Maze (MWM)

As mentioned earlier, the Morris Water Maze (MWM) test was utilized to investigate spatial learning and memory [26]. The experiment consisted of a 5-day phase for spatial navigation, followed by a 6th day dedicated to spatial exploration. Throughout the 120 s trials, we recorded the swimming trajectories, number of crossing platforms, and time spent in different quadrants.

### 2.4. m6A RNA Methylation Quantification

The EpiQuik m6A RNA Methylation Quantitation Kit (Colorimetric) from Epigentek(P-9005, Farmingdale, NY, USA) was used to determine the overall RNA m6A level. Initially, total RNAs were extracted from hippocampal tissues or PC12 cells and quantified using NanoDrop 2000 (ND-2000-BAS, Thermo, Wilmington, DE, USA). Subsequently, 200 ng of total RNA and the binding solution were mixed in each well and incubated at 37 °C for 1.5 h. Afterward, a series of reagents—including capture antibody, detection antibody, enhancer solution, developer solution, and stop solution—were sequentially added to each well and allowed to react for the prescribed durations. Ultimately, the relative m6A level in each group was assessed by measuring the absorbance at 450 nm using a microplate reader from Molecular Devices ( i3x, Mountain View, CA, USA).

### 2.5. Methylated RIP-qPCR (MeRIP-qPCR)

m6A modification on *lncRNA BDNF-AS* was examined using the Magna MeRIP m6A Kit (17-10499, Millipore, Darmstadt, Germany) following the manufacturer’s instructions. In brief, a total of 300 μg of RNA was fragmented to approximately 100 nucleotides. Prior to use, 10 μg of m6A antibody (ab151230, Abcam, Cambridge, UK) was coated onto protein A/G magnetic beads (88803, Thermo Fisher Scientific, Vienna, Austria). Following this, the RNA fragments were thoroughly mixed with the m6A antibody-coated beads under rotating conditions for 2 h. Subsequently, the methylated RNAs that were immunoprecipitated were eluted and subjected to PCR analysis. The primers for RT-qPCR are shown in Table 1.

### 2.6. H&E and Immunofluorescence Staining

Rats’ hippocampal tissues were removed gently and fixed in 4% formaldehyde overnight. The next day, the tissues underwent dehydration and were embedded in paraffin. These paraffin-embedded tissues were then sectioned into 5-μm slices. For H&E staining, slices were deparaffinized and stained with hematoxylin and eosin (C0105S, Beyotime, Shanghai, China). For immunofluorescence staining, slices were routinely dehydrated and subjected to antigen repair with citrate. After being washed with PBST, slices were blocked using 5% serum for 1 h, then incubated overnight at 4 °C with the following primary antibodies: β3-tubulin (1:500), NeuN (1:500), IBA1 (1:500), and GFAP (1:500). After being washed in PBS, the slices were subsequently added with DAPI and the corresponding secondary antibodies. Slices were then incubated in the dark at room temperature for 2 h, followed by PBS wash. Images were captured using a Pannoramic Digital Slide Scanner (3DHISTECH, Budapest, Hungary). For cell counting analyses, three rats per group were used, and the three best-labeled sections from each rat were selected for cell enumeration. The slide codes were randomized by independent personnel, with decoding conducted following the analysis. Immunoreactivity levels were measured using ImageJ software (1.54p, NIH, Bethesda, MD, USA).

### 2.7. ELISA

Rat brain tissue samples were collected, and the ELISA kit was used to measure the levels of IL-1βand TNF-αaccording to the manufacturer’s instructions.

### 2.8. Cell Sources and Grouping

PC-12 cell culture was conducted following previously described protocols [27]. Briefly, PC-12 cells were cultured in Dulbecco’s Modified Eagle Medium (DMEM) with 10% FBS and 1% penicillin-streptomycin. All cells were maintained in an incubator at 5% CO_2_, 37 °C, and 95% relative humidity. PC-12 cells were plated at 2 × 10^4^ cells/cm^2^ for 24 h. Cells in the logarithmic growth phase were selected for the experiment and divided into five groups: the control group, the Aβ25-35 group, and the Aβ25-35+AP-L (1 nmol/L), M (10 nmol/L), and H (100 nmol/L) groups. Except for the control group, we added Aβ25−35 (20 µmol/L) and/or AP to the medium and incubated it for 24 h. The cells were then collected for the next experiment.

### 2.9. MTT Assay for Cell Proliferation

Following incubation, 10 µL of MTT solution (concentration of 5 mg/mL) was added to each well and allowed to incubate for 4 h. Afterward, the medium was removed by aspiration, and 100 µL of DMSO (CAS number 67-68-5, sourced from Sinopharm, Beijing, China) was added. The mixture was then shaken for 15 min. The absorbance value was subsequently measured at a wavelength of 490 nm using a microplate reader.

### 2.10. Flow Cytometry to Detect Apoptosis

An equal quantity of cells was digested using EDTA-free trypsin and collected. Subsequently, the cells were rinsed twice with pre-chilled PBS, which was then removed by centrifugation at 1000 rpm. After adding 500 μL of binding buffer, the cells were resuspended. Next, 10 μL of Annexin V-FITC was introduced and mixed on an ice bath, followed by a 15 min incubation period in the dark. Apoptosis was then assayed.

### 2.11. qRT-PCR

RNA was extracted from hippocampal tissues and cells using TRIzol reagent (15596026, Invitrogen Co., Carlsbad, CA, USA). The concentration of mRNA was determined using a NanoDrop Spectrophotometer (ND-2000C-BL, Thermo Fisher Scientific, Wilmington, USA). Subsequently, 1 µg of mRNA was reverse-transcribed into cDNA utilizing the PrimeScriptTM RT reagent Kit (RR037A, Takara, Tokyo, Japan), following the manufacturer’s guidelines. The qRT-PCR reactions were carried out with SYBR Green reagents (RR820A, Takara, Tokyo, Japan) in a CFX Opus Real-Time PCR System (12013744, Bio-Rad, Hercules, USA). The expression levels of the target gene were normalized to the expression of the housekeeping gene beta-actin (*β-actin*). Table 1 displays the primer design for the PCR. The primers used for the experiment are listed in Table 1.

### 2.12. Western Blotting

Western blotting analysis was performed as described previously [9]. Proteins were extracted from both PC-12 cells and hippocampal tissues and separated using SDS-PAGE gel. The proteins were then transferred onto a PVDF membrane (ISEQ00010, Millipore, Carrigtwohill, Ireland) through the wet transfer method, followed by blocking with 5% skimmed milk for 2 h. Subsequent steps included incubation with primary and secondary antibodies, and color development was achieved with the addition of an ECL luminescent agent. Quantification of BDNF (1:1000), METTL3 (1:1000), IBA1 (1:1000), GFAP (1:1000), TrkB (1:1000), and p-TrkB (1:1000) expression levels was performed using ImageJ densitometry software, utilizing β-actin as an internal reference.

### 2.13. Statistical Analysis

The data processing program utilized was SPSS 22.0 (Version: 22.0.0.2, IBM, Armonk, NY, USA). A normal distribution was validated through the Shapiro–Wilk test. Statistical comparisons between two groups were conducted using the *t*-test. For the KO experiments, a two-way ANOVA followed by Tukey post hoc was used for statistical analysis. Other analyses involving multiple groups utilized a one-way ANOVA. For data that did not follow a normal distribution, the Kruskal–Wallis test with multiple comparisons was utilized. Data are expressed as mean ± standard error (SEM). Significance was determined at a *p*-value less than 0.05.

## 3. Results

### 3.1. AP Improved Cognitive Impairment in AD Rats by Upregulating m6A Methylation

**Figure 1:**
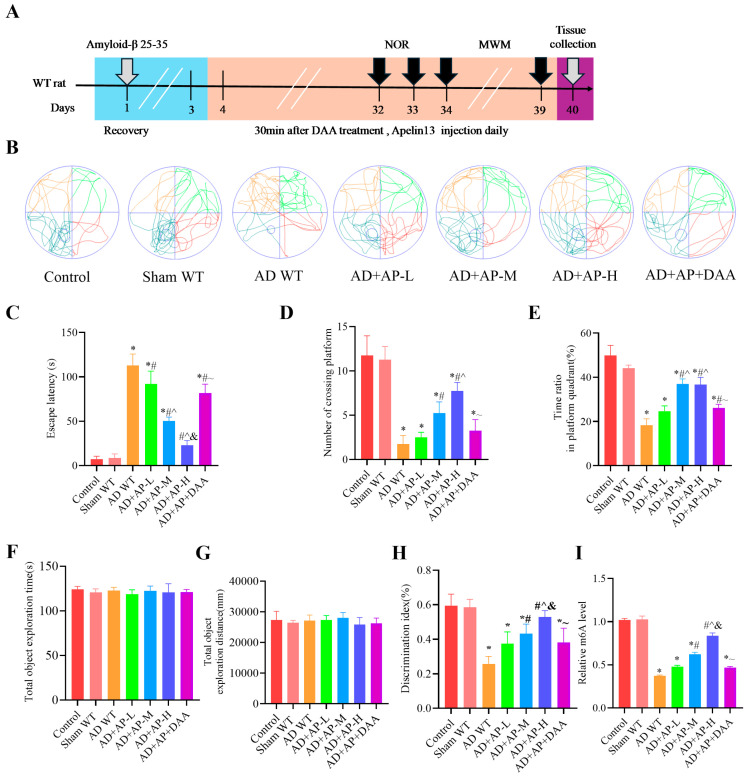
AP ameliorated cognitive impairment of AD rats by upregulating m6A methylation level. (**A**) A schematic illustration depicting the experimental designs and procedure. In the Morris water maze test: (**B**) Swimming trajectories of rats in Spatial probe test. (**C**) Escape latency. (**D**) Number of crossing platform. (**E**) Time ratio in platform quadrant. In the Novel object recognition test: (**F**) Total object exploration time. (**G**) Total object exploration distance. (**H**) Discrimination index. (**I**) Relative m6A level. Data is expressed as mean ± SEM, *n* = 8 per group, * *p <* 0.05 vs. sham WT, # *p <* 0.05 vs. AD WT, ^ *p <* 0.05 vs. AD+AP-L, & *p <* 0.05 vs. AD+AP-M, ~ *p <* 0.05 vs. AD+AP-H.

The timeline of experiments is shown in Figure 1A. NOR and MWM were used to assess cognitive function in rats. In the MWM test, AD WT rats showed longer escape latency, fewer platform crossings, and a lower time ratio in the platform quadrant (*p <* 0.05, Figure 1B–E), suggesting a compromised spatial learning ability. AP treatment dose-dependently improved the cognitive skills of AD WT rats (*p <* 0.05, Figure 1B–E). However, DAA pretreatment reversed this improvement effect (*p <* 0.05, Figure 1B–E).

In the NOR test, statistical analysis revealed no notable difference in the exploration of identical objects among the seven groups during the training phase (*p >* 0.05, Figure 1F,G). This indicates that drug injection (Aβ25-35, AP, and DAA) has no influence on object preference. Post hoc Tukey’s analysis showed that there was no significant difference between the Control group and sham WT group (*p >* 0.05, Figure 1H), suggesting that the surgery had no effect on the cognitive function of rats. A significant decrease in DI was observed in AD WT rats, when compared with the sham group. While AP treatment dose-dependently improved the cognitive capacity of AD WT rats (*p <* 0.05, Figure 1H). The AD+AP-H group had no significant difference from the Control group. DAA pretreatment reversed this improvement effect (*p <* 0.05, Figure 1H). Further studies found that AP indeed upregulated m6A methylation levels in a dose-dependent manner. These results revealed that AP, especially AP-H, significantly alleviated the cognitive impairment of AD WT rats by regulating m6A methylation.

### 3.2. AP Alleviated Hippocampal Damage and Neuroinflammation in AD Rats by Upregulating m6A Methylation

**Figure 2:**
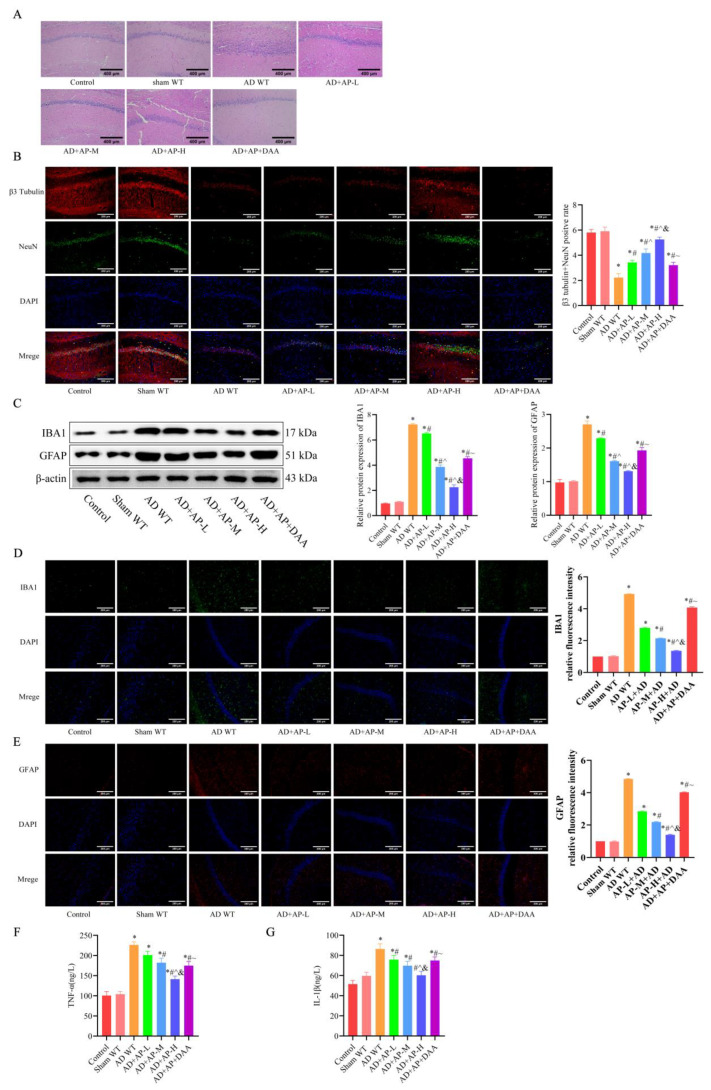
AP alleviated hippocampal pathology of AD rats by upregulating m6A methylation level. (**A**) Representative images of H&E staining in the CA1 region of hippocampus. (**B**) Representative immunofluorescence images of β3 tubulin (red) and NeuN (green) (neural markers) and DAPI staining(blue), and the graph shows the percentage of β3 tubulin and NeuN positive cells from 10 randomly chosen fields. *n* = 3, Scale bar: 200 μm. (**C**) Protein levels of IBA 1 and GFAP detected by Western blotting (*n* = 3). (**D**) Representative immunofluorescence images of IBA 1 and its relative intensity. *n* = 3, Scale bar: 200 μm. (**E**) Representative immunofluorescence images of GFAP and its relative intensity. *n* = 3, Scale bar: 200 μm. (**F**) Inflammatory factor TNF-α expression by ELISA, *n* = 6. (**G**) Inflammatory factor IL-1β expression by ELISA, *n* = 6. Original images can be found in Supplementary File S1. Data is expressed as mean ± SEM, * *p <* 0.05 vs. sham WT, # *p <* 0.05 vs. AD WT, ^ *p <* 0.05 vs. AD+AP-L, & *p <* 0.05 vs. AD+AP-M, ~ *p <* 0.05 vs. AD+AP-H.

HE staining and immunofluorescence were employed to evaluate nerve damage in the hippocampus of rats. The HE staining results showed that hippocampal neurons of AD WT rats exhibited obvious pathological damage, including sparse and disorganized arrangement, cell shrinkage, deepened staining, and nuclear consolidation. AP dose-dependently alleviated neuronal impairment of AD WT rats, while DAA administration reversed the improvement effect of AP (Figure 2A). β3-tubulin and NeuN, neuronal markers, were significantly decreased in the AD WT group, indicating neuron loss, but AP treatment dose-dependently alleviated neuron loss. DAA reversed the improvement effect of AP (*p* < 0.05, Figure 2B).

IBA1 and GFAP are important markers of microglia and astrocytes activation, respectively, and their elevated expression indicates enhanced inflammation. Western blotting results showed IBA1 and GFAP protein expression in the hippocampus increased significantly in the AD WT group, which was reversed by AP dose-dependently. However, DAA administration negated the AP effect (Figure 2C). Immunofluorescence results further confirmed it (Figure 2D,E). Elevated TNF-α (Figure 2F) and IL-1β (Figure 2G) levels in the AD WT group were also reversed by AP treatment in a dose-dependent manner, while DAA treatment abolished this effect (Figure 2F). The above results suggested that AP could alleviate the hippocampal pathology of AD rats by upregulating m6A methylation.

### 3.3. AP Effects on lncRNA BDNF-AS and Its Methylation and BDNF/TrkB Signaling Pathway in AD Rats

**Figure 3:**
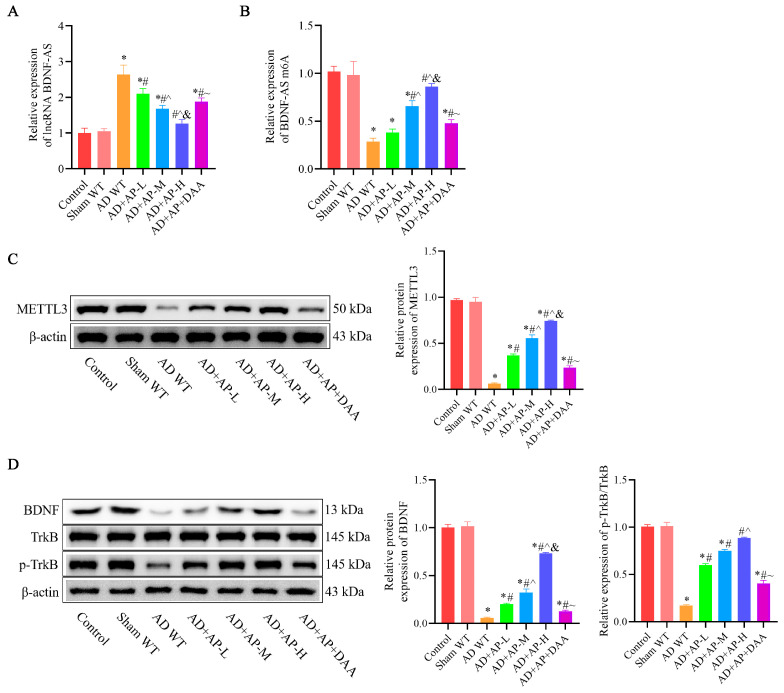
AP effects on lncRNA BDNF-AS and its methylation and BDNF/TrkB signaling pathway in AD rats. (**A**) RNA levels of *lncRNA BDNF-AS* detected by qRT-PCR. (**B**) RNA levels of *BDNF-AS m6A* detected by MeRIP-qPCR. (**C**) Protein level of METTL3 detected by Western blotting. (**D**) Protein levels of BDNF, TrkB, and p-TrkB detected by Western blotting. Original images can be found in Supplementary File S1. Data is expressed as mean ± SEM, *n* = 3. * *p <* 0.05 vs. sham WT, # *p <* 0.05 vs. AD WT, ^ *p <* 0.05 vs. AD+AP-L, & *p <* 0.05 vs. AD+AP-M, ~ *p <* 0.05 vs. AD+AP-H.

In the AD WT group, *lncRNA BDNF-AS* was significantly upregulated, while *BDNF-AS m6A* expression was markedly downregulated compared to the sham group. AP treatment reversed these changes in a dose-dependent manner, while DAA addition negated these effects (Figure 3A,B). METTL3 protein expression was substantially reduced in the AD WT group. AP treatment effectively restored it in a dose-dependent manner, but DAA administration reversed this effect (Figure 3C). Additionally, BDNF and p-TrkB protein levels were significantly decreased in the AD WT group. AP treatment restored their levels in a dose-dependent manner, but DAA administration negated these effects (Figure 3D). These observations suggested that AP dose-dependently inhibits lncRNA BDNF-AS expression, which in turn enhances BDNF/TrkB signaling pathway by increasing methylase protein METTL3 expression.

### 3.4. Effect of AP on Cognitive Function in METTL3 Knockout Rats

**Figure 4:**
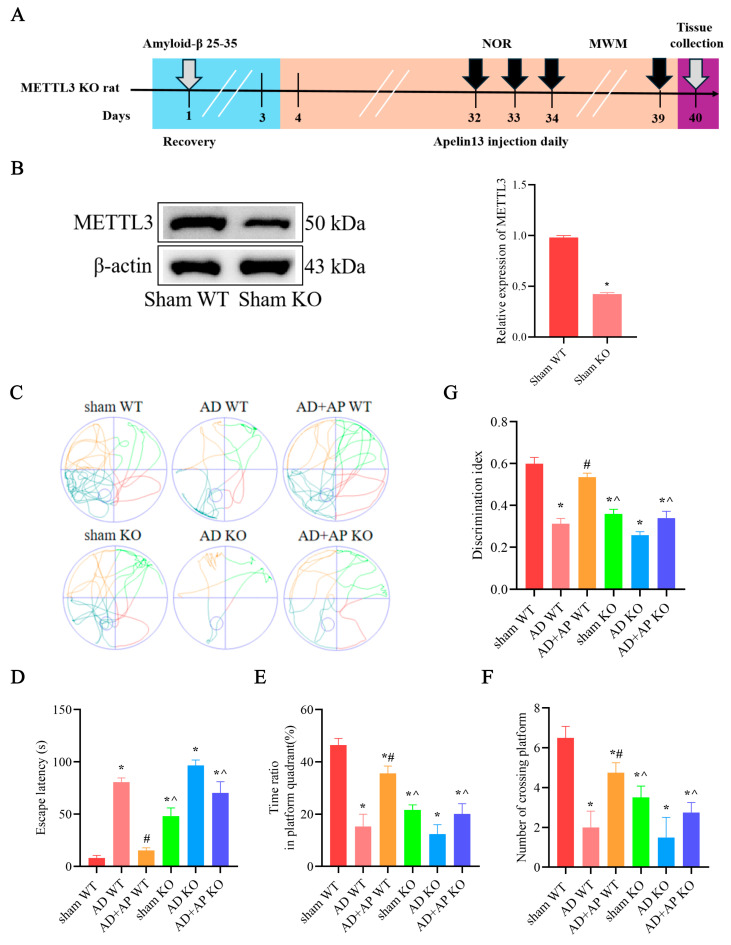
Effect of AP on cognitive function in METTL3 knockout rats. (**A**) A schematic illustration depicting the experimental designs and procedure. (**B**) Protein level of METTL3 detected by Western blotting. *n* = 3. The following are in the Morris water maze test: (**C**) Swimming trajectories of rats in Spatial exploration test. (**D**) Escape latency. (**E**) Time ratio in platform quadrant. (**F**) Number of crossing platform. (**G**) Discrimination index in Novel object recognition test. Original images can be found in Supplementary File S1. Data is expressed as mean ± SEM, *n* = 8, * *p* < 0.05 vs. sham WT, # *p* < 0.05 vs. AD WT, ^ *p* < 0.05 vs. AD+AP WT.

The timeline of experiments is shown in Figure 4A. Western blotting analysis showed that MTTL3 knockout rats have a marked decrease in MTTL3 expression in the hippocampus (*p* < 0.05, Figure 4B). In MWM test, the results revealed a longer escape latency (*F*(1, 28) = 7.11, *p <* 0.05) and fewer platform crossings (*F*(1, 28) = 6.025, *p <* 0.05) and a reduced time ratio in the platform quadrant (*F*(1, 28) = 6.375, *p <* 0.05, Figure 4C–F) in sham KO rats compairing with sham WT group, suggesting METTL3 may normally be involved in maintaining the homeostasis of cognitive function. There was no significant difference between the AD WT group and the AD KO group, as well as the AD+AP KO group and the sham KO group. AP treatment improved cognitive skills of AD WT rats (Escape latency: *F*(1, 28) = 4.496, *p <* 0.05; time ratio in platform quadrant: *F*(1, 28) = 4.573, *p* < 0.05; number of crossing platform: *F*(1, 28) = 4.311, *p <* 0.05, Figure 4C–F), while knockdown of METTL3 blocked this improvement effect (Escape latency: *F*(1, 28) = 5.947, *p <* 0.05. time ratio in platform quadrant: *F*(1, 28) = 5.152, *p* < 0.05. number of crossing platform: *F*(1, 28) = 4.978, *p <* 0.05, Figure 4C–F).

NOR test showed similar results. Sham KO rats showed decreased DI when compare with the sham WT group (*F*(1, 28) = 6.213, *p* < 0.05). No significant difference was found between the AD WT group and the AD KO group, as well as the AD+AP KO group and sham KO group. There was a significant decrease in DI in the AD+AP KO group when compared with the AD+AP group (*F*(1, 28) = 4.779, *p* < 0.05 and METTL3 knockout status × AP treatment, *F*(1, 28) = 5.012, *p <* 0.05, Figure 4G). Collectively, the above data indicated that METTL3 mediated AP-induced improvement of cognitive function in AD rats.

### 3.5. AP Effects on Neuropathological Changes in METTL3 Knockout Rats

**Figure 5:**
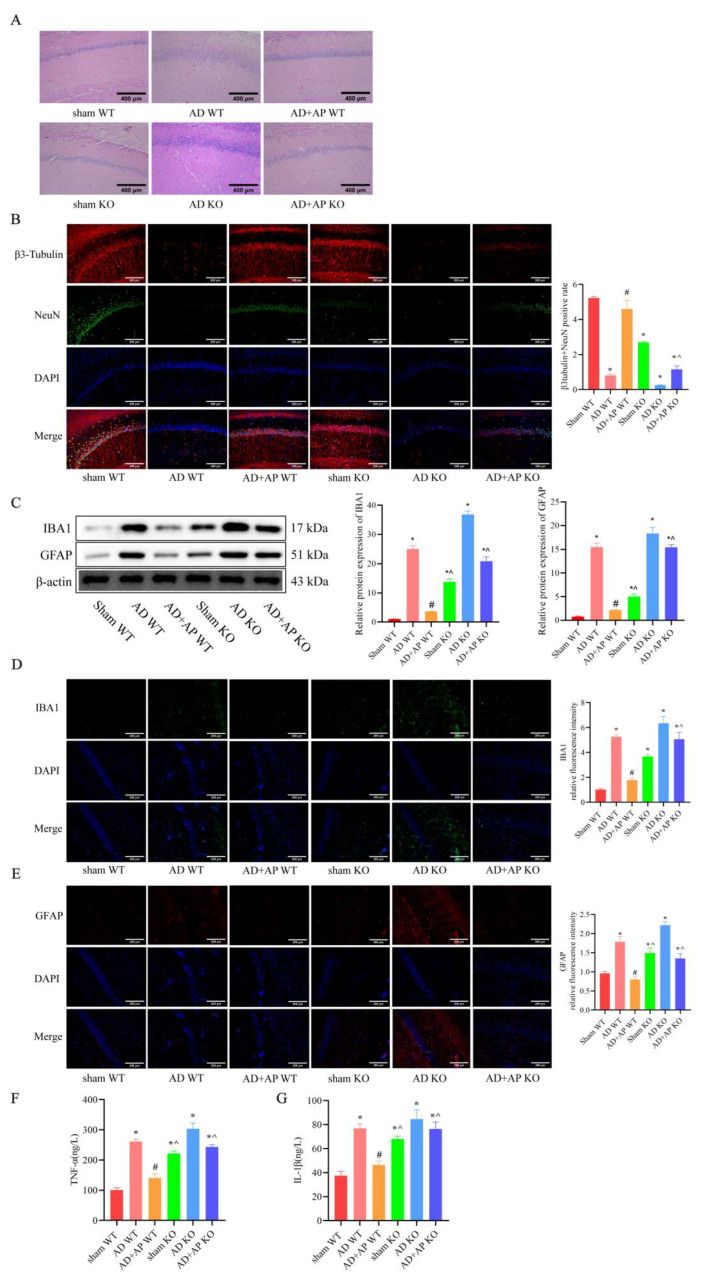
AP effects on Neuropathological changes in METTL3 Knockout Rats. (**A**) Representative images of H&E staining in the CA1 region of the hippocampus. Scale bar: 400 μm. (**B**) Representative immunofluorescence images of β3 tubulin (red) and NeuN(green) (neural markers) and DAPI staining(blue), and the graph shows the percentage of β3 tubulin and NeuN positive cells from 10 randomly chosen fields. *n* = 3, Scale bar: 200 μm (**C**) Protein levels of IBA 1 and GFAP detected by Western blotting, *n* = 3. (**D**) Representative immunofluorescence images of IBA 1 and its relative intensity. *n* = 3, Scale bar: 200 μm. (**E**) Representative immunofluorescence images of GFAP and its relative intensity. *n* = 3, Scale bar: 200 μm (**F**) Inflammatory factor TNF-α expression by ELISA. *n* = 6. (**G**) Inflammatory factor IL-1β expression by ELISA. *n* = 6. Original images can be found in Supplementary File S1. Data is expressed as mean ± SEM, * *p* < 0.05 vs. sham WT, ^#^ *p* < 0.05 vs. AD WT, ^ *p* < 0.05 vs. AD+AP WT.

The HE staining results revealed that, compared to the AD+AP WT group, the AD+AP KO group exhibited an increase in disorganized arrangement, cell shrinkage, nuclear consolidation, and deepened staining in hippocampal neurons. Sham KO rats showed significant nerve injury compared with the sham WT group. No significant difference was found between the AD WT group and the AD KO group (Figure 5A).

β3 tubulin and NeuN positive cells were significantly decrease in the AD+AP KO group compared to the AD+AP WT group (*F*(1, 8) = 5.143, *p* < 0.05 and METTL3 knockout status × AP treatment, *F*(1, 8) = 8.221, *p <* 0.05), with no significant differences between the AD+AP KO and AD WT groups. There was a significant neuron loss in sham KO rats when compared with the sham WT group (*F*(1, 8) = 7.998, *p <* 0.05). No significant difference was found between the AD WT group and AD KO group (Figure 5B).

The AD+AP KO group also had significantly higher levels of IBA1 (*F*(1, 8) = 5.143, *p* < 0.05 and METTL3 knockout status × AP treatment, *F*(1, 8) = 8.221, *p* < 0.05)), GFAP (*F*(1, 8) = 6.115, *p* < 0.05 and METTL3 knockout status × AP treatment, *F*(1, 8) = 7.134, *p* < 0.05), TNF-α (*F*(1, 18) = 4.779, *p* < 0.05 and METTL3 knockout status × AP treatment, *F*(1, 18) = 5.012, *p* < 0.05) and IL-1β (*F*(1, 18) = 5.298, *p* < 0.05 and METTL3 knockout status × AP treatment, *F*(1, 18) = 5.461, *p* < 0.05), indicating increased inflammation compared to the AD+AP WT group (*p* < 0.05), and no significant differences were observed between the AD+AP KO and AD WT groups sham KO rats showed higher IBA1(*F*(1, 8) = 7.998, *p* < 0.05), GFAP (*F*(1, 8) = 9.271, *p* < 0.05), TNF-α (*F*(1, 18) = 5.213, *p* < 0.05) and IL-1β (*F*(1, 18) = 6.331, *p* < 0.05) levels when compaired to the sham WT group. No significant difference was found between the AD WT group and the AD KO group (Figure 5C–G). These findings suggested that METTL3 mediated the effects of AP in ameliorating hippocampal nerve damage and neuroinflammation.

### 3.6. Effect of AP on lncRNA BDNF-AS and its Methylation and BDNF/TrkB Signaling Pathway in METTL3 Knockout Rats

**Figure 6:**
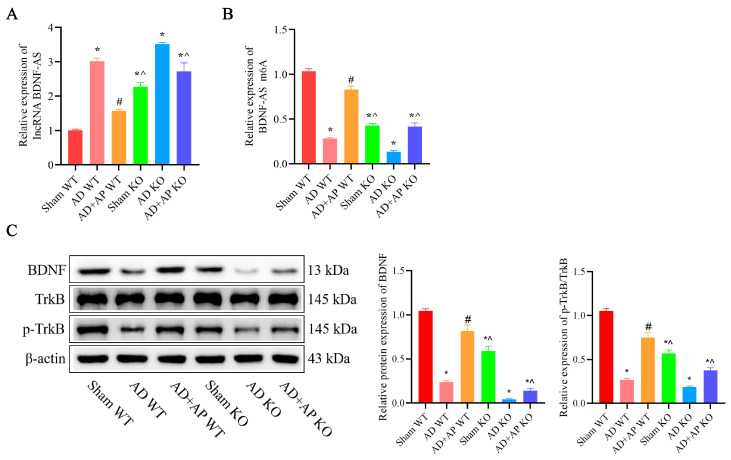
Effect of AP on lncRNA BDNF-AS and its methylation and BDNF/TrkB Signaling Pathway in METTL3 Knockout Rats. (**A**) RNA levels of *lncRNA BDNF-AS* detected by qRT-PCR. (**B**) RNA levels of *BDNF-AS m6A* detected by MeRIP-qPCR. (**C**) Protein levels of BDNF, TrkB, and p-TrkB detected by Western blotting. Original images can be found in Supplementary File S1. Data is expressed as mean ± SEM, *n* = 3, * *p* < 0.05 vs. sham WT, ^#^ *p* < 0.05 vs. AD WT, ^ *p* < 0.05 vs. AD+AP WT.

In the sham KO group, there was a significant upregulation of *lncRNA BDNF-AS* (*F*(1, 8) = 6.438, *p* < 0.05) and a remarkable downregulation of *BDNF-AS m6A* expression (*F*(1, 8) = 7.169, *p* < 0.05) compared to the sham WT group. The AD+AP KO group also had significantly higher levels of *lncRNA BDNF-AS* (*F*(1, 8) = 5.443, *p* < 0.05; METTL3 knockout status × AP treatment, *F*(1, 8) = 5.922, *p* < 0.05) and lower *BDNF-AS m6A* level compared with the AD+AP WT group (*F*(1, 8) = 5.992, *p* < 0.05; METTL3 knockout status × AP treatment, *F*(1, 8) = 6.421, *p* < 0.05), but no significant differences were observed between the AD+AP KO and AD WT groups (Figure 6A,B).

The levels of BDNF (*F*(1, 8) = 5.661, *p* < 0.05; METTL3 knockout status × AP treatment, *F*(1, 8) = 6.013, *p* < 0.05) and p-TrkB/TrkB (*F*(1, 8) = 5.401, *p* < 0.05; METTL3 knockout status × AP treatment, *F*(1, 8) = 5.637, *p* < 0.05) were significantly lower in the AD+AP KO group compared to the AD +AP WT group (*p* < 0.05), no significant difference between the AD+AP KO group and the AD KO group (Figure 6C).

### 3.7. AP Ameliorated Apoptosis and Neuroinflammation, Promoted Proliferation via METTL3/lnc RNA BDNF-AS/BDNF Pathway in an In Vitro Model of AD

**Figure 7:**
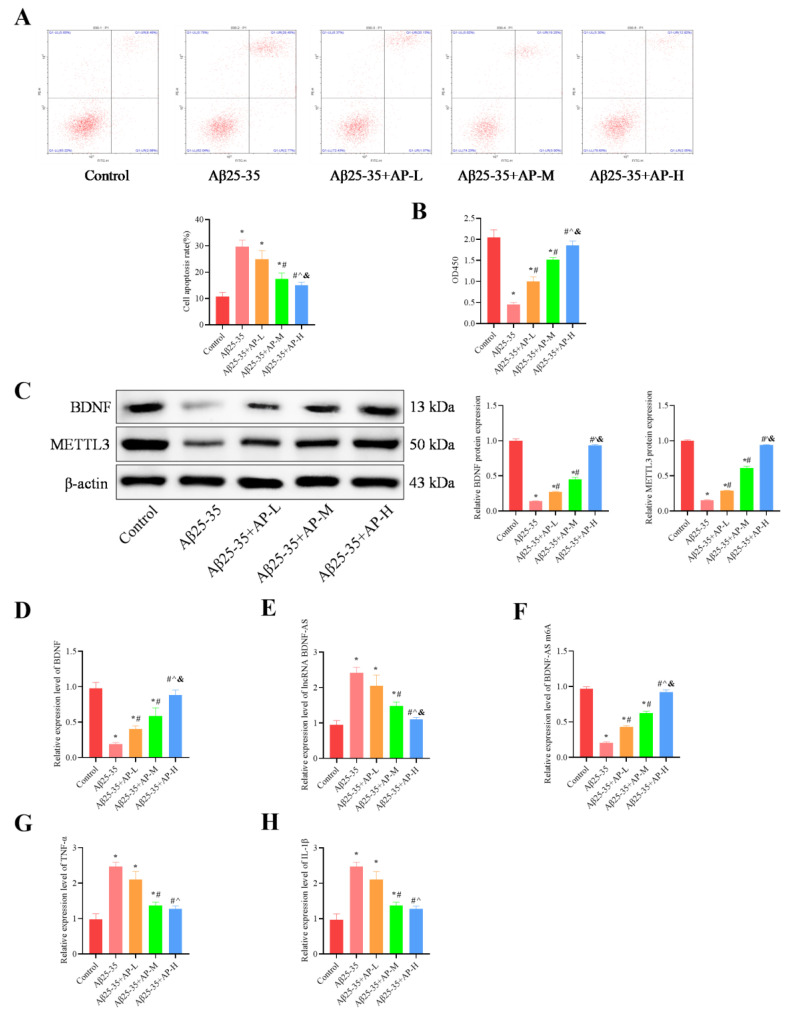
Effect of AP on cell proliferation, apoptosis, and neuroinflammation. (**A**) Flow cytometry for Cell apoptosis analysis. (**B**) MTT for evaluating Cell proliferation. (**C**) Protein level of BDNF and METTL3 detected by Western blotting. (**D**) RNA levels of *BDNF* detected by qRT-PCR. (**E**) RNA levels of *lncRNA BDNF-AS*, detected by qRT-PCR. (**F**) RNA levels of *BDNF-AS m6A* detected by MeRIP-qPCR. (**G**) Inflammatory factor *TNF-α* expression by qRT-PCR. (**H**) Inflammatory factor *IL-1β* expression by qRT-PCR. Original images can be found in Supplementary File S1. Data is expressed as mean ± SEM, *n* = 3, * *p <* 0.05 vs. Control, ^#^ *p <* 0.05 vs. Aβ25-35, ^ *p <* 0.05 vs. Aβ25-35+AP-L, & *p <* 0.05 vs. Aβ25-35+AP-M.

The results demonstrated that, compared with the control group, the Aβ25-35 group significantly increased cell apoptosis (*p* < 0.05, Figure 7A) and inhibited cell proliferation (*p* < 0.05, Figure 7B). AP treatment reversed these effects in a dose-dependent manner. Additionally, the Aβ25-35 group exhibited a considerable reduction in BDNF and METTL3 expression compared to the control group, which AP treatment also reversed in a dose-dependent manner (*p* < 0.05, Figure 7C,D). Furthermore, the Aβ25-35 group showed a significant decrease in *BDNF-AS m6A* expression, along with a notable increase in *lncRNA BDNF-AS* expression, when compared to the control group (*p* < 0.05). AP treatment effectively reversed these changes, with more substantial impacts at higher concentrations (*p* < 0.05, Figure 7E,F). The increased level of *TNF-α* and *IL-1β* induced by Aβ25-35 was dose-dependently inhibited by AP treatment (*p* < 0.05, Figure 7G,H). These findings indicate that AP can counteract Aβ25-35-induced pathological alterations through the METTL3/lnc RNA BDNF-AS/BDNF pathway.

## 4. Discussion

As AD advances, patients experience a decline in cognitive abilities, ultimately culminating in death [28]. Neuroinflammation may be a prerequisite for the development of hallmark AD features, such as synaptic loss and Aβ deposition [29,30,31]. Therefore, targeting inflammation, including the overactivation of microglia and astrocytes, is an effective strategy for treating AD. Our previous research found that apelin-13 inhibits neuroinflammation and improves the cognitive function of AD rats by upregulating the BDNF/TrkB signaling pathway [12]. However, the mechanism by which apelin-13 upregulates the BDNF/TrkB signaling pathway remains unclear. Prior studies have suggested that the suppression of lncRNA BDNF-AS, an antisense transcript of the BDNF gene, exerts a protective effect by negatively regulating BDNF. This suppression enhances the viability of PC12 cells damaged by Aβ25-35, and inhibits apoptosis and oxidative stress [32,33]. BDNF-AS knockdown also significantly suppressed inflammation and inflammatory pathway in AD model [34], while BDNF-AS induces neurotoxicity in AD [32]. These findings suggest that BDNF-AS may serve as a new pathologic gene for AD and a prognostic marker for AD patients. However, it remains unclear whether apelin-13 mediates upregulation of the BDNF/TrkB signaling pathway through regulating lncRNA BDNF-AS in AD. In this study, we firstly demonstrated that apelin-13 significantly enhanced BDNF/TrkB signaling pathway by inhibiting lncRNA BDNF-AS in AD.

Additionally, the level of m6A modification is decreased in AD [35]. However, no previous studies have reported the involvement of m6A modification in the regulation of BDNF-AS-mediated downregulation of BDNF in AD. Our research found that the methylation inhibitor DAA can reverse the apelin-13-mediated improvements in cognitive function impairment, as evidenced by improvements in performance on the Morris water maze and novel object recognition tasks. Furthermore, DAA administration also reverses apelin-13-mediated ameliorative effect on hippocampal nerve injury and loss, as well as microglial and astroglial hyperactivation and neuroinflammation in AD rats. These results suggest that apelin-13 improves the pathological changes in AD rats by upregulating m6A methylation levels. While Yang et al. demonstrated DAA-mediated suppression of TNFα/IL-1β in macrophages via NF-κB inhibition [36], our paradoxical findings in AD rats may be due to the following reasons: First, Tissue-specific effects: neuronal/glial methylation dynamics may differ from peripheral macrophages, a view supported by previous research [37]. Second, Apelin-13 crosstalk: DAA may disrupt apelin’s anti-inflammatory signaling in neurons. Further studies comparing cell-type-specific responses are warranted. Although DAA monotherapy was not experimentally assessed, its well-documented lack of intrinsic activity [19] and no worsening effect in AD+AP+DAA vs. AD WT groups support its antagonistic role. Future studies may validate this in DAA-only cohorts, though current evidence suggests minimal impact on measured outcomes. Further SRAMP data analysis revealed multiple high-confidence m6A modification sites on BDNF-AS, suggesting that this long non-coding RNA could be a key regulator in the context of AD. Our in vivo and in vitro experiments demonstrated that apelin-13 significantly upregulates BDNF-AS m6A methylation levels in a dose-dependent manner within AD models, resulting in a subsequent downregulation of BDNF-AS expression. These findings imply a critical relationship between apelin-13 and the m6A modification of BDNF-AS, potentially uncovering a novel pathway involved in the pathogenesis and progression of AD. Although our studies establish the ability of apelin-13 to modulate BDNF-AS m6A methylation, the precise molecular mechanisms underlying this regulatory effect necessitate further investigation.

The m6A modification is closely regulated by a suite of proteins: writers (methyltransferases), readers (binding proteins), and erasers (demethylases) [38]. Specifically, writer proteins catalyze the m6A methylation process [39]. Among them, METTL3 has been identified as the primary catalytic writer [40]. Notably, brains from patients with AD exhibit decreased expression of METTL3 [41], and METTL3 inhibition-induced m6A dysregulation exacerbates neurodegeneration in AD [42]. Consistently, we observed decreased METTL3 expression in in vivo and in vitro models of AD. AP dose-dependently upregulated METTL3, subsequently enhanced m6A modification, and reduced BDNF-AS expression. This further upregulated the BDNF/TrkB signaling pathway, inhibited neuroinflammation, and improved cognitive function. However, recent research showed that METTL3 protein is significantly upregulated in inflammatory microglia, aggravating the neuroinflammatory response [43]. This discrepancy may arise from the differential effects of METTL3 on neuroinflammation across various disease contexts. Contrastingly, another research showed that METTL3 inhibited microglial activation, M1 polarization and BBB permeability, thereby providing protection against cerebral ischemic stroke [37]. The findings indicated that METTL3 was involved in inflammation inhibition induced by AP through m6A modification of BDNF-AS in AD. Given that METTL3 diminishes RNA stability via m6A modification [44,45], we speculated that METTL3 reduced the stability of BDNF-AS through an m6A-dependent mechanism, thereby enhancing the BDNF/TrkB signaling pathway. In our study, we identified a noteworthy phenomenon wherein the administration of DAA inhibits the apelin-13-induced upregulation of METTL3. The potential mechanisms underlying this observation are twofold. First, DAA may inhibit the methylation of transcription factors (e.g., STAT3), reducing their ability to bind to the METTL3 promoter and suppressing its expression [46,47]. Second, DAA-induced global methylation inhibition may lead to a reduction in histone activation marks (H3K4me3) and a subsequent compression of the chromatin open regions associated with the METTL3 gene, ultimately leading to decreased expression of the METTL3 gene [48,49]. For the inverse relationship between apelin-13 (reduced in AD serum) and APJ (elevated in AD brains), it may suggest compensatory receptor upregulation. Our previous research demonstrated a decrease in both apelin-13 and APJ in the brains of AD rats [12]. The mechanism by which apelin-13/APJ regulates METTL3 expression remains unexplored. We hypothesize that APJ upregulates METTL3 through transcriptional regulation: APJ/Gαi signaling may enhance CREB phosphorylation or activate transcription factors such as STAT3, which in turn activate the METTL3 promoter [50]. This inference requires further experimental verification.

## 5. Conclusions

**Figure 8:**
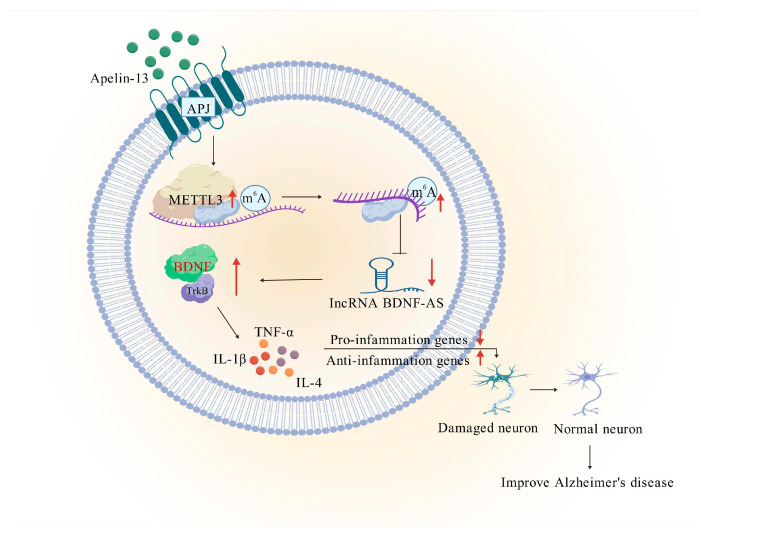
Mechanical diagram. Apelin-13-mediated upregulation of METTL3 downregulates lncRNA BDNF-AS, which enhances BDNF/TrkB signaling pathway to inhibit neuroinflammation, thus ameliorating Alzheimer’s disease. The red upward arrow indicates an increase in expression. And the downward arrow indicates a decrease.

We have demonstrated for the first time that AP-mediated upregulation of METTL3 alleviates AD by enhancing the BDNF/TrkB pathway and inhibiting inflammation through an m6A-dependent reduction in BDNF-AS transcripts (Figure 8). Our results offer fresh insights into the mechanisms regulating neuroinflammation in AD and present promising drug targets for its treatment.

## Figures and Tables

**Table 1:** PCR primers used in this study.

Gene	Direction	Sequence (5′–3′)
*BDNF*	F	ATGACCATCCTTTTCCTTCATCTTC
	R	TTTCCTTGTTGGGACGTTTGT
*lncRNA BDNF-AS*	F	GAGTCATCGTCAGGCCTTTC
	R	AGTTGGCAGTGTCCTGGAGT
*lncRNA BDNF-AS m6A*	F	GGAAAGGGAAGAGCTGGGAC
	R	TCACACAGGAAGTCAGGAAC
*IL-1β*	F	TCGGCCAAGACAGGTCGCTCA
	R	TGGTTGCCCATCAGAGGCAAGG
*TNF-α*	F	TCATTCCTGCTCGTGGCGGG
	R	CGGCTGACGGTGGGGTGAG
*β-actin*	F	AGAGCTACGAGCTGCCTGAC
	R	AGCACTGTGTTGGCGTACAG

## Data Availability

The data presented in this study are available from the corresponding author upon request.

## Supplementary Materials

The following supporting information can be downloaded at: https://www.mdpi.com/article/10.3390/biom15081188/s1, Figure S1: AlzData database analyzed BDNF expression in AD patients; Figure S2: StarBase database analyzed BDNF-AS m6A methylation site; Figure S3: AlzData database analyzed APJ expression in early-stage AD patients; File S1: Original Western Blot Images.

## Abbreviations

The following abbreviations are used in this manuscript:

AP	Apelin-13
APLN	Apelin
AD	Alzheimer’s disease
BDNF	Brain-derived neurotrophic factor
TrkB	tyrosine kinase receptor B
BDNF-AS	Brain-derived neurotrophic factor antisense RNA
lncRNA	long non-coding RNA
m6A	N6-adenylate methylation
METTL3	methyltransferase-like 3
APJ	angiotensin II type 1 receptor (AT1) -related protein
SD	Sprague-Dawley
KO	METTL3 knockout rats
NOR	Novel Object Recognition Test
MWM	Morris Water Maze
MeRIP-qPCR	Methylated RIP-qPCR
