# Supplementary material for: Apelin-13-Mediated Upregulation of METTL3 Ameliorates Alzheimer’s Disease via Inhibiting Neuroinflammation Through m6A-Dependent Regulation of lncRNA BDNF-AS
Source: Biomolecules. 2025 Aug 18;15(8):1188. doi: 10.3390/biom15081188 (PMC12384069; doi:10.3390/biom15081188)
Supplement: Supplementary file 1 [file biomolecules-15-01188-s001.zip › biomolecules-3675839-supplementary figures.pdf]

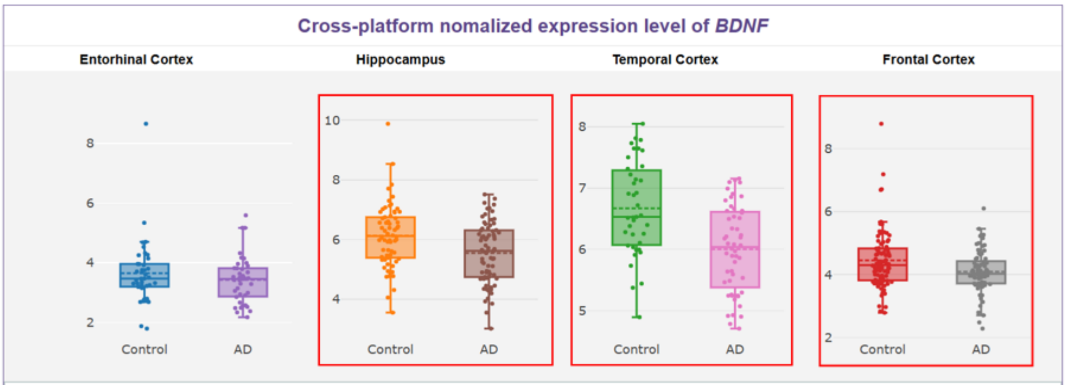


BDNF: brain-derived neurotrophic factor

| Brain region | log2 FoldChange | P-value | FDR |
|---|---|---|---|
| Entorhinal Cortex | -0.24 | 0.28 | 0.468 |
| Hippocampus | -0.64 | 0.000421011070852949 | 0.012 |
| Temporal Cortex | -0.65 | 7.09563322538288e-05 | 0.001 |
| Frontal Cortex | -0.38 | 0.001 | 0.007 |

Figure S1: Expression differences of BDNF between AD and normal populations.

In this study, we investigated the differential expression of genes across various brain regions associated with neurological conditions. The datasets analyzed were sourced from the National Center for Biotechnology Information (NCBI) Gene Expression Omnibus (GEO) database, encompassing the Entorhinal Cortex, Hippocampus, Temporal Cortex, and Frontal Cortex. The Entorhinal Cortex datasets included GSE26927, GSE26972, GSE48350, and GSE5281; the Hippocampus datasets comprised GSE28146, GSE29378, GSE36980, GSE48350, and GSE5281; the Temporal Cortex datasets included GSE29652, GSE36980, GSE37263, and GSE5281; and the Frontal Cortex datasets consisted of GSE12685, GSE36980, GSE48350, GSE5281, GSE53890, and GSE66333.

we conducted a comprehensive analysis of the datasets in human AD vs. control brains from alzdata (http://www.alzdata.org/). The demographic profile of the population analyzed is as follows: Total samples: n = 589 (AD:274; controls: 315); Age: 73.2 ± 19.1 years (mean ± SD); Sex: 52.5% female (AD group: 51.4% F; controls: 48.6% F). All differential expression results were adjusted for the age and sex of the samples. Data were filtered, background corrected, log2 transformed, and normalized prior to analysis. The criteria for identifying differentially expressed genes (DEGs) were set at |log2 Fold Change (FC)| > 1 and a p-value < 0.05, with results indicating significant differential expression observed specifically in the Hippocampus (log2 FC

= -0.64, p = 0.00042, FDR = 0.012) and Temporal Cortex (log2 FC = -0.65, p = 7.1e-05, FDR = 0.001).

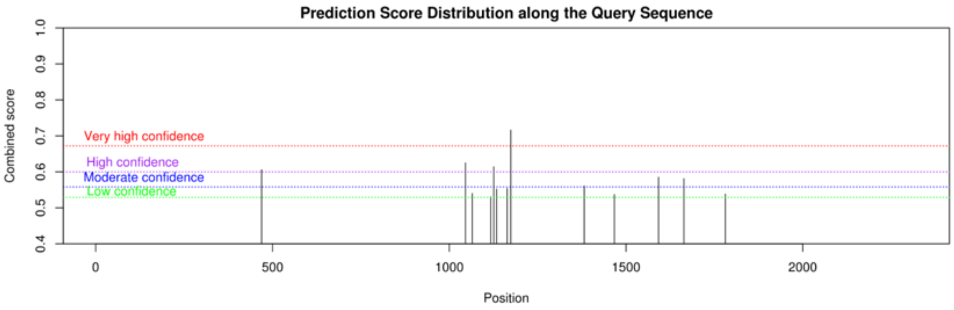


| # | Position | Sequence context | Structural context | Local structure visualization | Score(binary) | Score(knn) | Score(spectrum) | Score(combined) | Decision |
|---|---|---|---|---|---|---|---|---|---|
| 1 | 469 | AUUGU UUUCU UCAUU<br>GGGCC GAACU UUCUG<br>GUCCU CAUCC AACAG | IIPPP PPMMM PPPII<br>IIPPP MMMPP PPPPP<br>PPMMM MMPPP MMMMP | Draw | 0.606 | 0.324 | 0.642 | 0.606 | $m^6A$ site (High confidence) |
| 2 | 1046 | GUGGC UAAGG GGCUA<br>AGAUG UGACU UGAAA<br>AGAAA GGUAG AACAA | IIPPP PPIPP PPPPM<br>MMMMP PPBPB PPPPP<br>PPPPP PPPMM MMMMP | Draw | 0.521 | 0.376 | 0.799 | 0.625 | $m^6A$ site (High confidence) |
| 3 | 1065 | GUGAC UUGAA AAGAA<br>AGGUA GAACA AGAUC<br>AUCUC AAAUU UAUUA | PPPBP BPPPP PPPPP<br>PPPPM MMMMM PPPPP<br>IPPPP PIIII IIIII | Draw | 0.377 | 0.231 | 0.803 | 0.540 | $m^6A$ site (Low confidence) |
| 4 | 1117 | AUAGU UCAGA AAACG<br>ACUUC AGACC ACAGA<br>GACAG CAGAA CAGAU | PMMPP PPMPP PHHHH<br>HHPPP MMMMM MMMMM<br>PPPPI IIPPP PPPPP | Draw | 0.411 | 0.317 | 0.722 | 0.531 | $m^6A$ site (Low confidence) |
| 5 | 1126 | AAAAC GACUU CAGAC<br>CACAG AGACA GCAGA<br>ACAGA UGGUC CGGCA | PPHHH HHHPP PMMMM<br>MMMMM MPPPP IIIPP<br>PPPPP PPPBP PMMPP | Draw | 0.546 | 0.387 | 0.738 | 0.614 | $m^6A$ site (High confidence) |
| 6 | 1134 | UUCAG ACCAC AGAGA<br>CAGCA GAACA GAUGG<br>UCCGG CAUGG AUAGA | PPPMM MMMMM MMMPP<br>PPIII PPPPP PPPPP<br>BPPII PPPPI PPPPI | Draw | 0.444 | 0.404 | 0.720 | 0.552 | $m^6A$ site (Low confidence) |
| 7 | 1164 | UCCGG CAUGG AUAGA<br>GCAUC AGACA CUCAC<br>AGACU GUGCC AACAA | BPPII PPPPI PPPPM<br>MMMMM MMMPP PPIPP<br>IIIIP PPPPP PIIIP | Draw | 0.456 | 0.600 | 0.685 | 0.554 | $m^6A$ site (Low confidence) |
| 8 | 1174 | AUAGA GCAUC AGACA<br>CUCAC AGACU GUGCC<br>AACAA GAGCC AUCGA | PPPPI IIIII IPPPP<br>PPPIP PPIIP PPPPM<br>MMPPP PPPII IIIII | Draw | 0.753 | 0.633 | 0.676 | 0.716 | $m^6A$ site (Very high confidence) |

Figure S2: Analysis of m6A methylation modification sites of BDNF-AS using the SRAMP database.

The analysis of N6-methyladenosine (m6A) modification sites was performed using the Sequence-based RNA Adenosine Methylation Site Predictor (SRAMP) (http://www.cuilab.cn/sramp). The m6A sites were evaluated based on their sequence context, structural features, and multiple scoring metrics, including binary scoring, k-nearest neighbors (KNN), spectral analysis, and a combined score. A total of 13 candidate m6A sites were identified, categorized by their confidence levels.

The prediction results indicate that sites 1, 2, 5, and 8 are classified as high confidence, while several others show lower confidence levels. This analysis highlights the potential sequence and structural determinants of m6A modifications, which could inform future research on their biological implications.

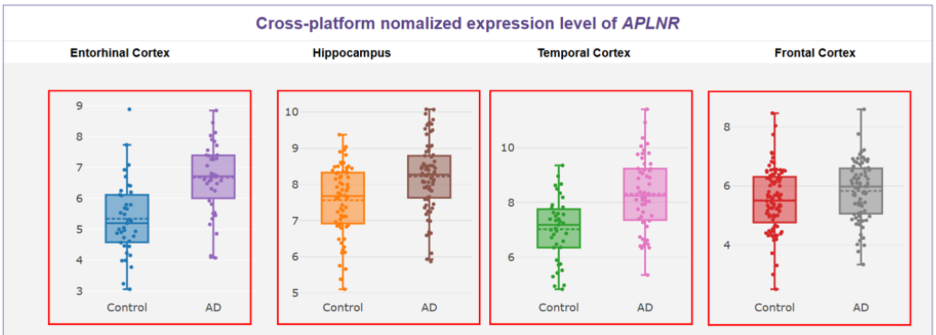


APLNR: apelin receptor

| Brain region | log2 FoldChange | P-value | FDR |
|---|---|---|---|
| Entorhinal Cortex | 1.35 | 1.55102406555381e-06 | 0.001 |
| Hippocampus | 0.61 | 0.000274970506586381 | 0.01 |
| Temporal Cortex | 1.3 | 4.41832939908563e-06 | 0.000257847034554186 |
| Frontal Cortex | 0.37 | 0.008 | 0.043 |

Figure S3: Expression differences of APLNR between AD and normal populations.

In this study, we focused on the apelin receptor (APLNR) and its differential expression across various brain regions relevant to neurological conditions. The datasets analyzed were sourced from the National Center for Biotechnology Information (NCBI) Gene Expression Omnibus (GEO) database, specifically targeting the Entorhinal Cortex, Hippocampus, Temporal Cortex, and Frontal Cortex. The datasets for the Entorhinal Cortex included GSE26927, GSE26972, GSE48350, and GSE5281; the Hippocampus datasets comprised GSE28146, GSE29378, GSE36980, GSE48350, and GSE5281; the Temporal Cortex datasets included GSE29652, GSE36980, GSE37263, and GSE5281; and the Frontal Cortex datasets consisted of GSE12685, GSE36980, GSE48350, GSE5281, GSE53890, and GSE66333.

we conducted a comprehensive analysis of the datasets from alzdata (http://www.alzdata.org/). All differential expression results were adjusted for the age and sex of the samples. All differential expression results were adjusted for age and sex of samples. The analysis revealed significant upregulation of APLNR in the following brain regions: Entorhinal Cortex (log2 Fold Change = 1.35, p = 1.55e-06, FDR = 0.001), Hippocampus (log2 Fold Change = 0.61, p = 0.00027, FDR = 0.01), Temporal Cortex (log2 Fold Change = 1.3, p = 4.42e-06, FDR = 0.00026), and Frontal Cortex (log2 Fold Change = 0.37, p = 0.008, FDR = 0.043). These findings suggest a potential role of APLNR in the pathophysiology of neurological disorders.
