# Supplementary figures and images for: Apelin-13-Mediated Upregulation of METTL3 Ameliorates Alzheimer’s Disease via Inhibiting Neuroinflammation Through m6A-Dependent Regulation of lncRNA BDNF-AS
Source: Biomolecules. 2025 Aug 18;15(8):1188. doi: 10.3390/biom15081188 (PMC12384069; doi:10.3390/biom15081188)

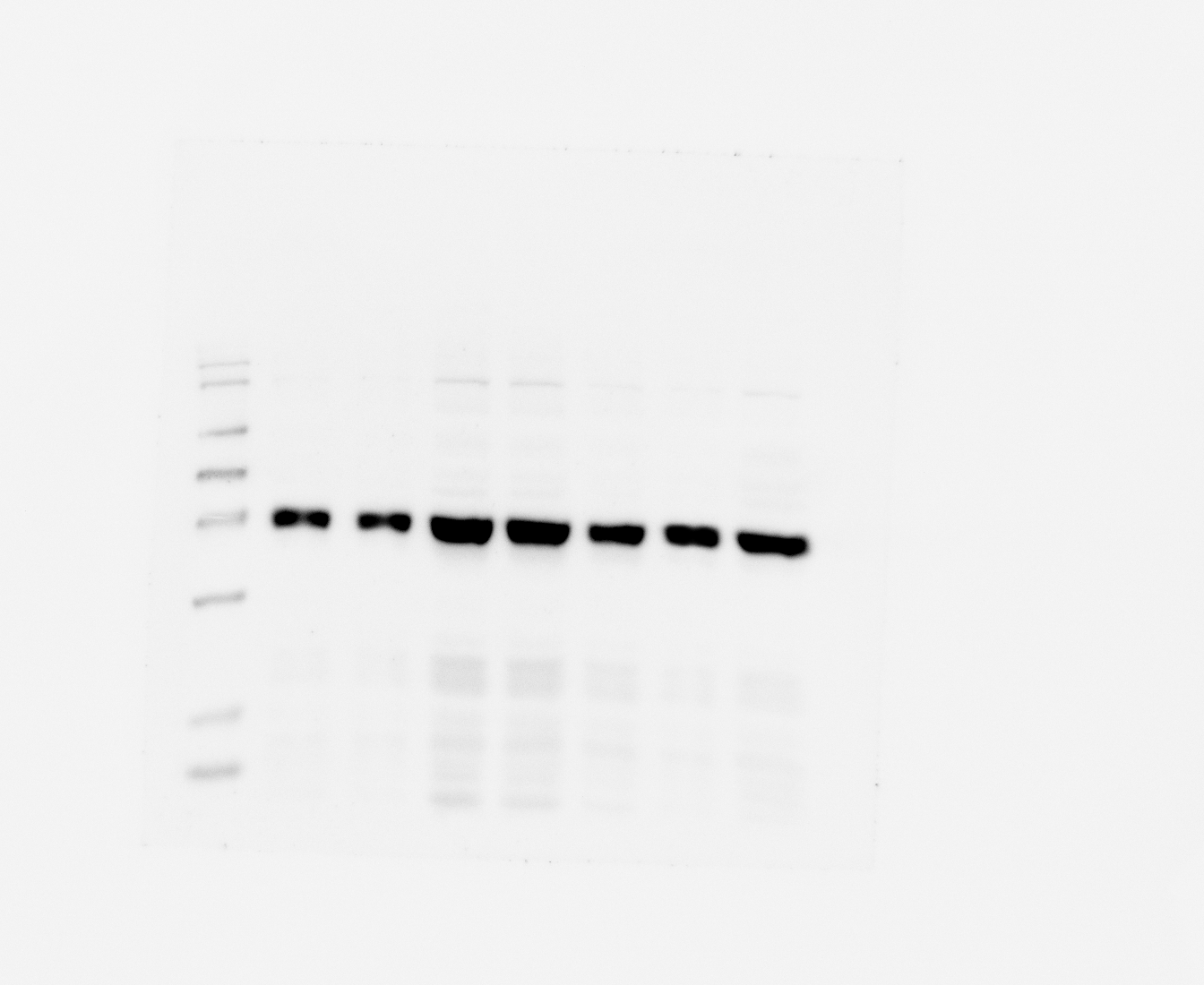

Supplement: Supplementary file 1 [file biomolecules-15-01188-s001.zip › File S1. Original Images for Blots/Figure2 C/1-GFAP-1.tif]

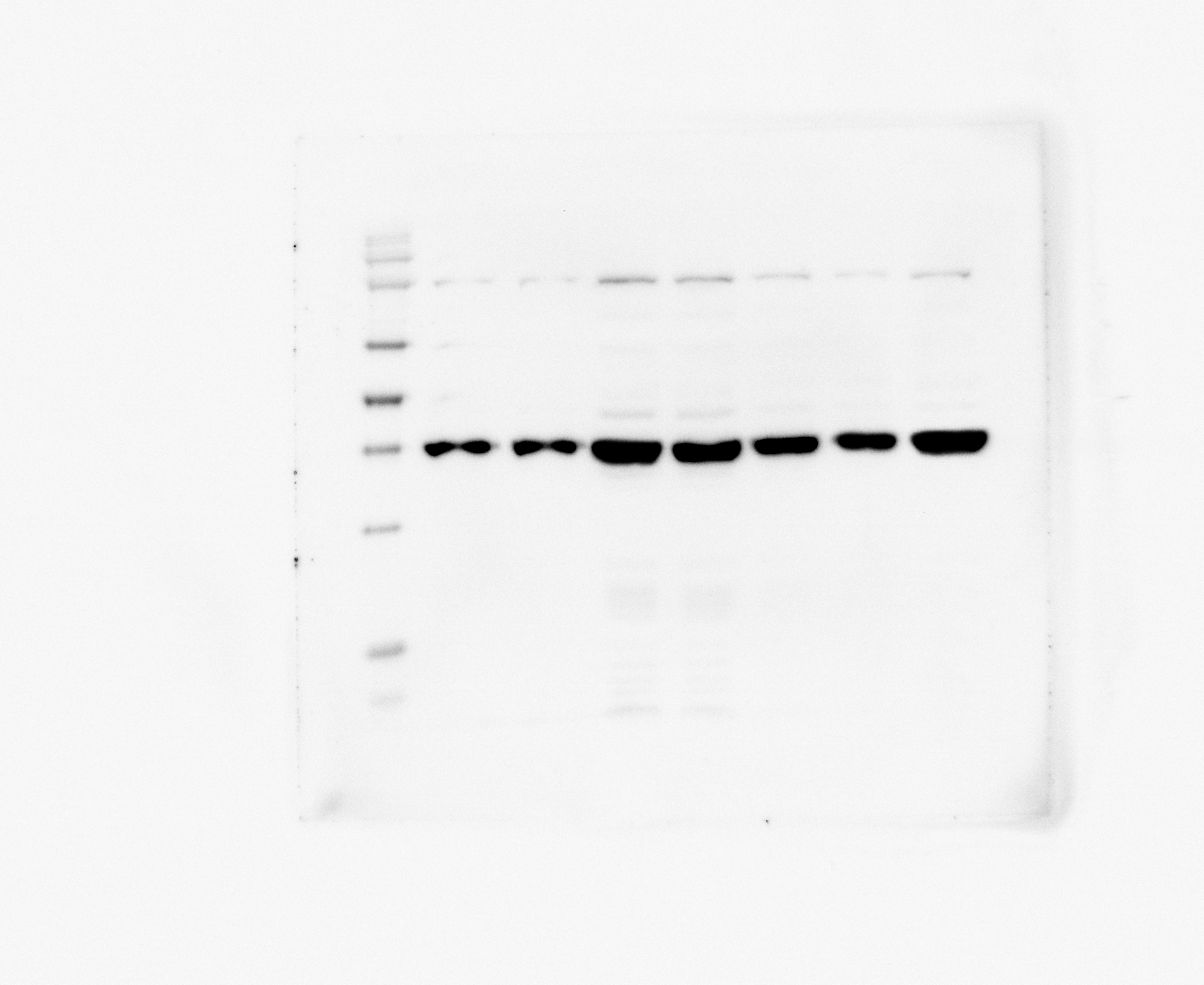

Supplement: Supplementary file 1 [file biomolecules-15-01188-s001.zip › File S1. Original Images for Blots/Figure2 C/1-GFAP-2 报告.tif]

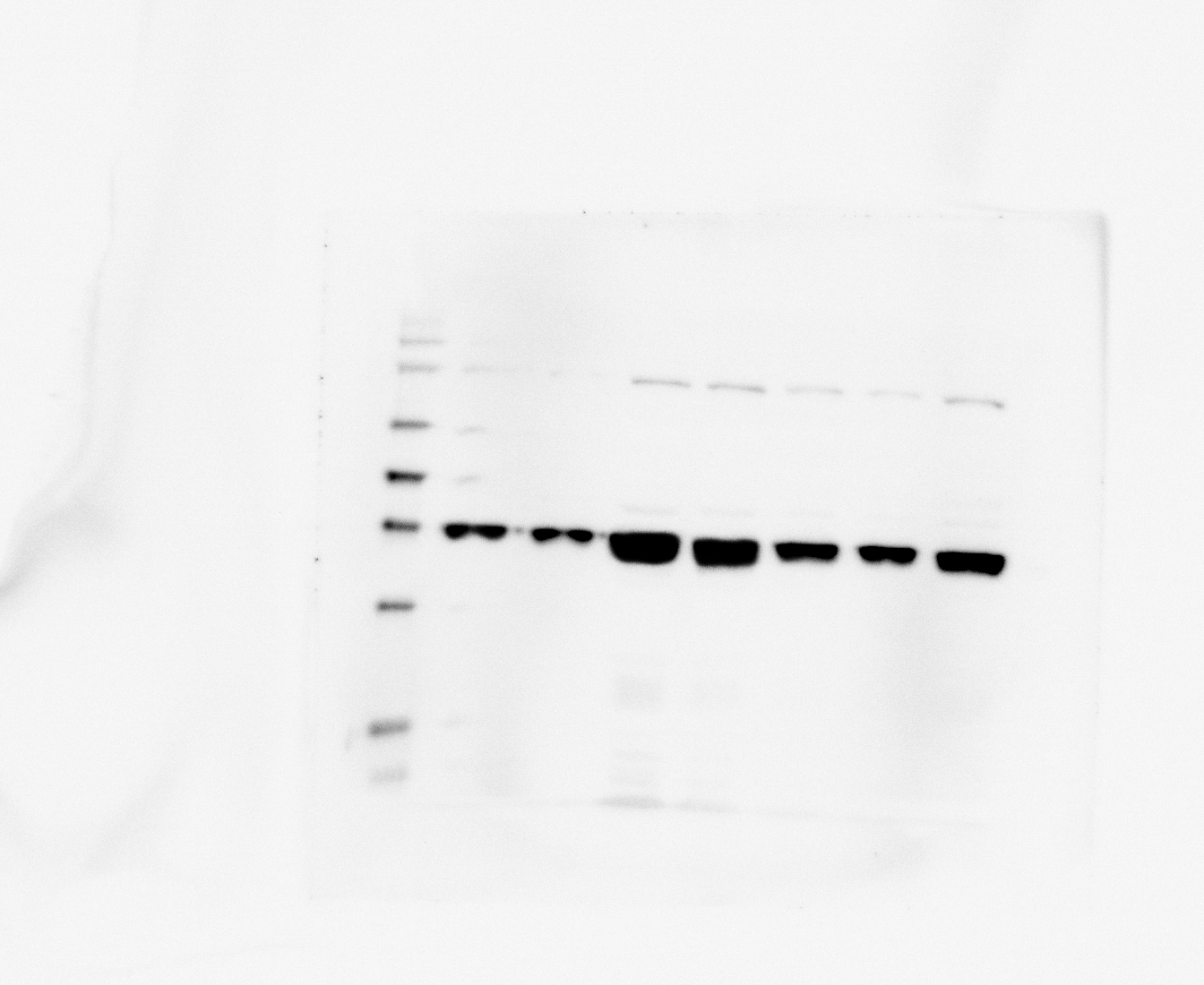

Supplement: Supplementary file 1 [file biomolecules-15-01188-s001.zip › File S1. Original Images for Blots/Figure2 C/1-GFAP-3.tif]

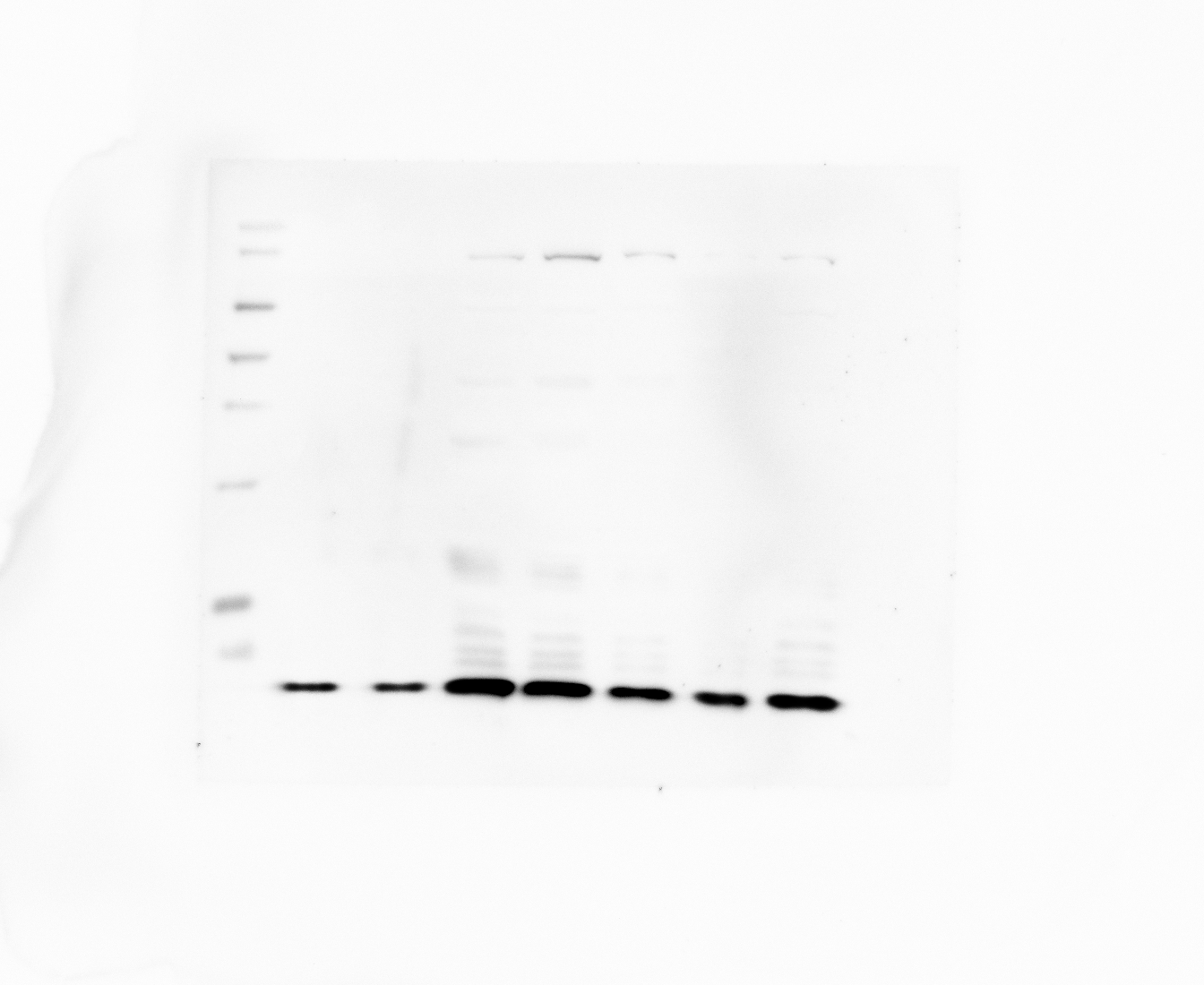

Supplement: Supplementary file 1 [file biomolecules-15-01188-s001.zip › File S1. Original Images for Blots/Figure2 C/1-IBA1-1 报告.tif]

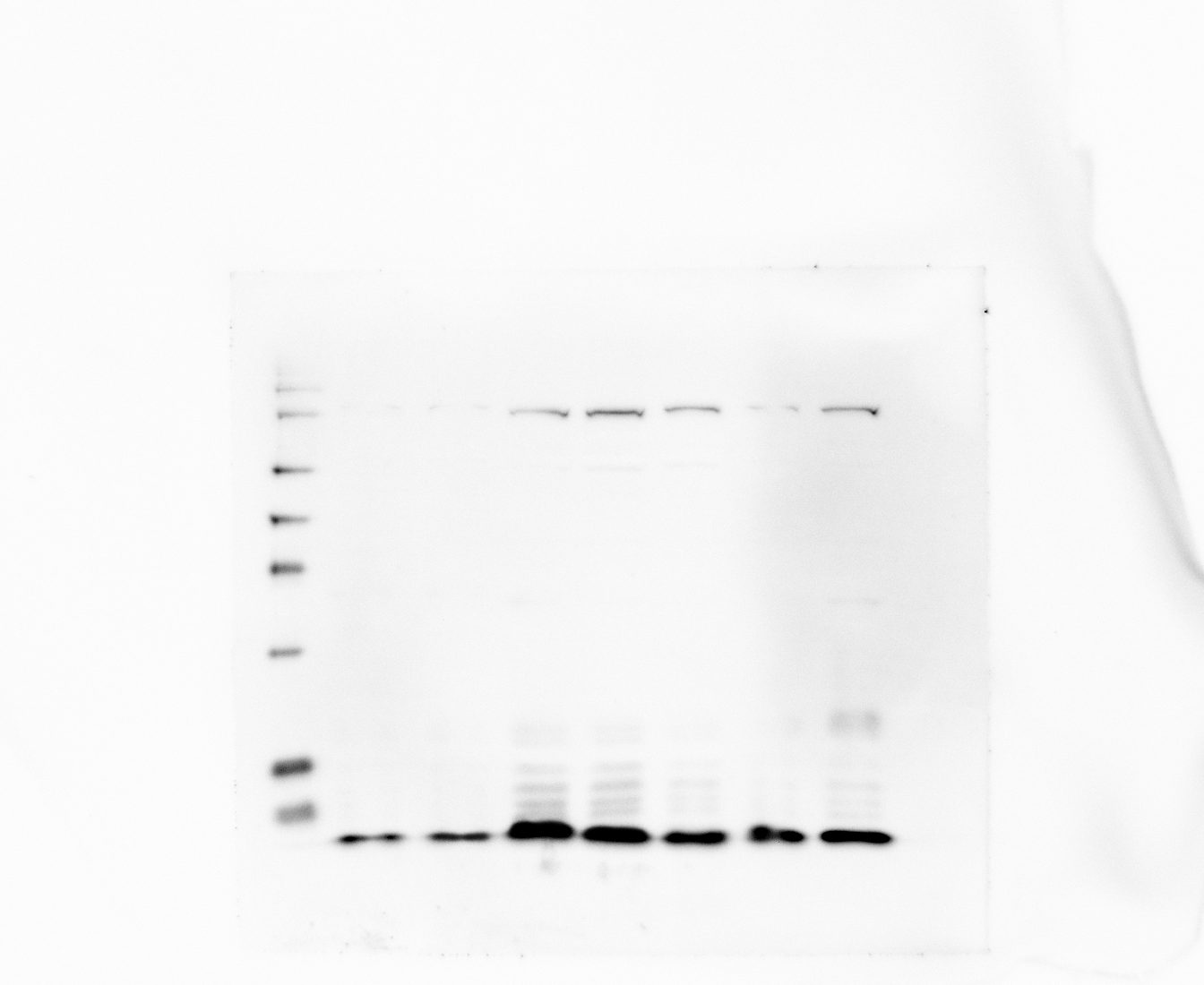

Supplement: Supplementary file 1 [file biomolecules-15-01188-s001.zip › File S1. Original Images for Blots/Figure2 C/1-IBA1-2.tif]

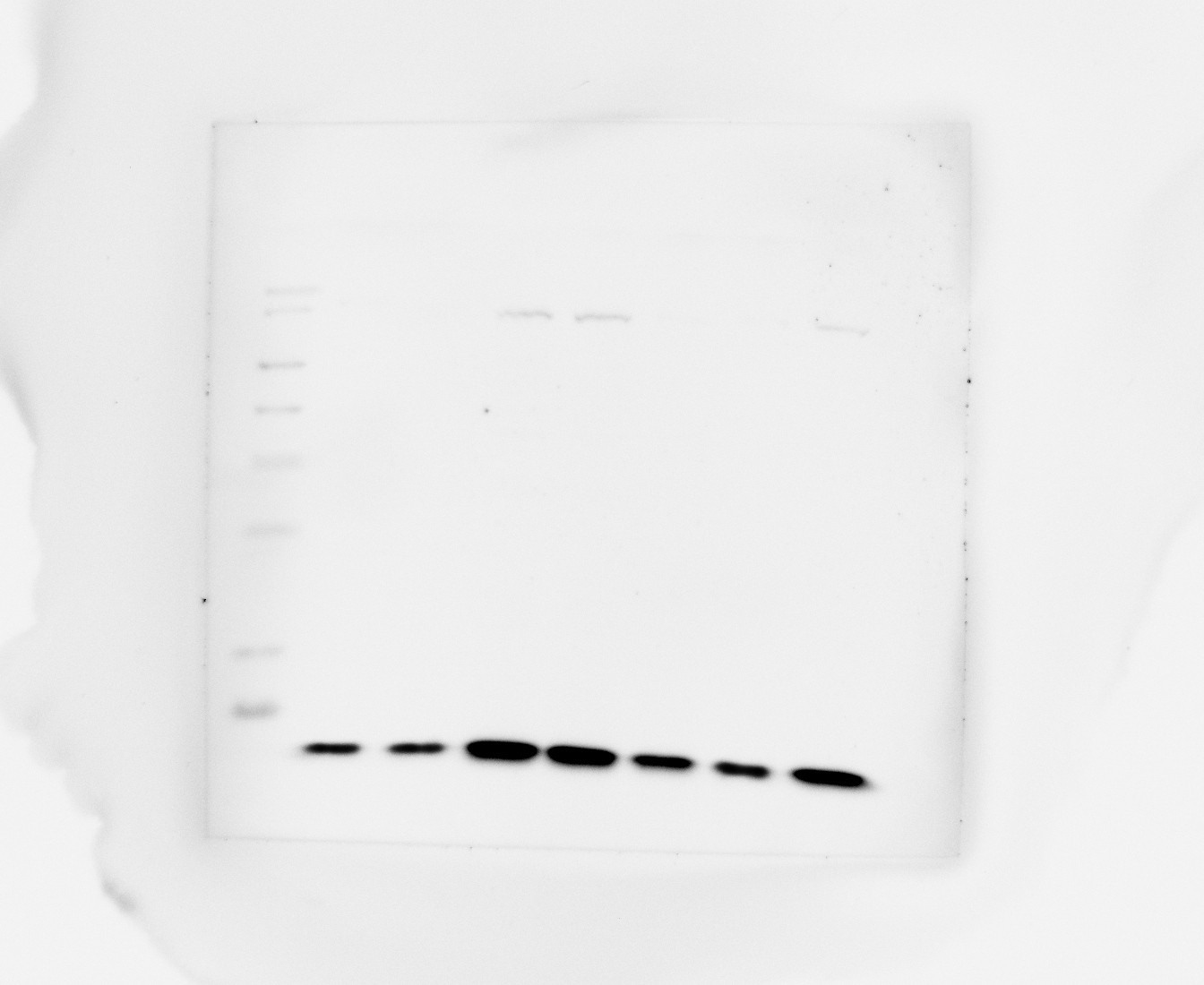

Supplement: Supplementary file 1 [file biomolecules-15-01188-s001.zip › File S1. Original Images for Blots/Figure2 C/1-IBA1-3.tif]

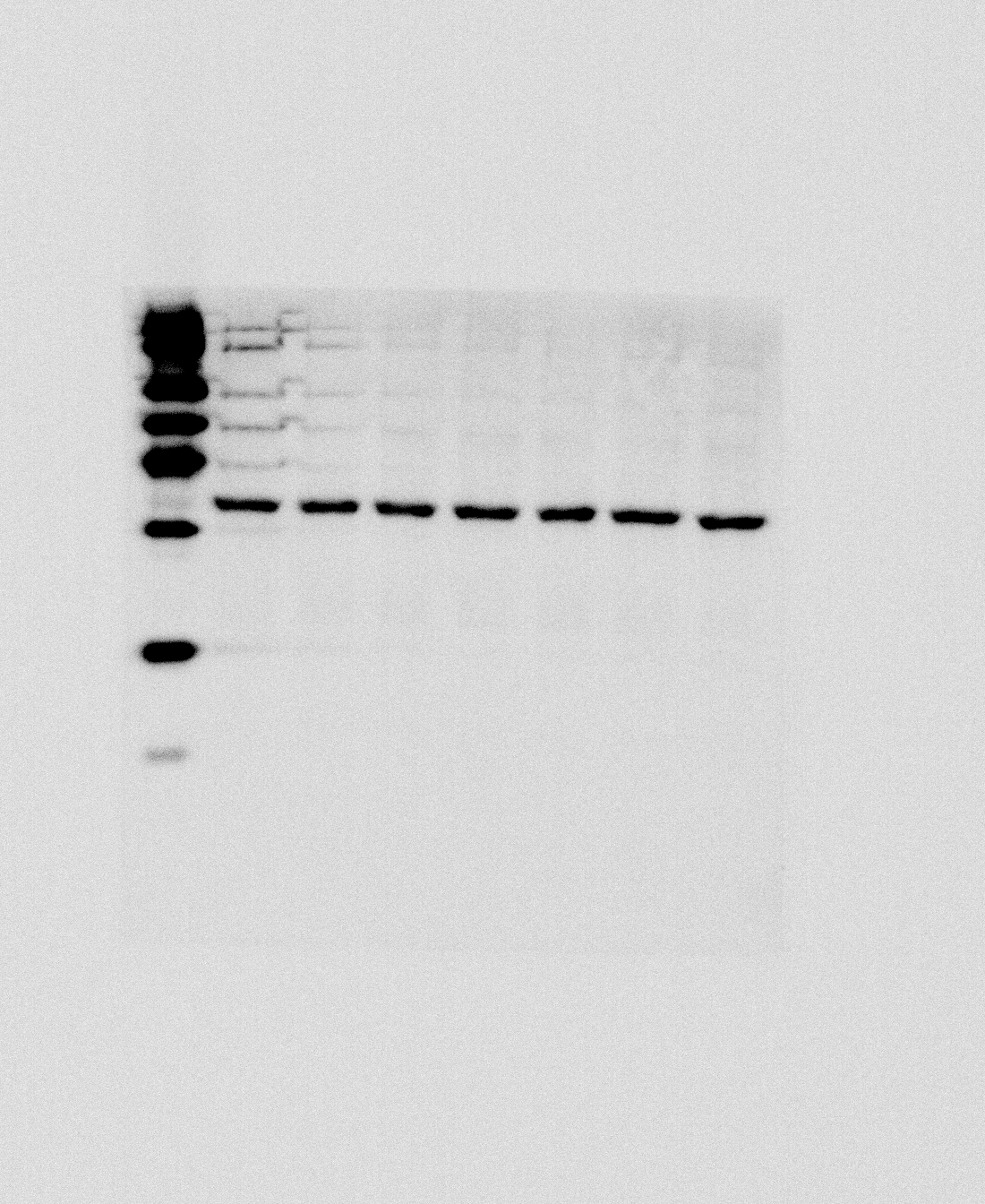

Supplement: Supplementary file 1 [file biomolecules-15-01188-s001.zip › File S1. Original Images for Blots/Figure2 C/1-β-actin-1.tif]

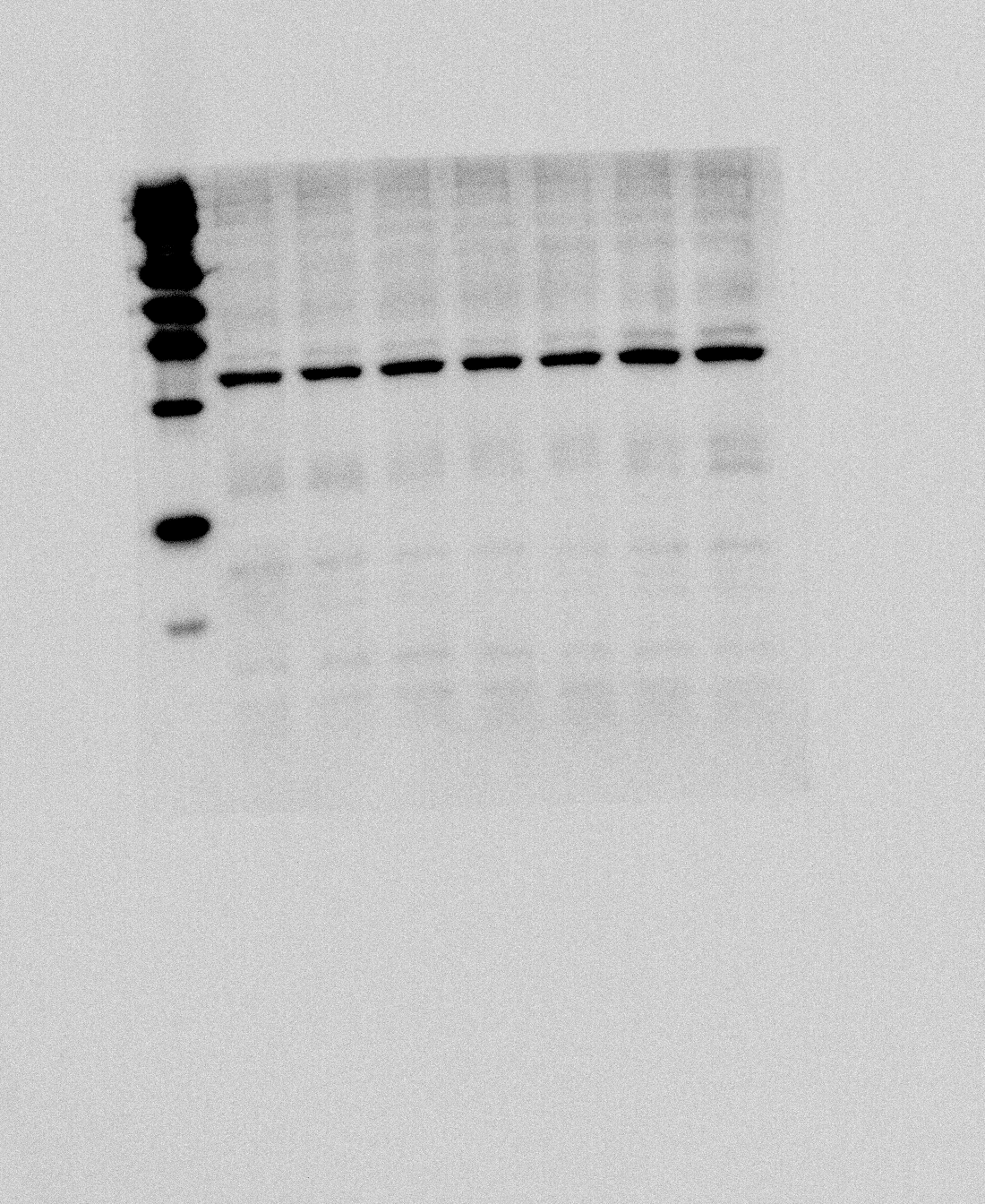

Supplement: Supplementary file 1 [file biomolecules-15-01188-s001.zip › File S1. Original Images for Blots/Figure2 C/1-β-actin-2 报告.tif]

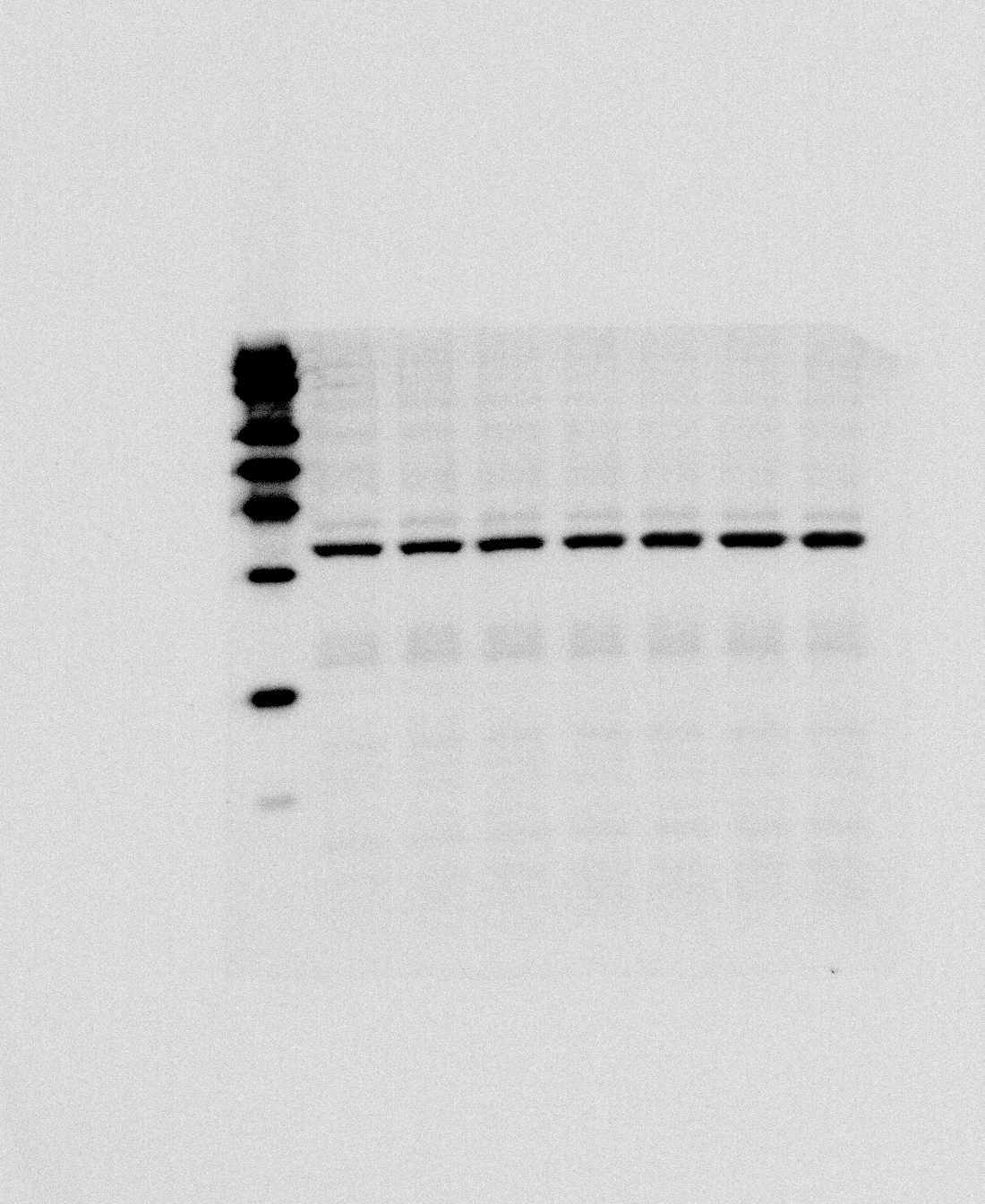

Supplement: Supplementary file 1 [file biomolecules-15-01188-s001.zip › File S1. Original Images for Blots/Figure2 C/1-β-actin-3.tif]

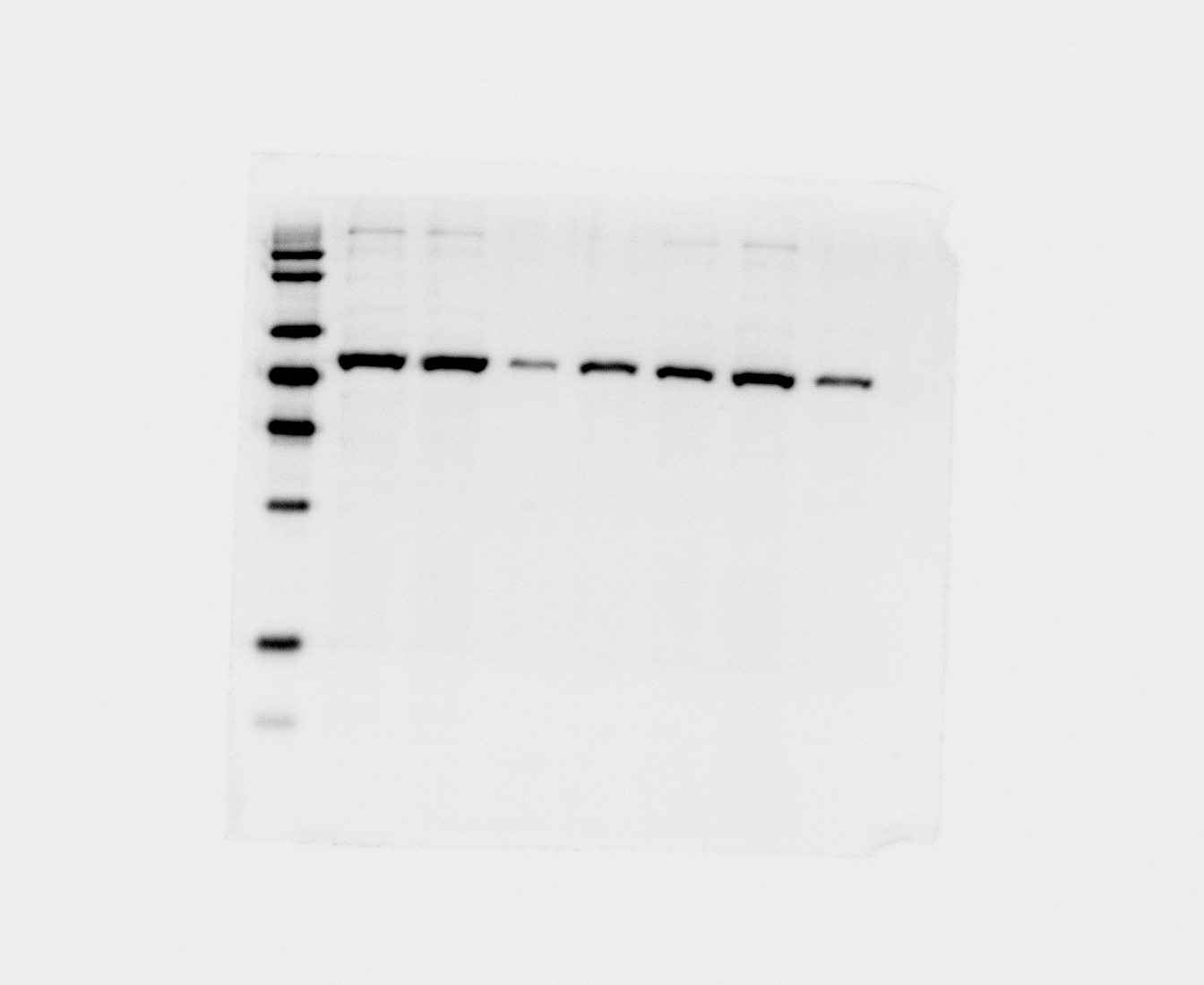

Supplement: Supplementary file 1 [file biomolecules-15-01188-s001.zip › File S1. Original Images for Blots/Figure3 C/2-METTL3-1 报告.tif]

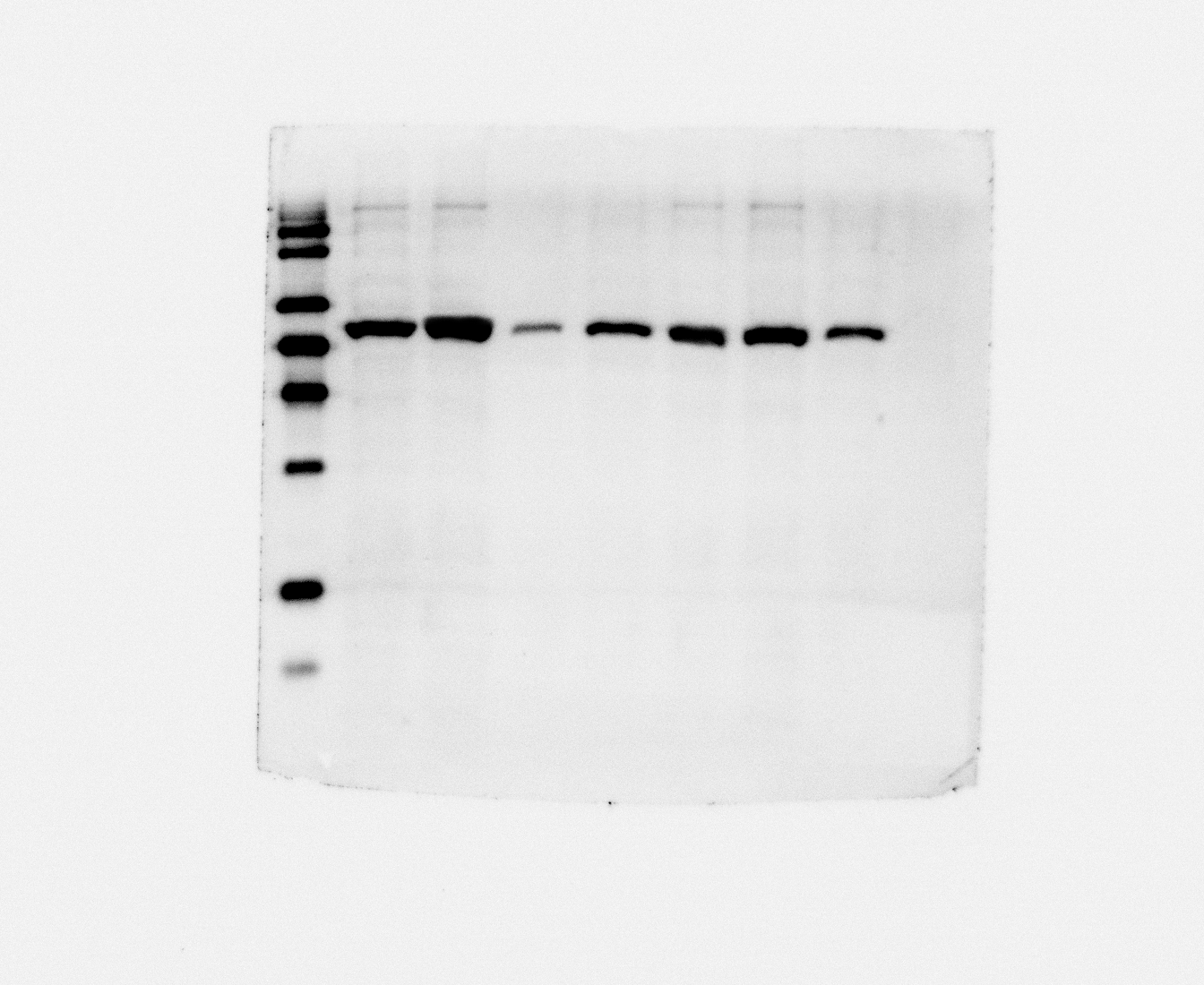

Supplement: Supplementary file 1 [file biomolecules-15-01188-s001.zip › File S1. Original Images for Blots/Figure3 C/2-METTL3-2.tif]

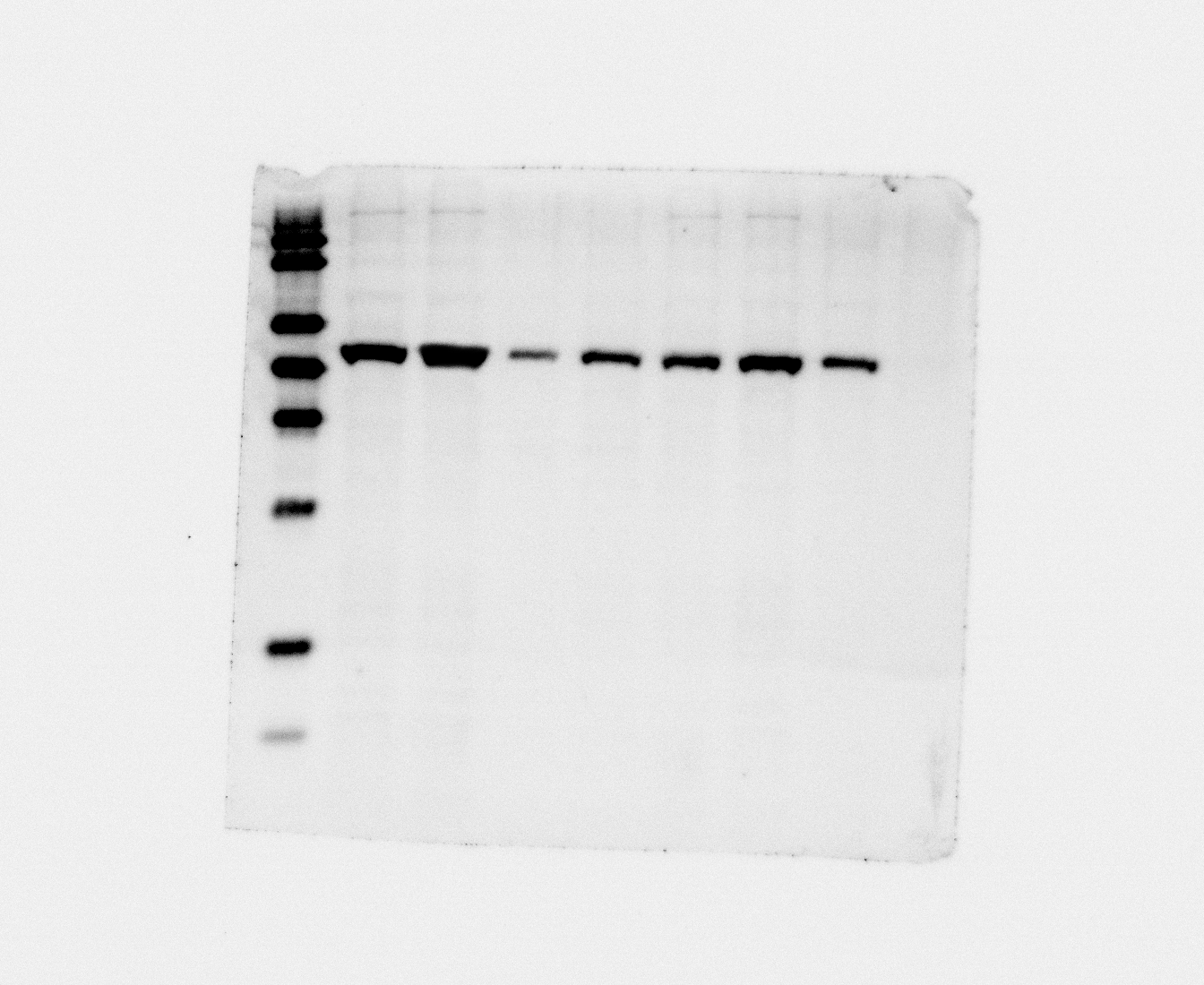

Supplement: Supplementary file 1 [file biomolecules-15-01188-s001.zip › File S1. Original Images for Blots/Figure3 C/2-METTL3-3.tif]

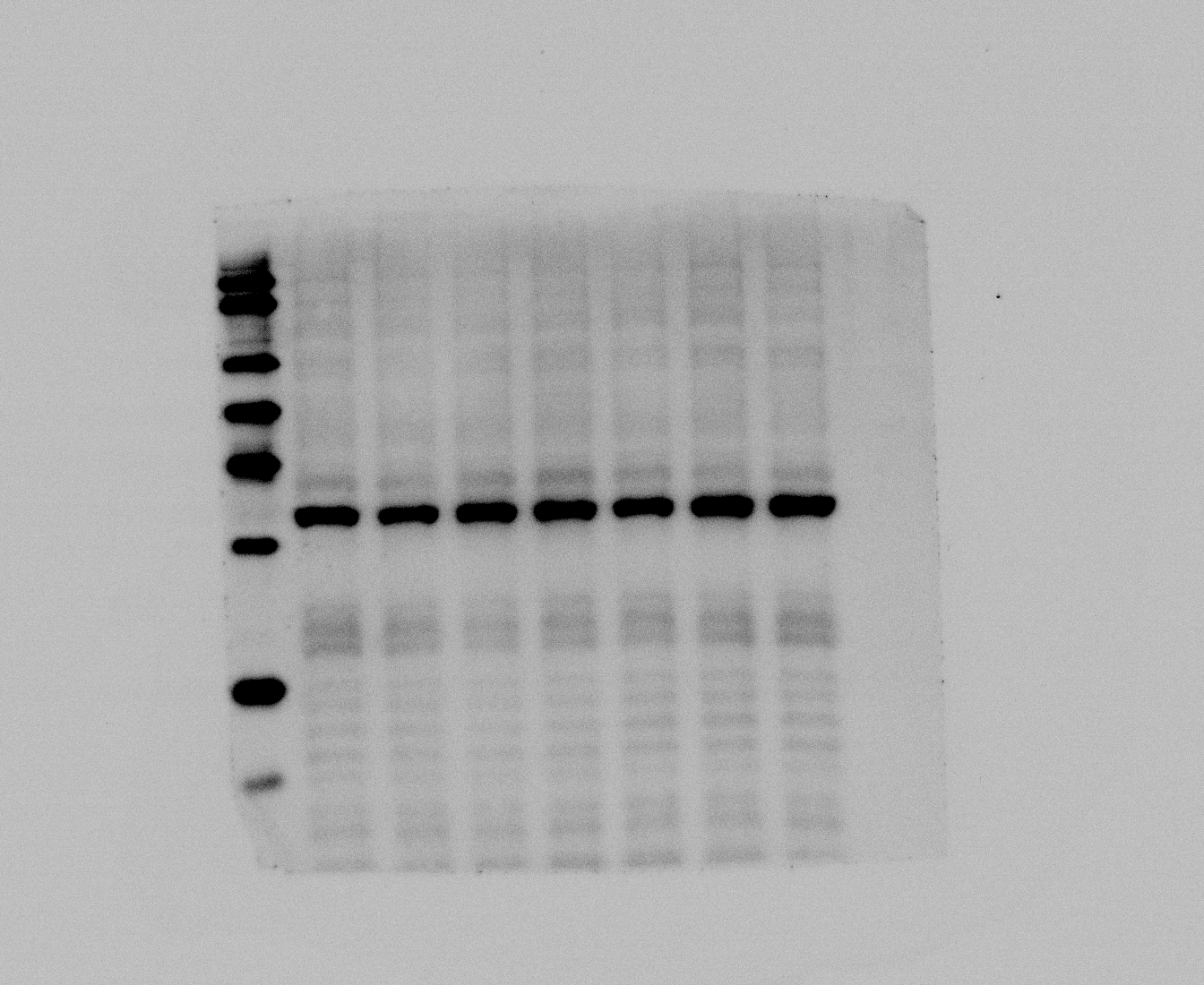

Supplement: Supplementary file 1 [file biomolecules-15-01188-s001.zip › File S1. Original Images for Blots/Figure3 C/2-β-actin-1 报告.tif]

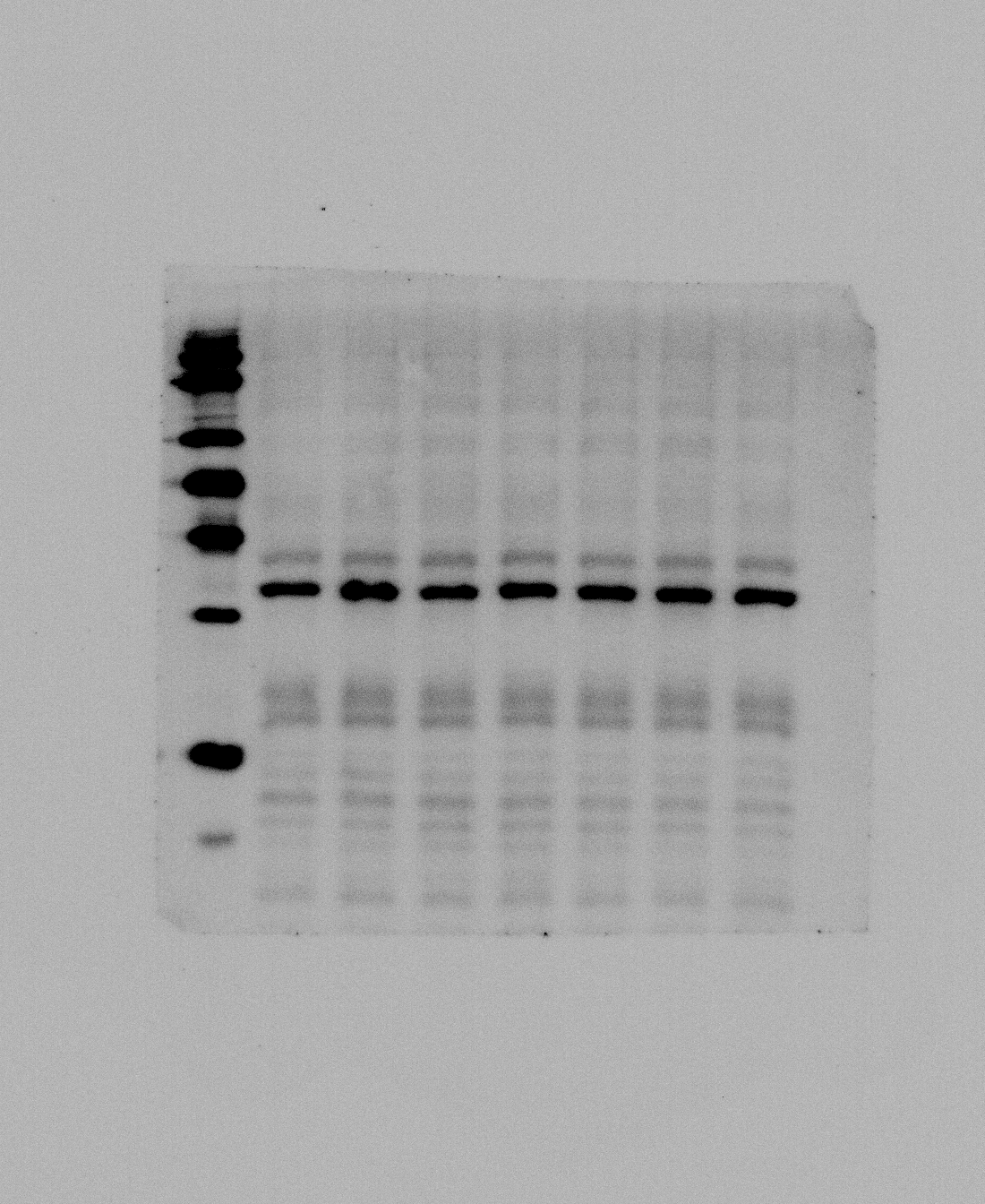

Supplement: Supplementary file 1 [file biomolecules-15-01188-s001.zip › File S1. Original Images for Blots/Figure3 C/2-β-actin-2.tif]

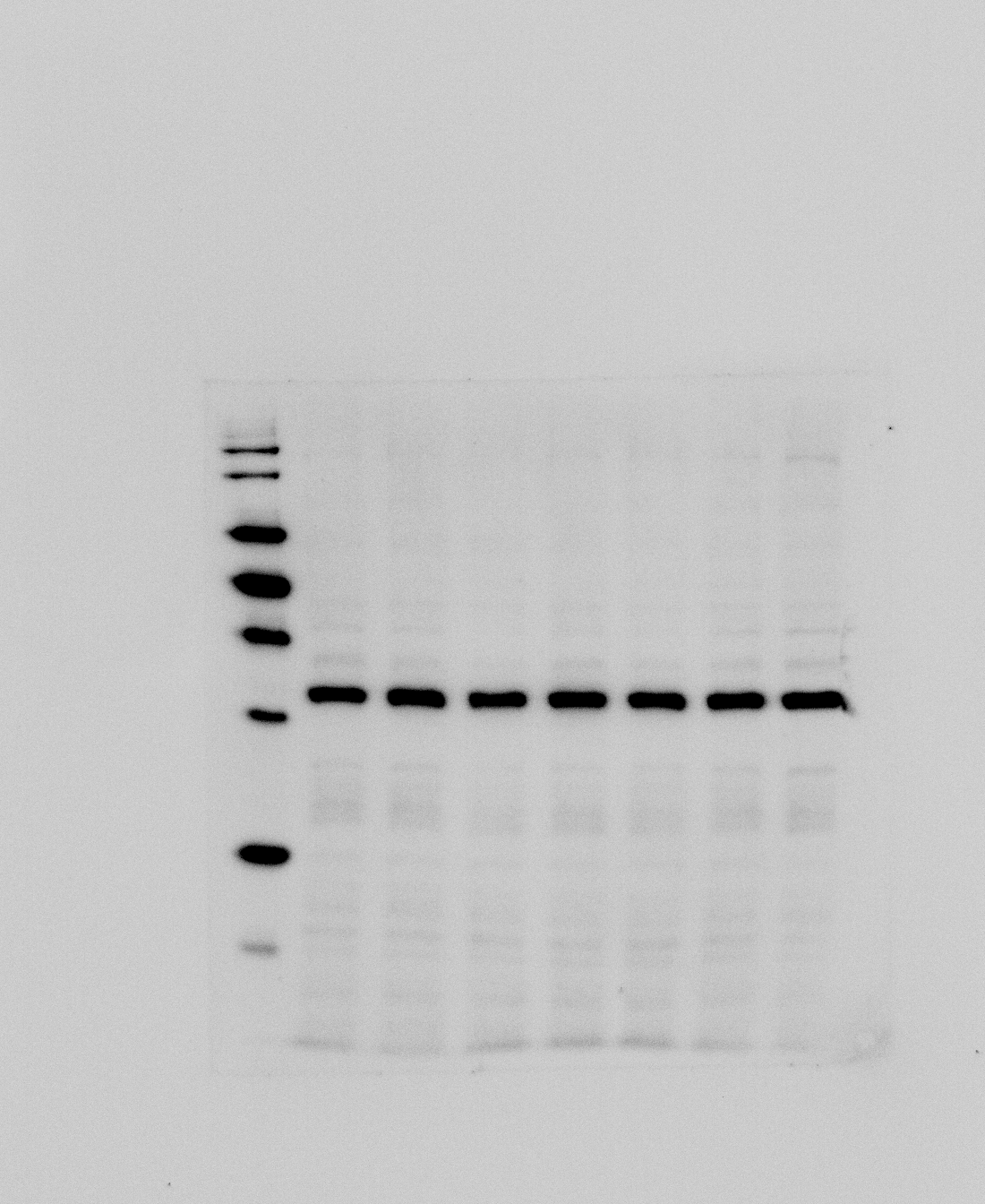

Supplement: Supplementary file 1 [file biomolecules-15-01188-s001.zip › File S1. Original Images for Blots/Figure3 C/2-β-actin-3.tif]

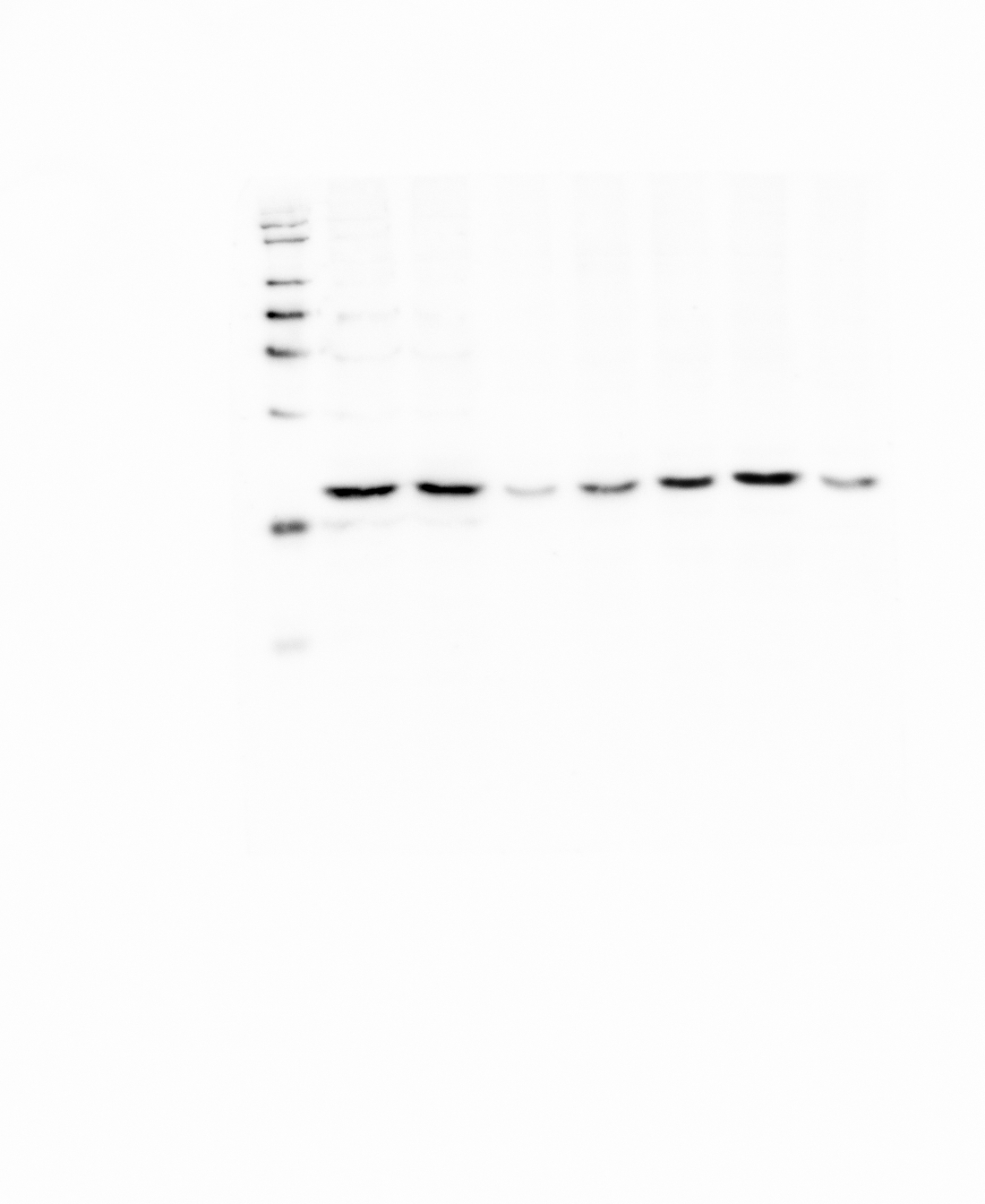

Supplement: Supplementary file 1 [file biomolecules-15-01188-s001.zip › File S1. Original Images for Blots/Figure3 D/3-BDNF-1.tif]

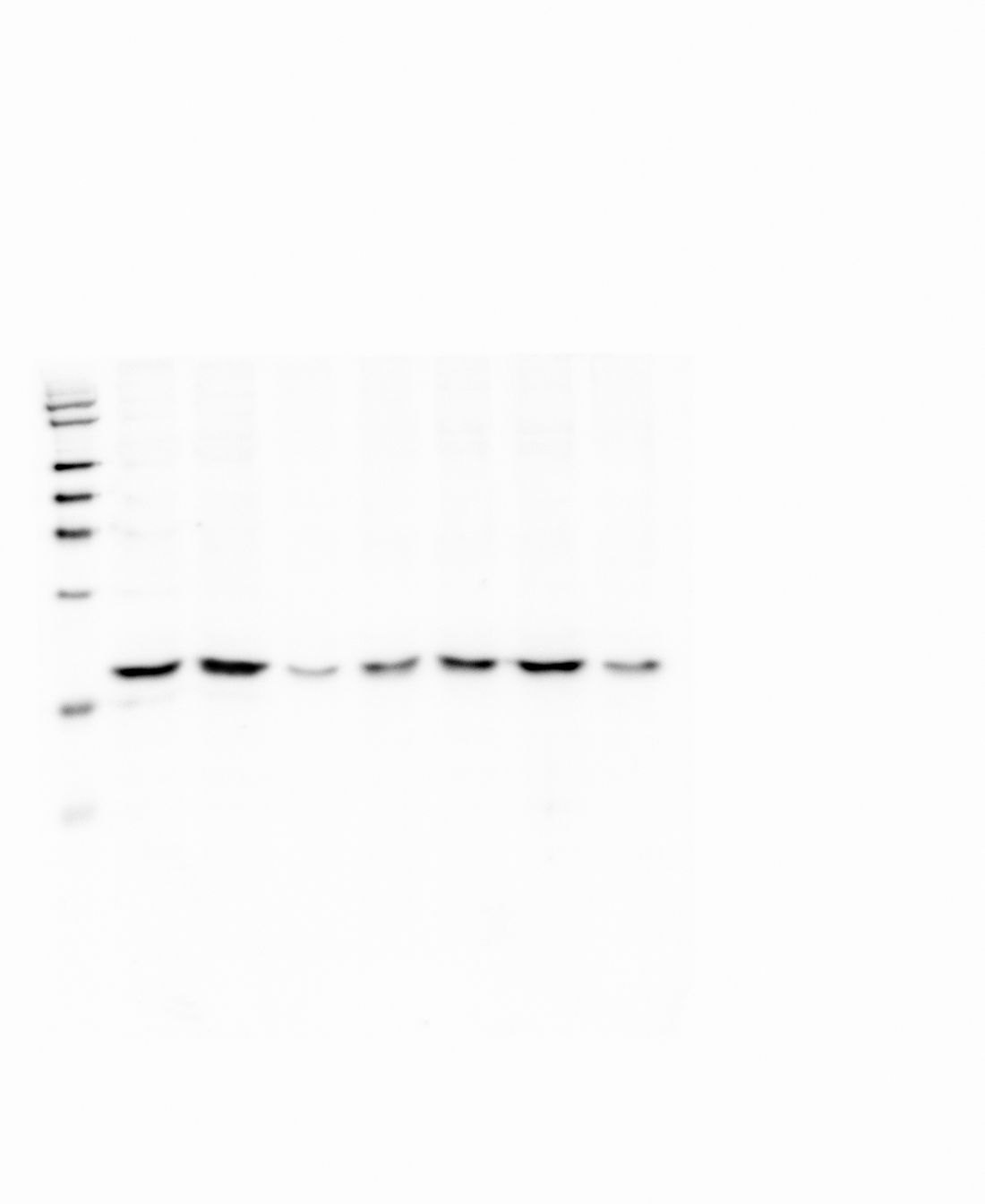

Supplement: Supplementary file 1 [file biomolecules-15-01188-s001.zip › File S1. Original Images for Blots/Figure3 D/3-BDNF-2 报告.tif]

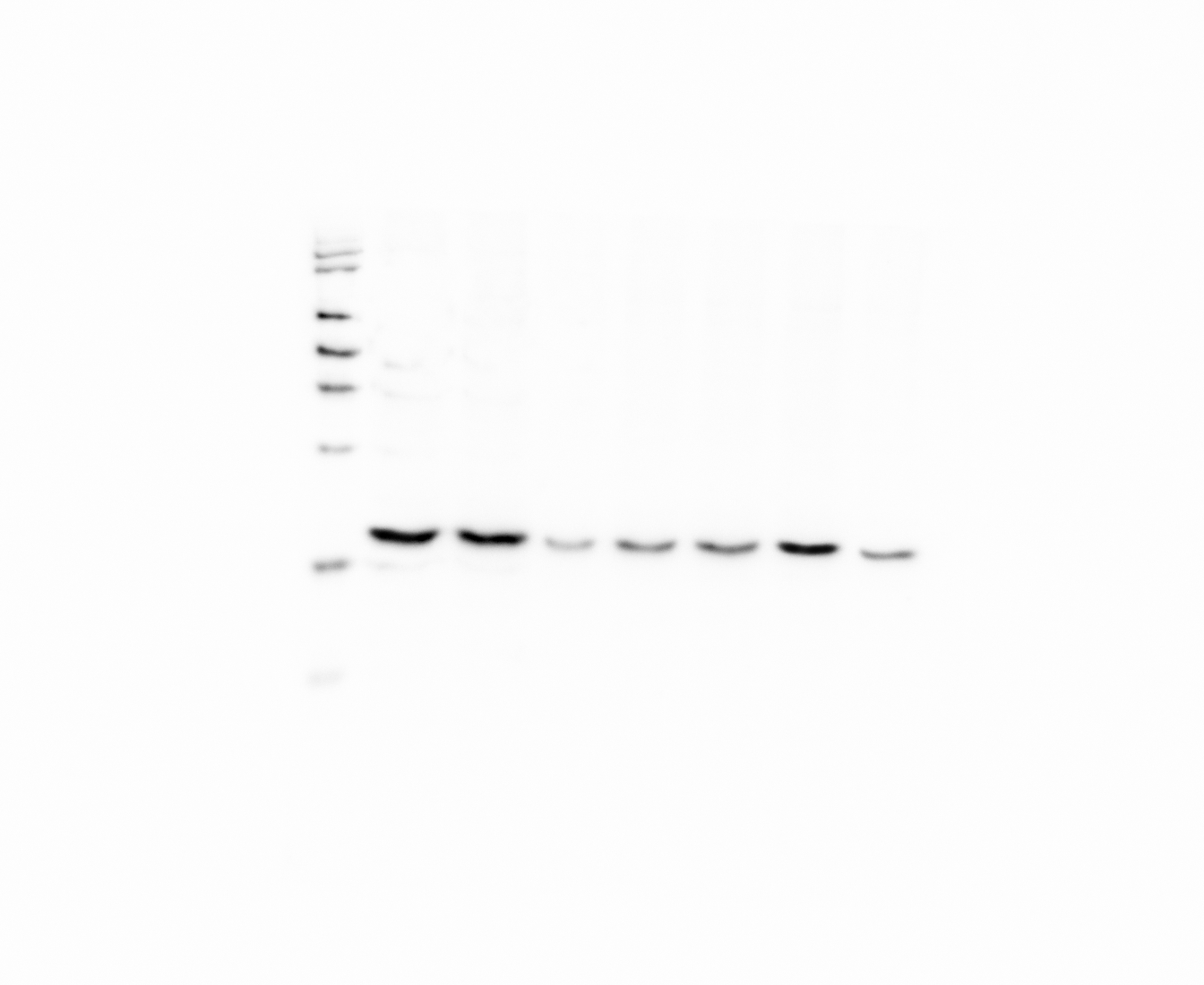

Supplement: Supplementary file 1 [file biomolecules-15-01188-s001.zip › File S1. Original Images for Blots/Figure3 D/3-BDNF-3.tif]

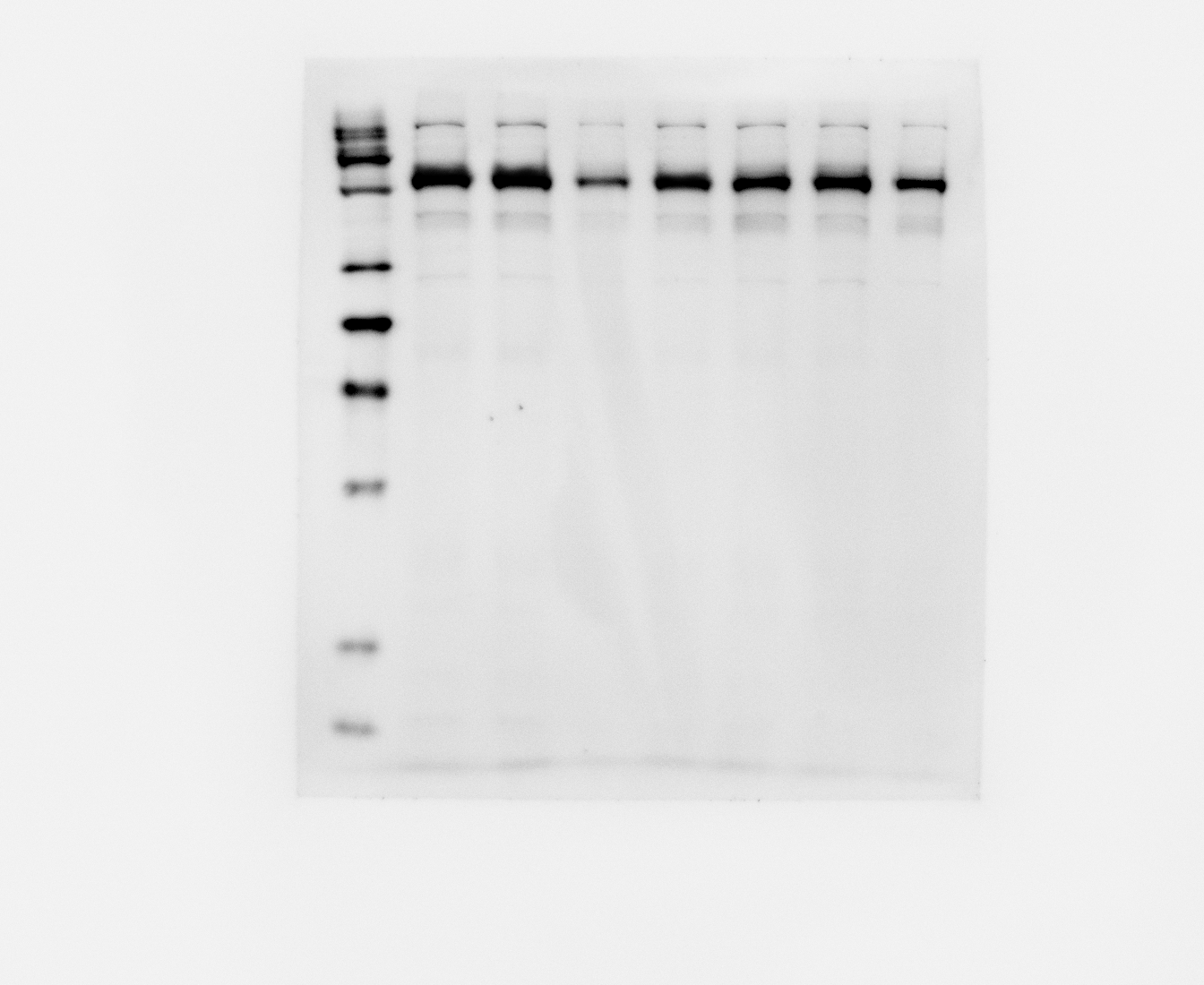

Supplement: Supplementary file 1 [file biomolecules-15-01188-s001.zip › File S1. Original Images for Blots/Figure3 D/3-p-TrkB-1.tif]

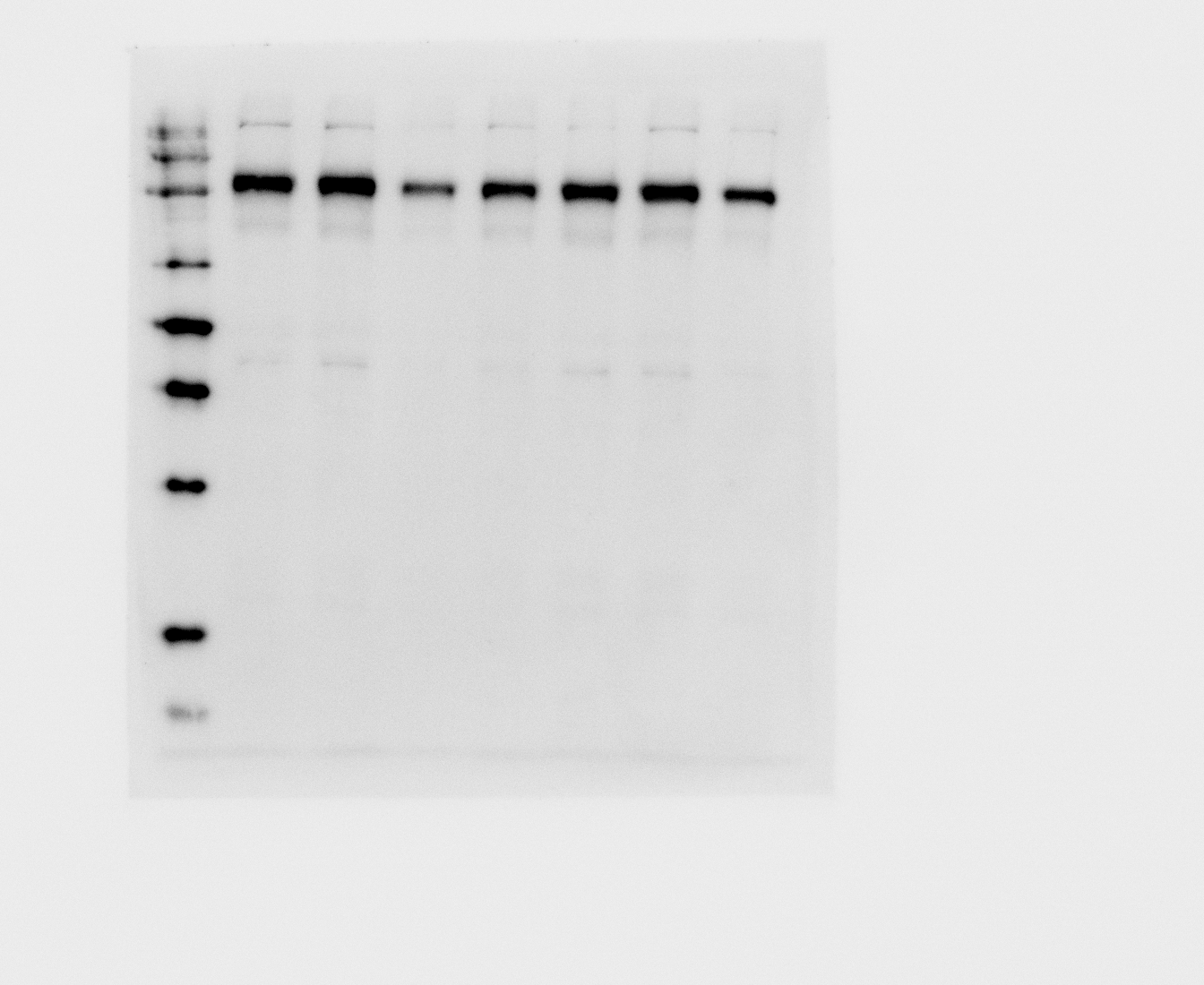

Supplement: Supplementary file 1 [file biomolecules-15-01188-s001.zip › File S1. Original Images for Blots/Figure3 D/3-p-TrkB-2 报告.tif]

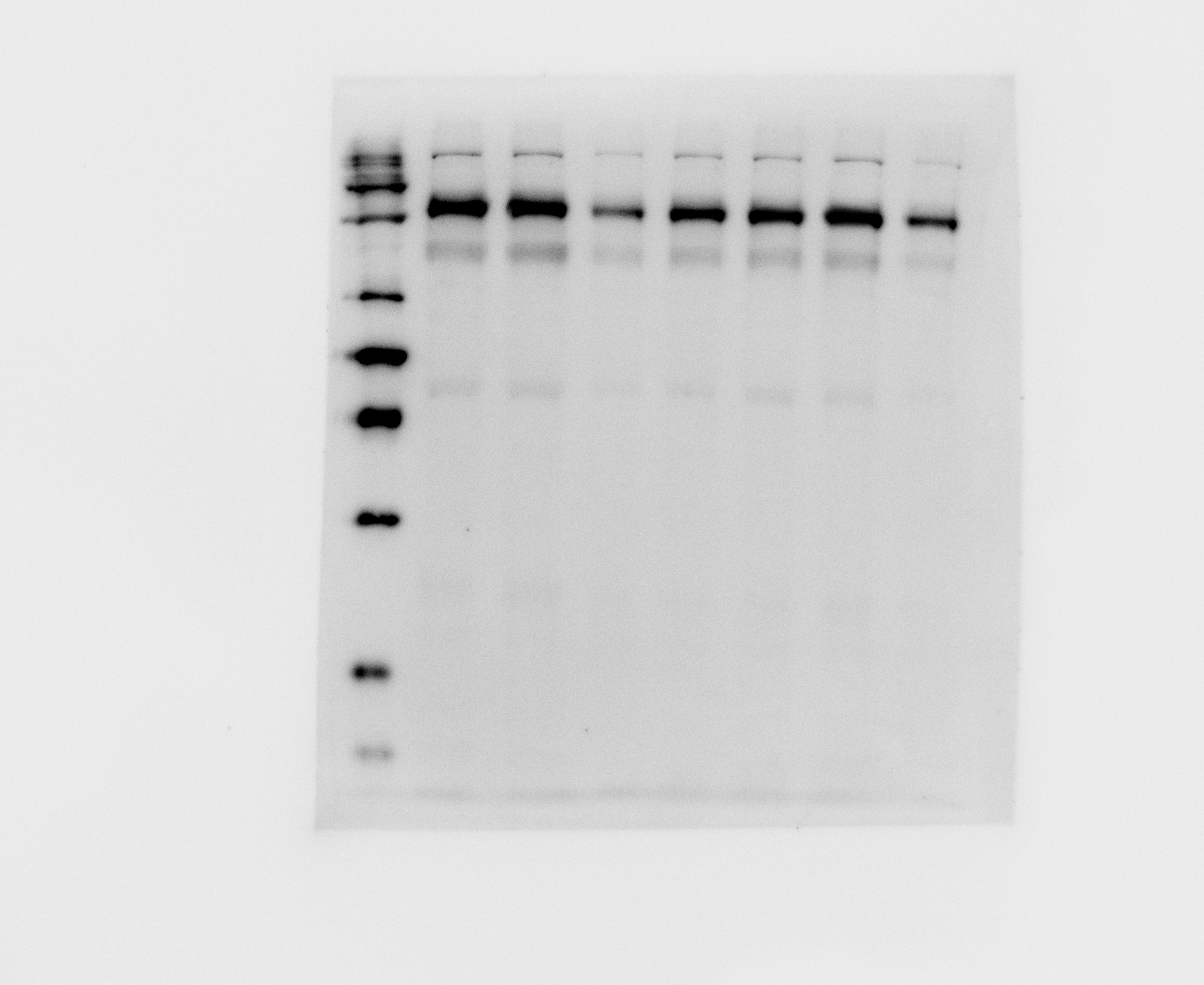

Supplement: Supplementary file 1 [file biomolecules-15-01188-s001.zip › File S1. Original Images for Blots/Figure3 D/3-p-TrkB-3.tif]

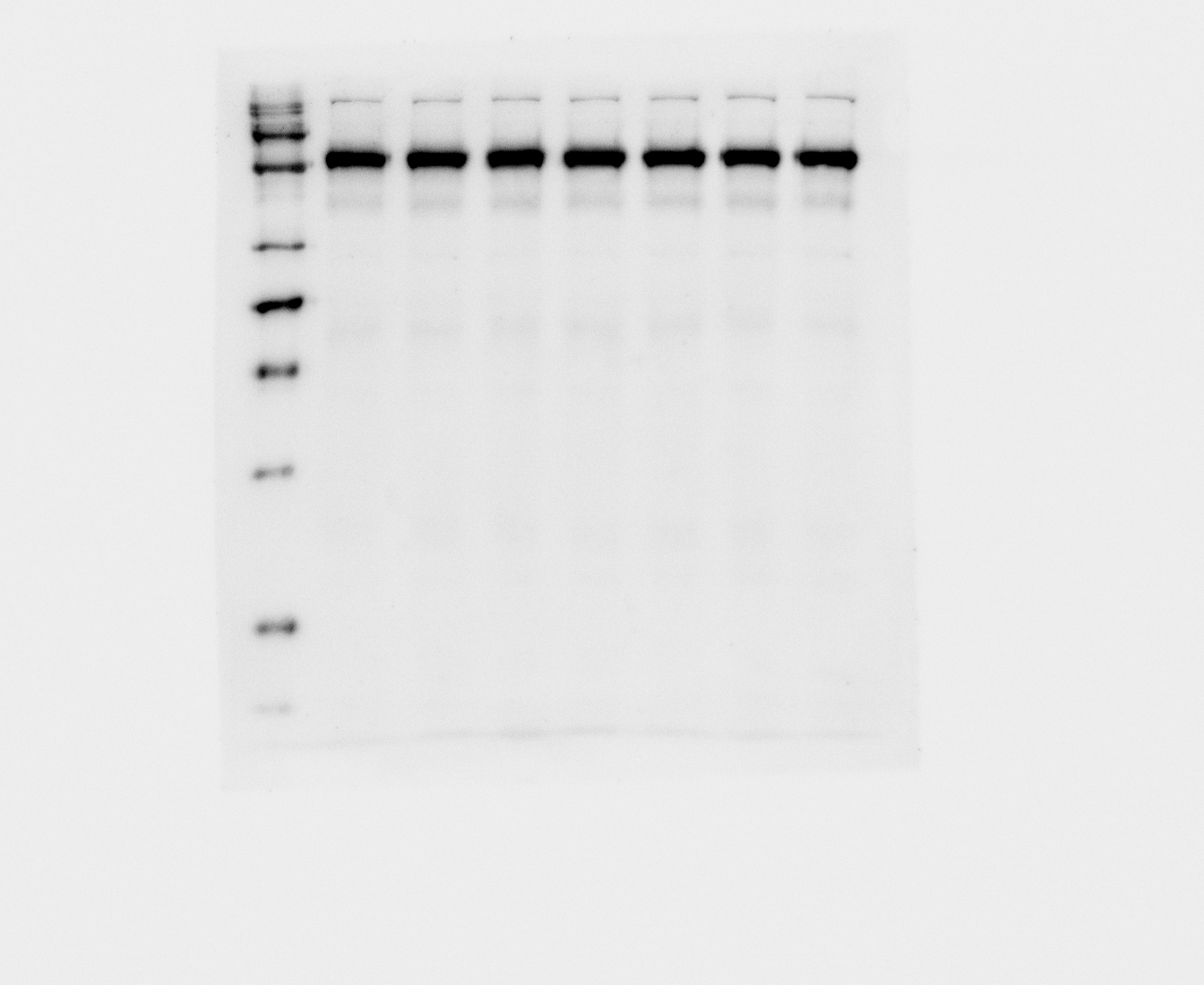

Supplement: Supplementary file 1 [file biomolecules-15-01188-s001.zip › File S1. Original Images for Blots/Figure3 D/3-TrkB-1 报告.tif]

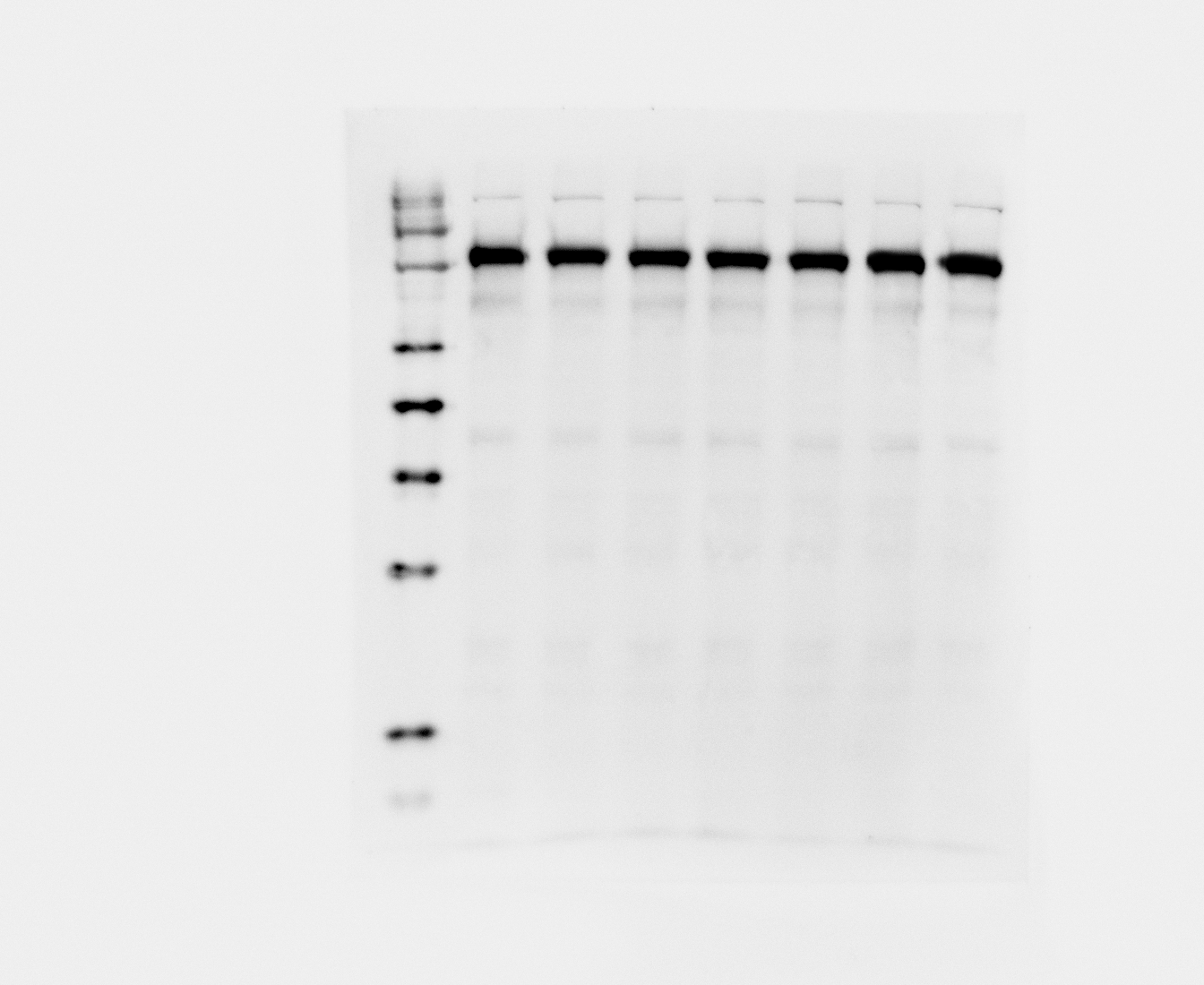

Supplement: Supplementary file 1 [file biomolecules-15-01188-s001.zip › File S1. Original Images for Blots/Figure3 D/3-TrkB-2.tif]

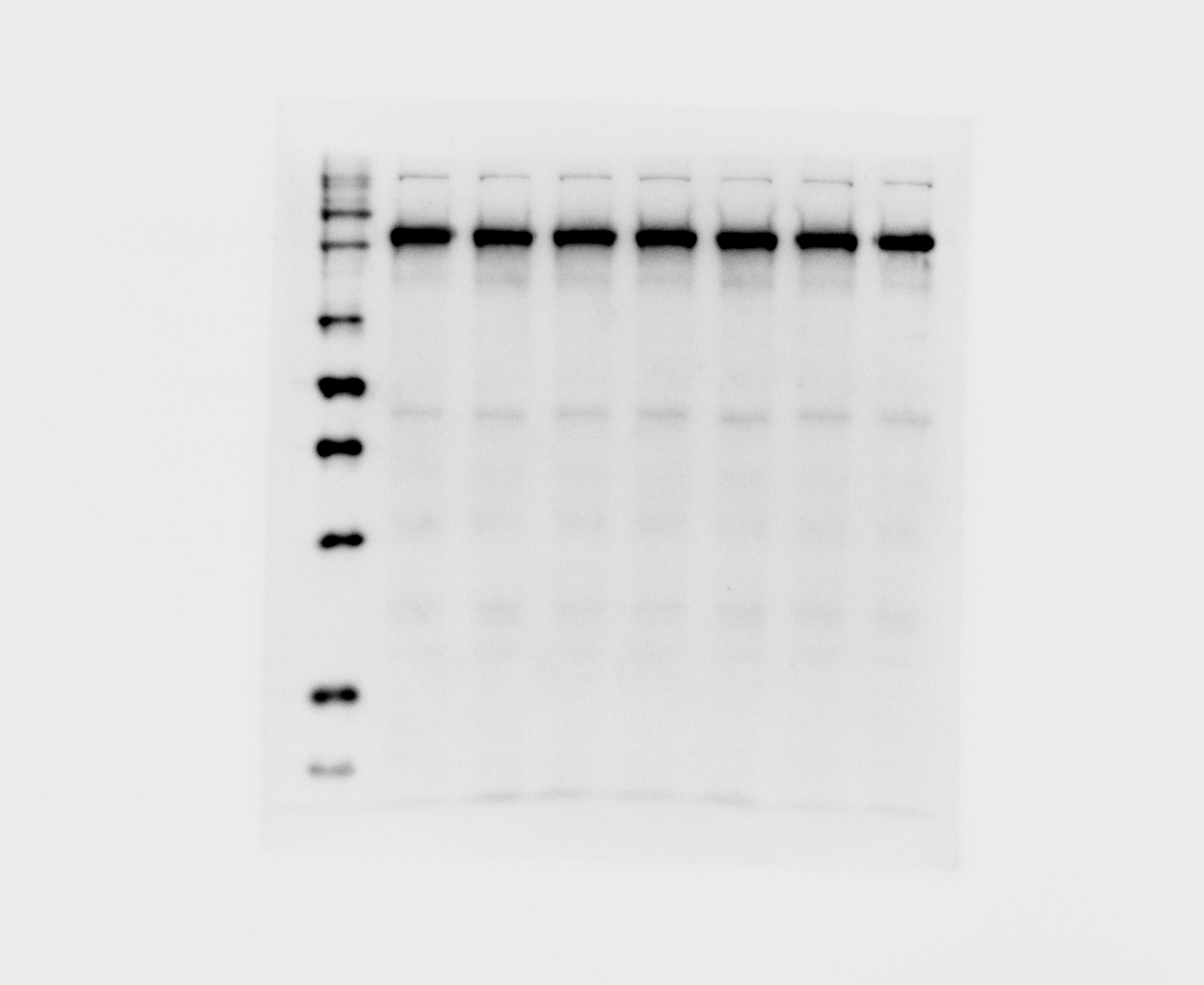

Supplement: Supplementary file 1 [file biomolecules-15-01188-s001.zip › File S1. Original Images for Blots/Figure3 D/3-TrkB-3.tif]

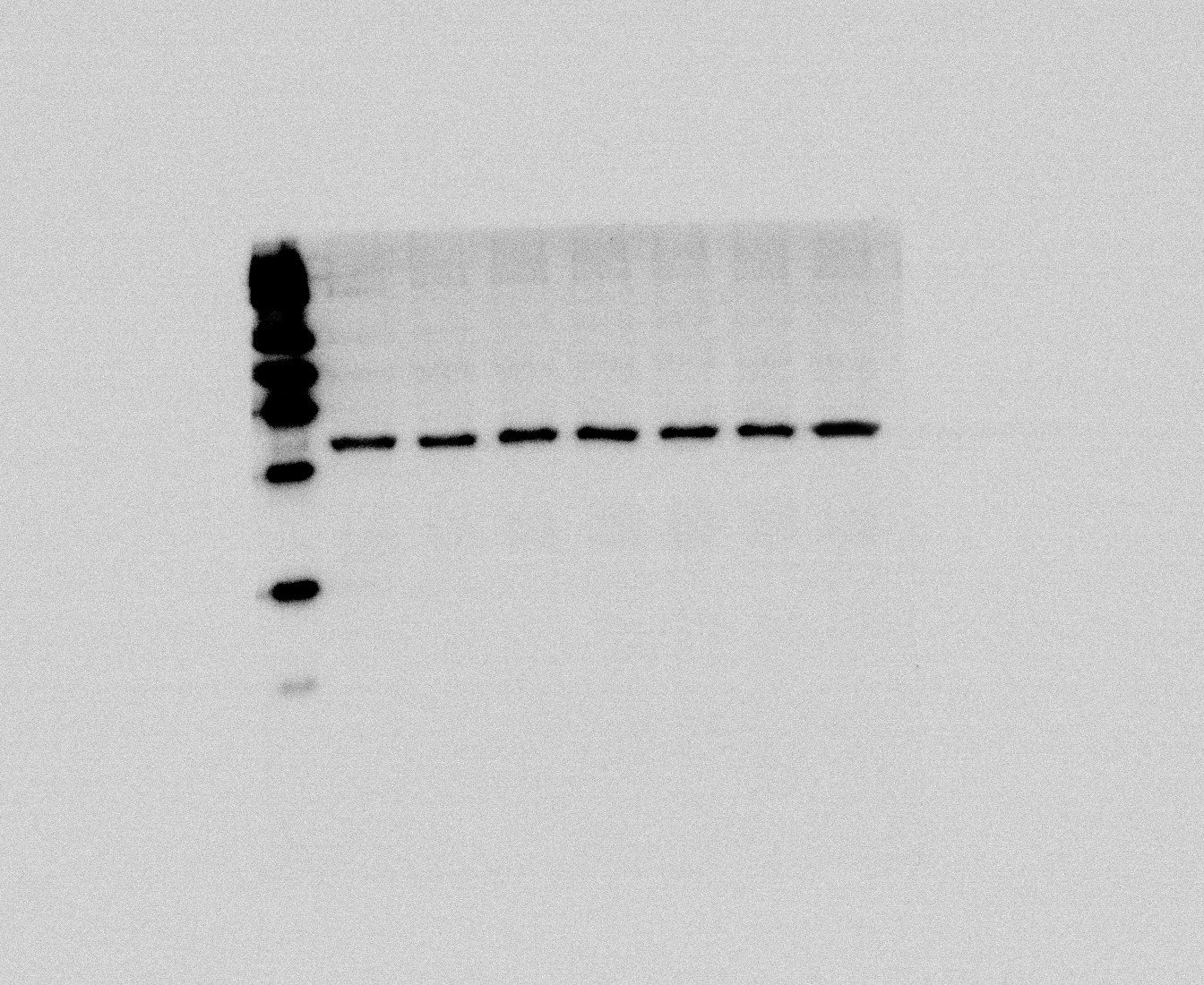

Supplement: Supplementary file 1 [file biomolecules-15-01188-s001.zip › File S1. Original Images for Blots/Figure3 D/3-β-actin-1 报告.tif]

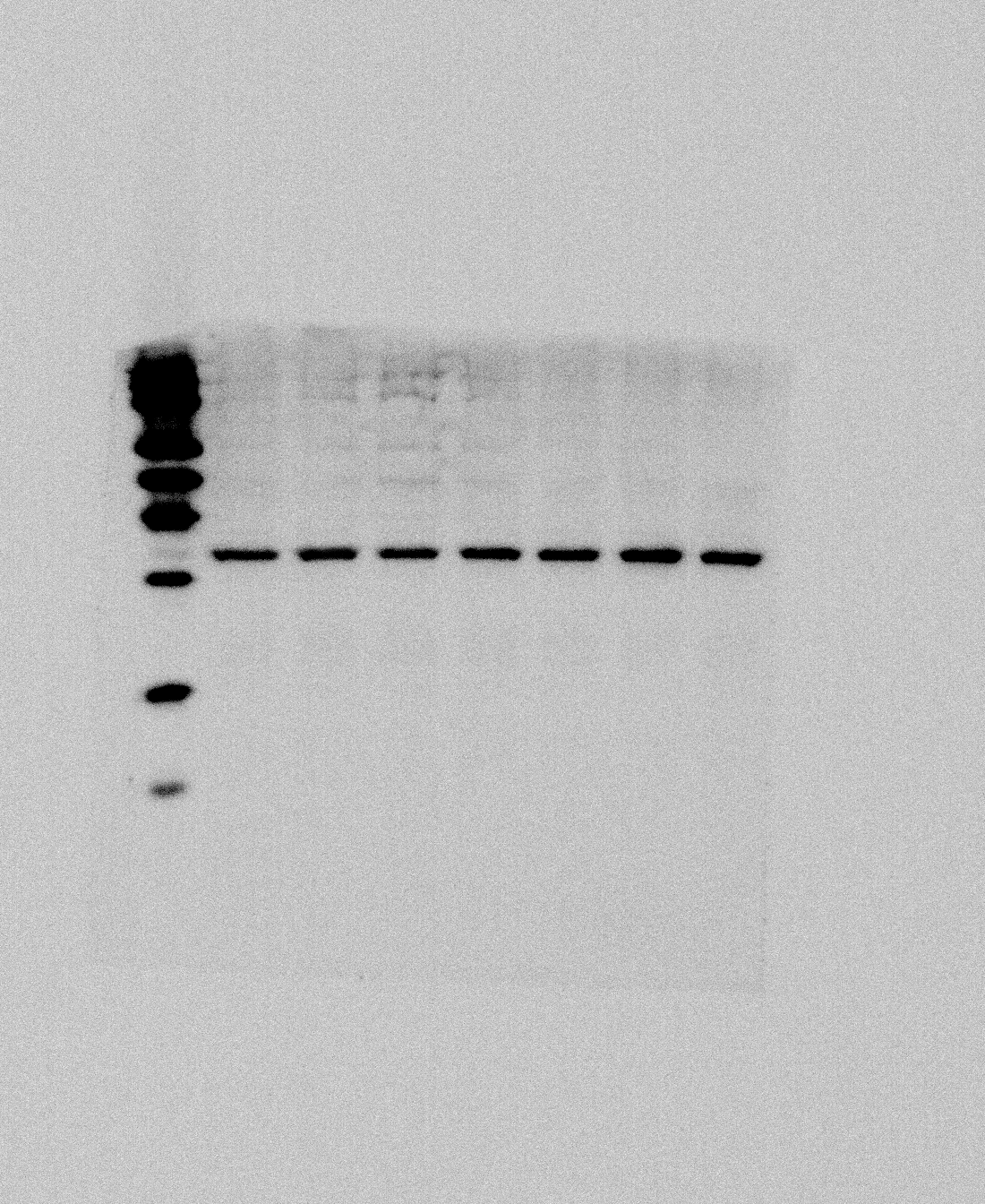

Supplement: Supplementary file 1 [file biomolecules-15-01188-s001.zip › File S1. Original Images for Blots/Figure3 D/3-β-actin-2.tif]

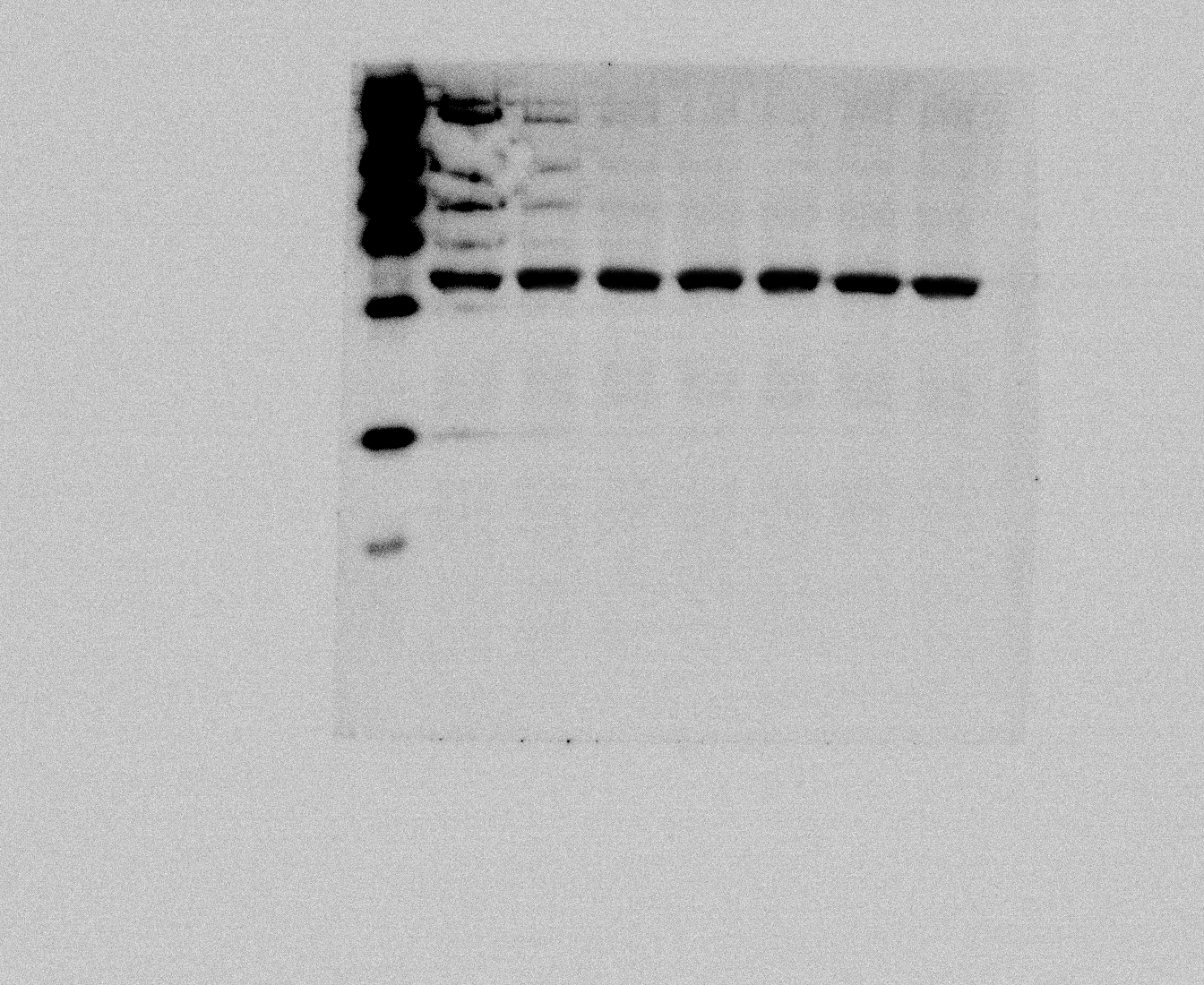

Supplement: Supplementary file 1 [file biomolecules-15-01188-s001.zip › File S1. Original Images for Blots/Figure3 D/3-β-actin-3.tif]

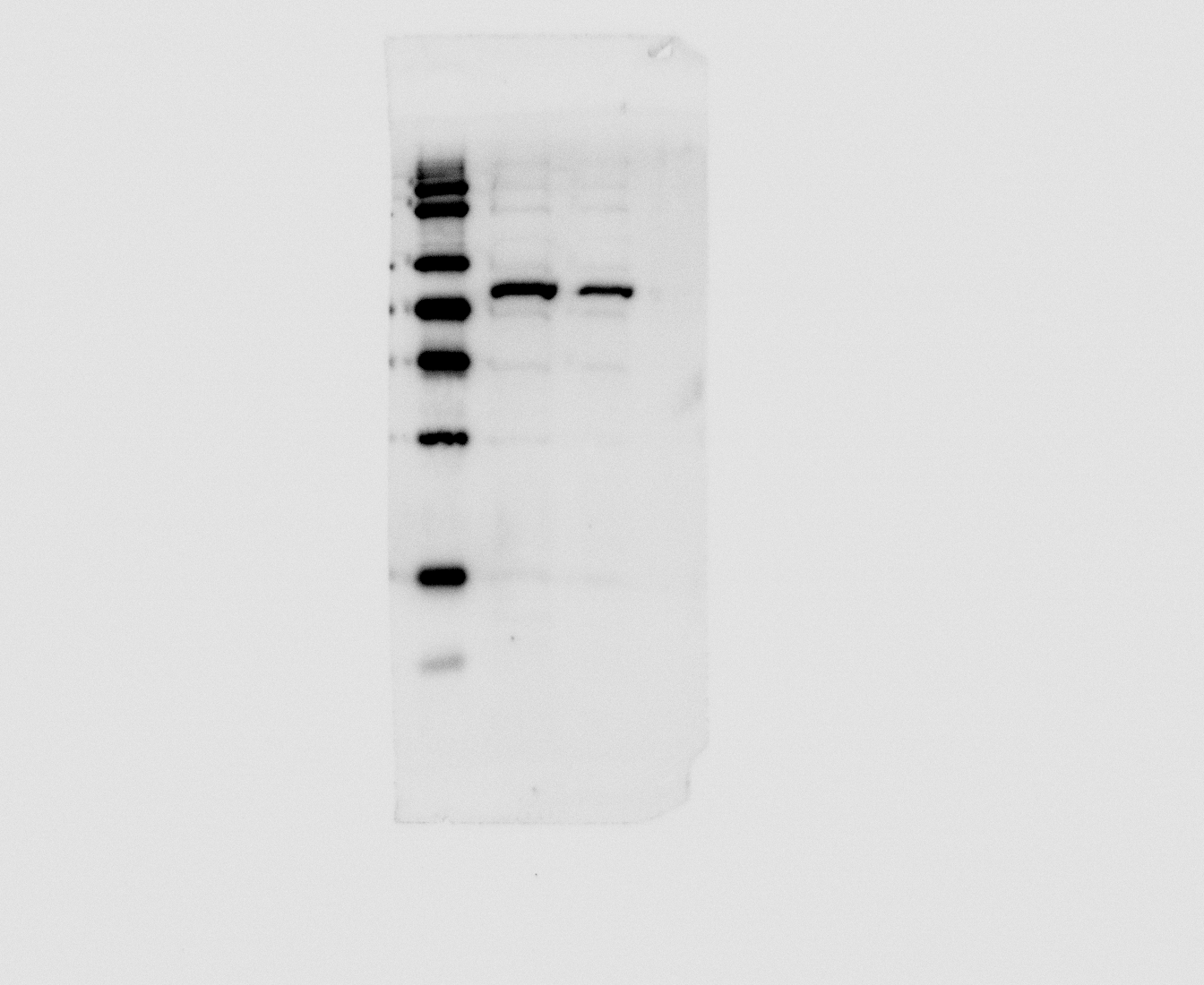

Supplement: Supplementary file 1 [file biomolecules-15-01188-s001.zip › File S1. Original Images for Blots/Figure4 B/4-METTL3-1 报告.tif]

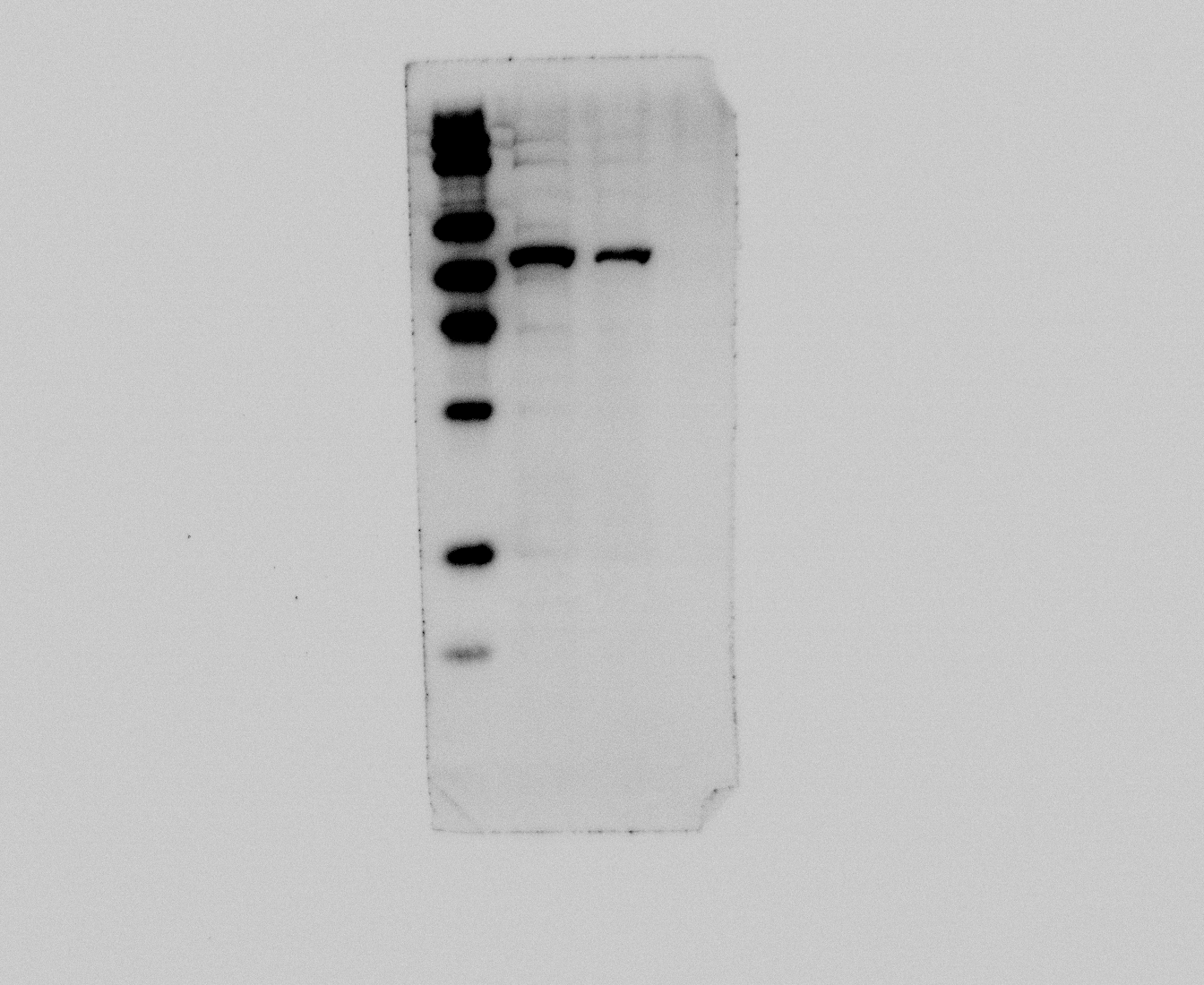

Supplement: Supplementary file 1 [file biomolecules-15-01188-s001.zip › File S1. Original Images for Blots/Figure4 B/4-METTL3-2.tif]

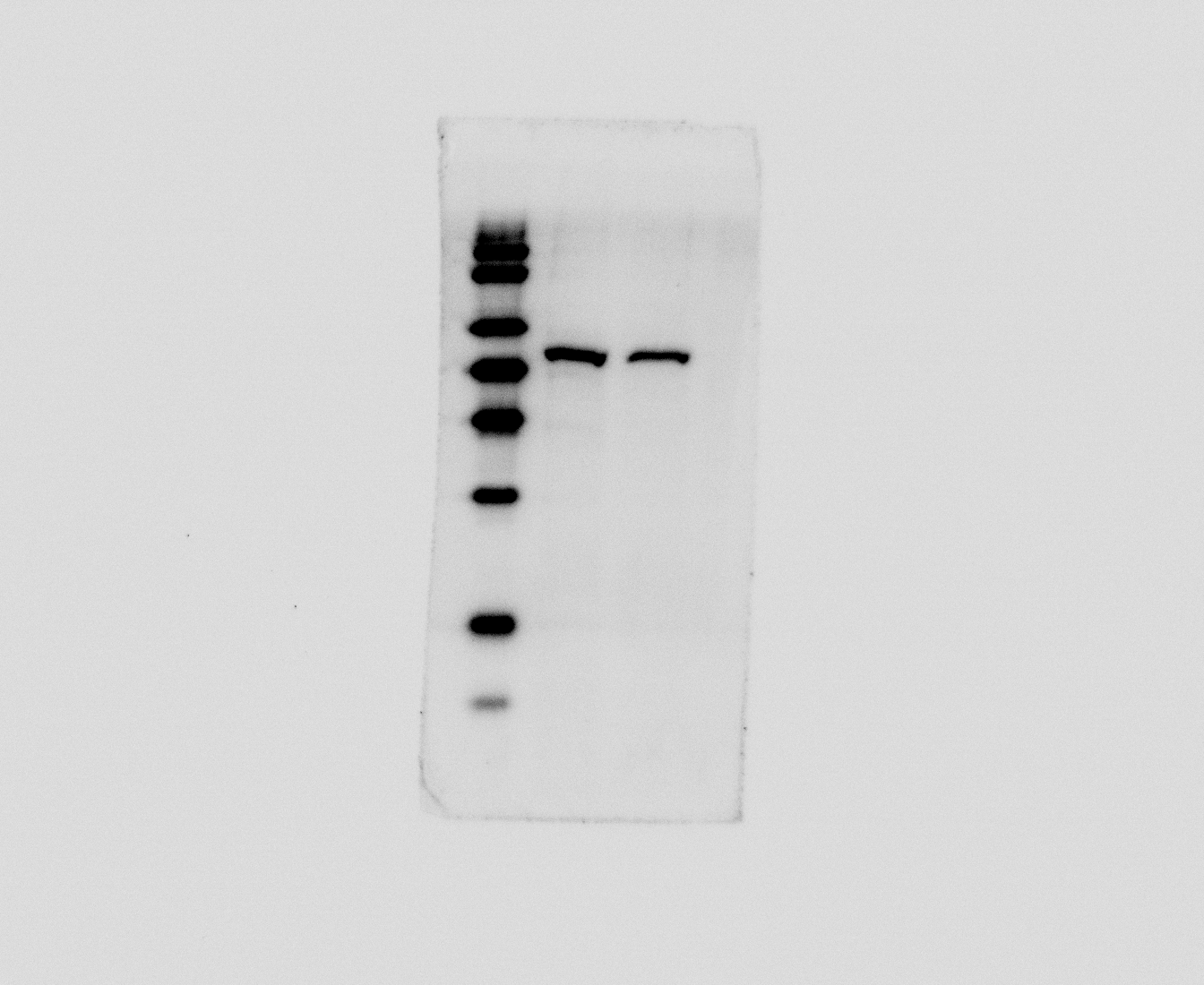

Supplement: Supplementary file 1 [file biomolecules-15-01188-s001.zip › File S1. Original Images for Blots/Figure4 B/4-METTL3-3.tif]

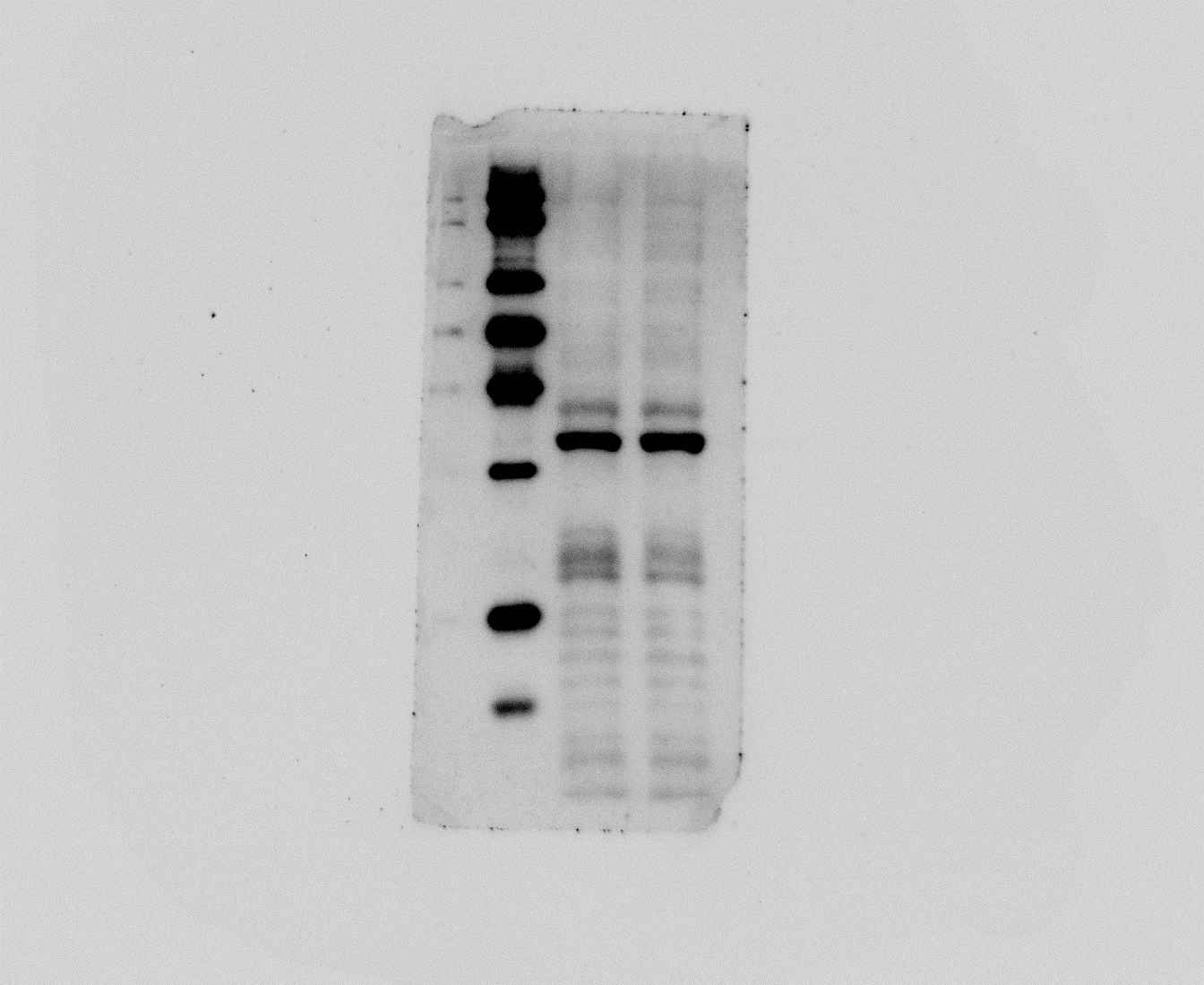

Supplement: Supplementary file 1 [file biomolecules-15-01188-s001.zip › File S1. Original Images for Blots/Figure4 B/4-β-actin-1.tif]

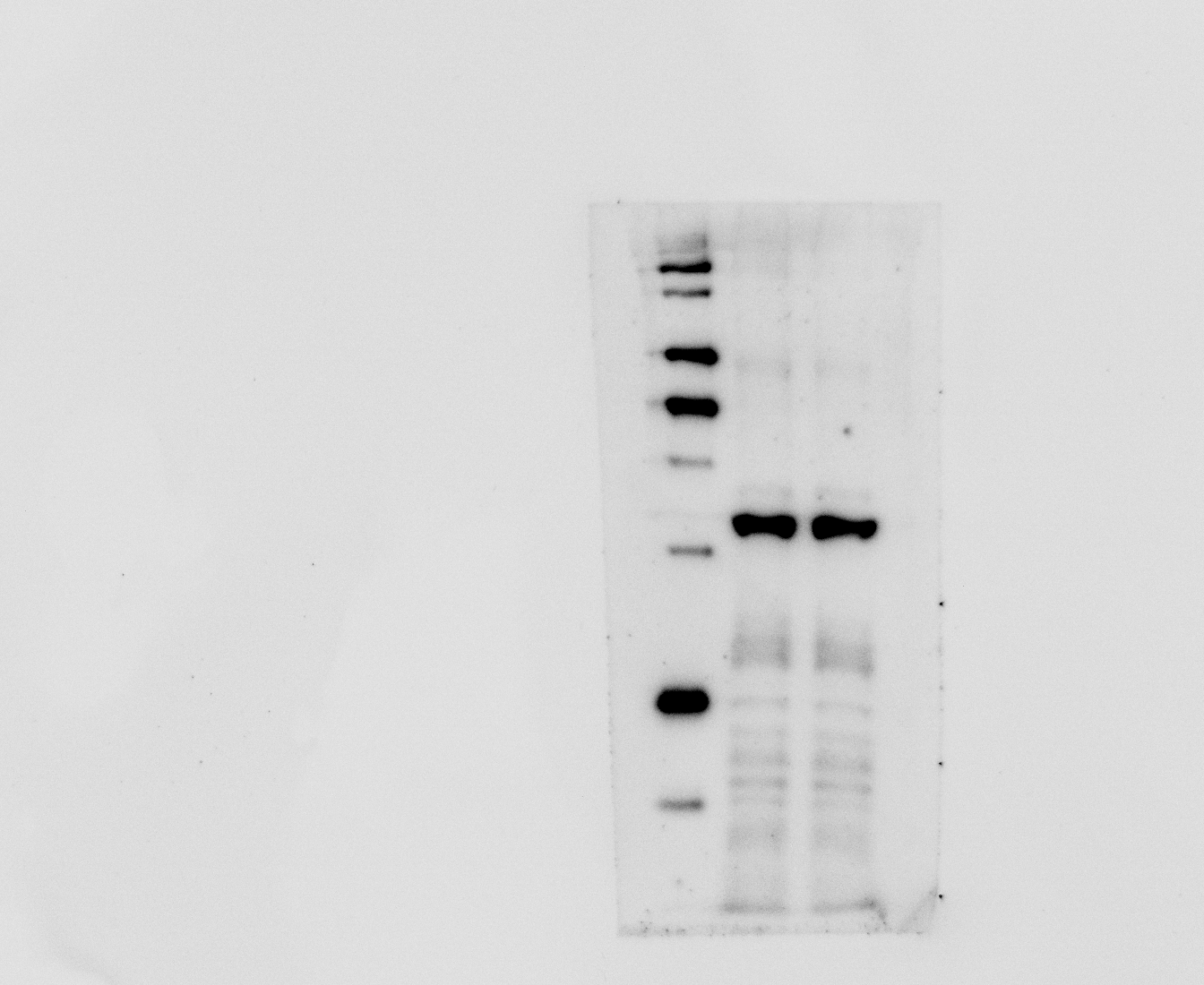

Supplement: Supplementary file 1 [file biomolecules-15-01188-s001.zip › File S1. Original Images for Blots/Figure4 B/4-β-actin-2.tif]

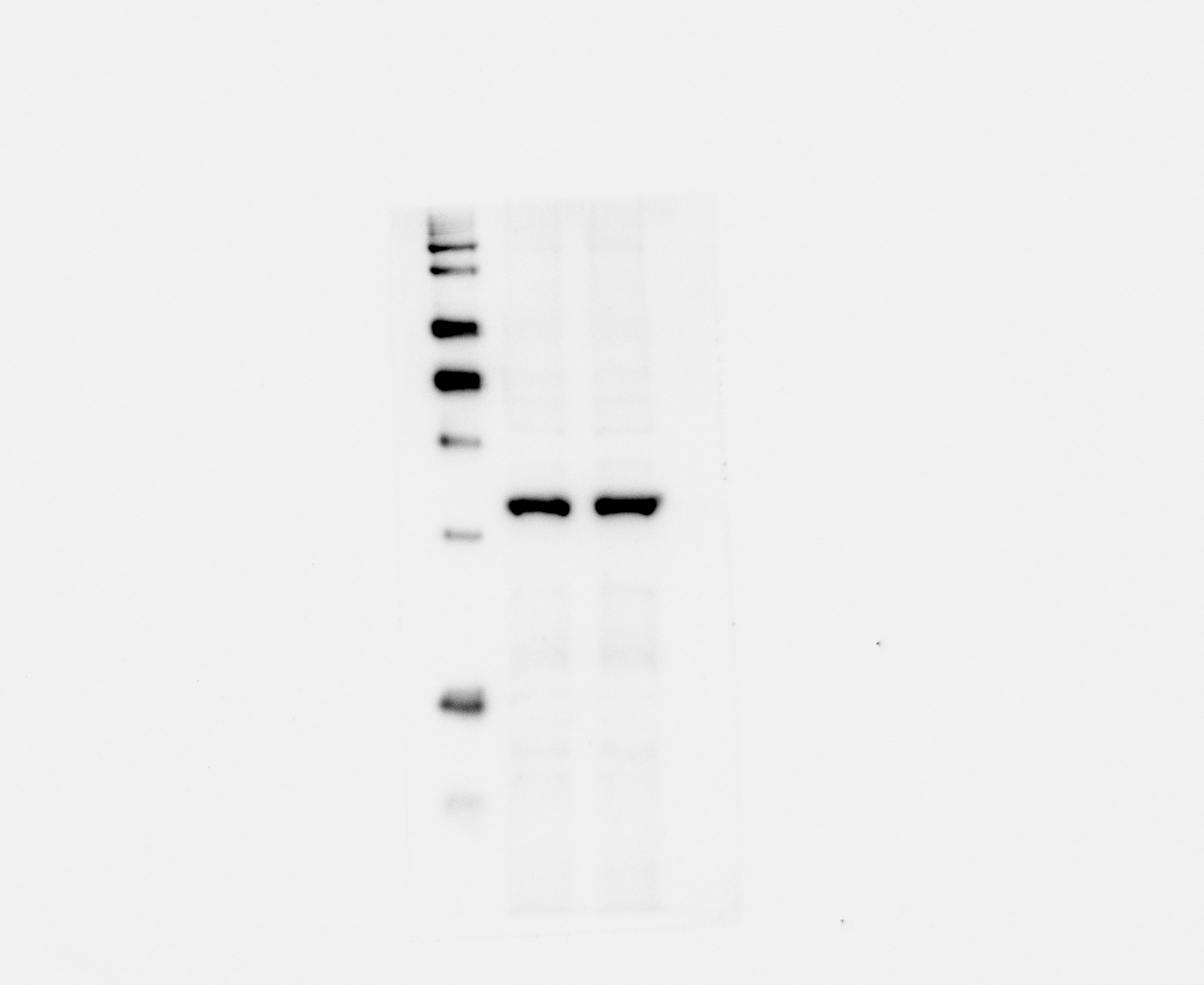

Supplement: Supplementary file 1 [file biomolecules-15-01188-s001.zip › File S1. Original Images for Blots/Figure4 B/4-β-actin-3 报告.tif]

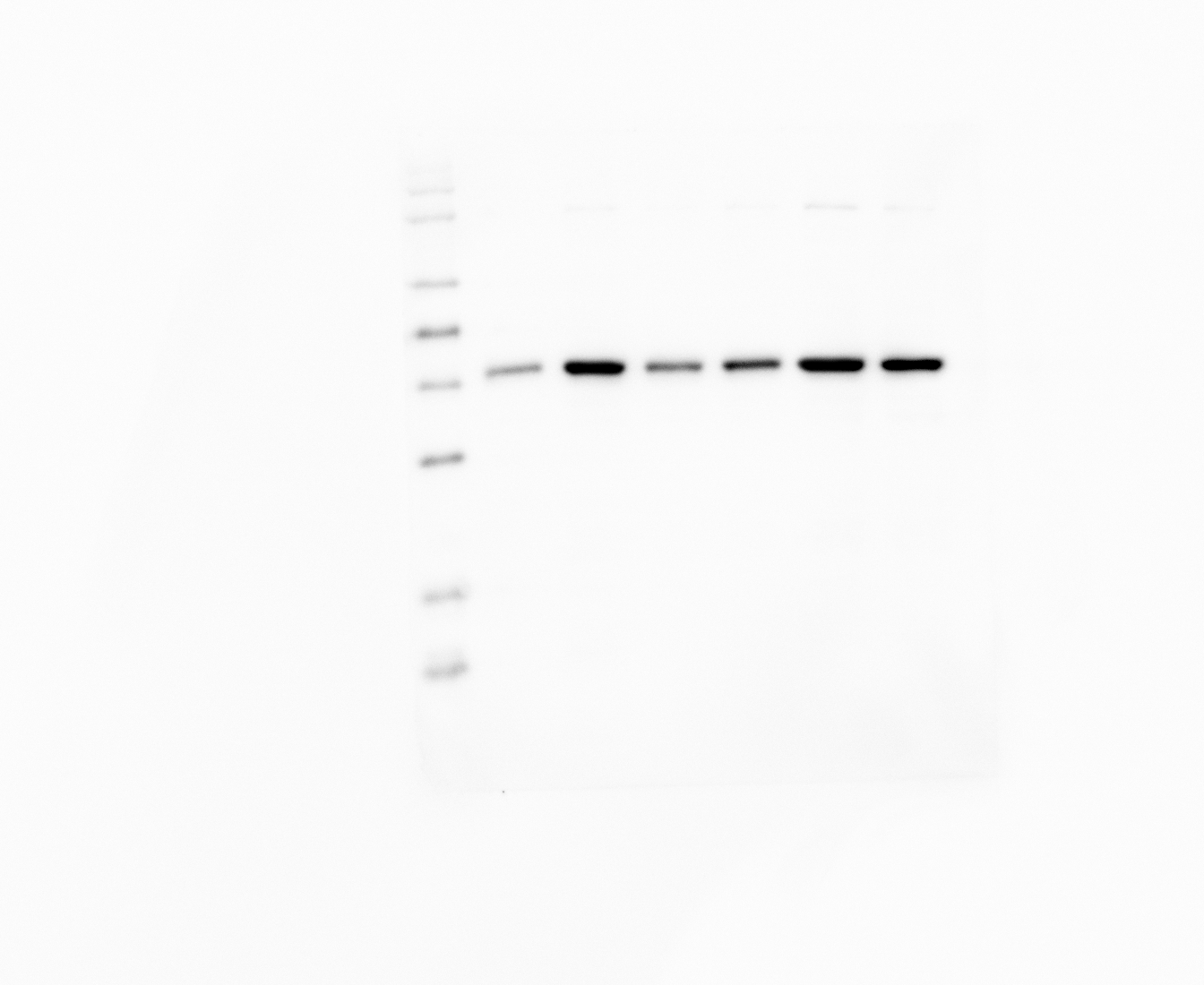

Supplement: Supplementary file 1 [file biomolecules-15-01188-s001.zip › File S1. Original Images for Blots/Figure5 C/5-GFAP-1 报告.tif]

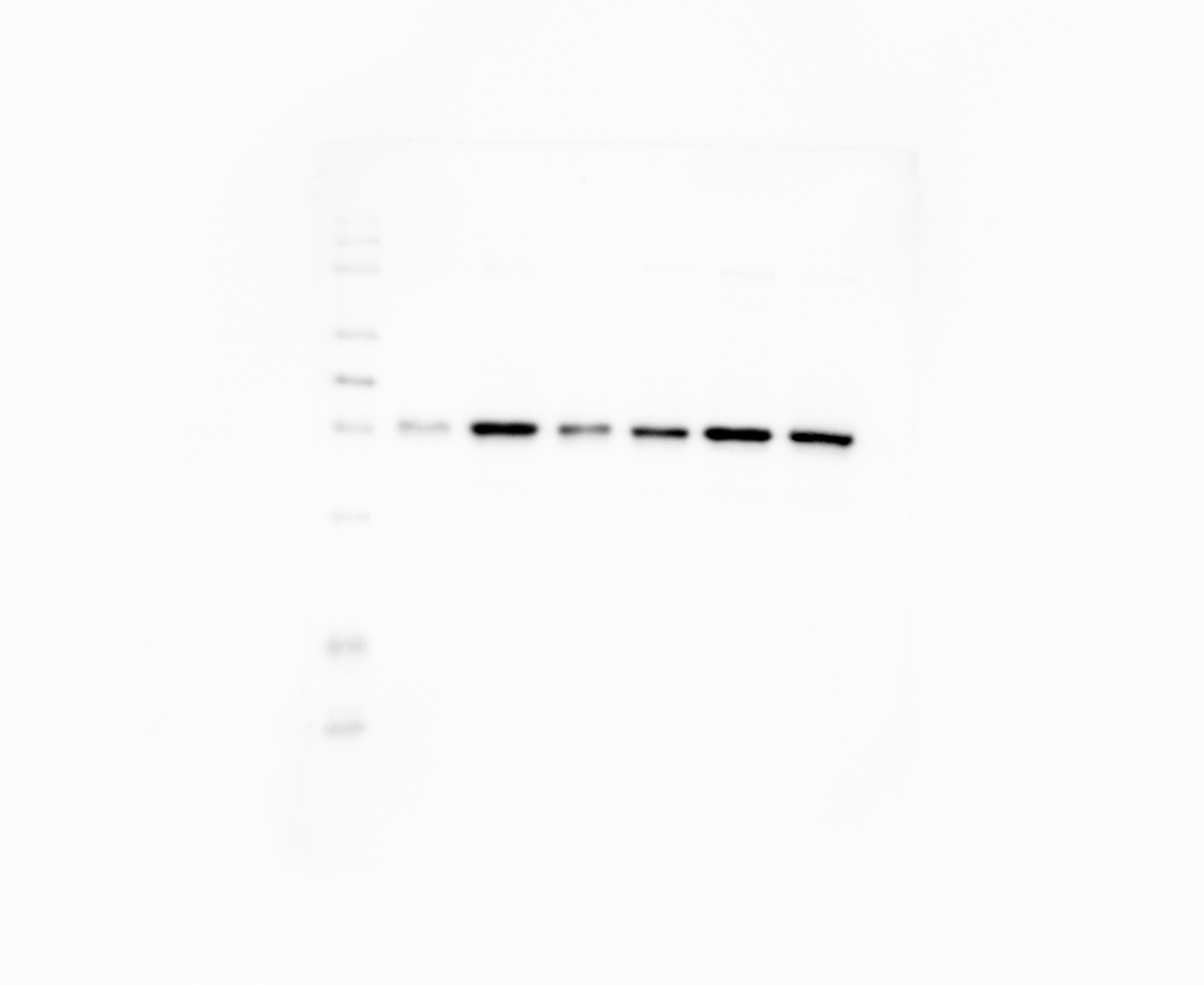

Supplement: Supplementary file 1 [file biomolecules-15-01188-s001.zip › File S1. Original Images for Blots/Figure5 C/5-GFAP-2.tif]

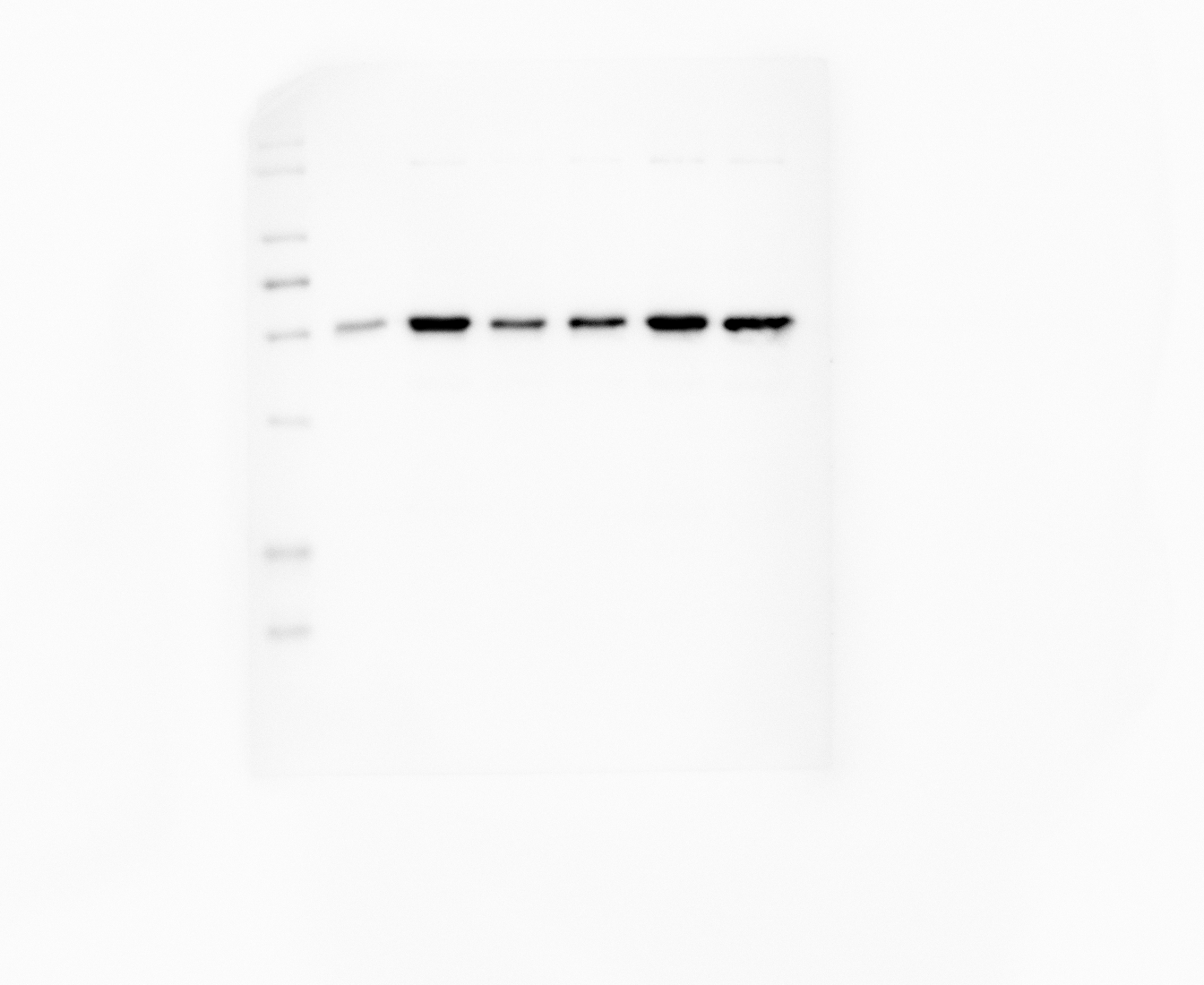

Supplement: Supplementary file 1 [file biomolecules-15-01188-s001.zip › File S1. Original Images for Blots/Figure5 C/5-GFAP-3.tif]

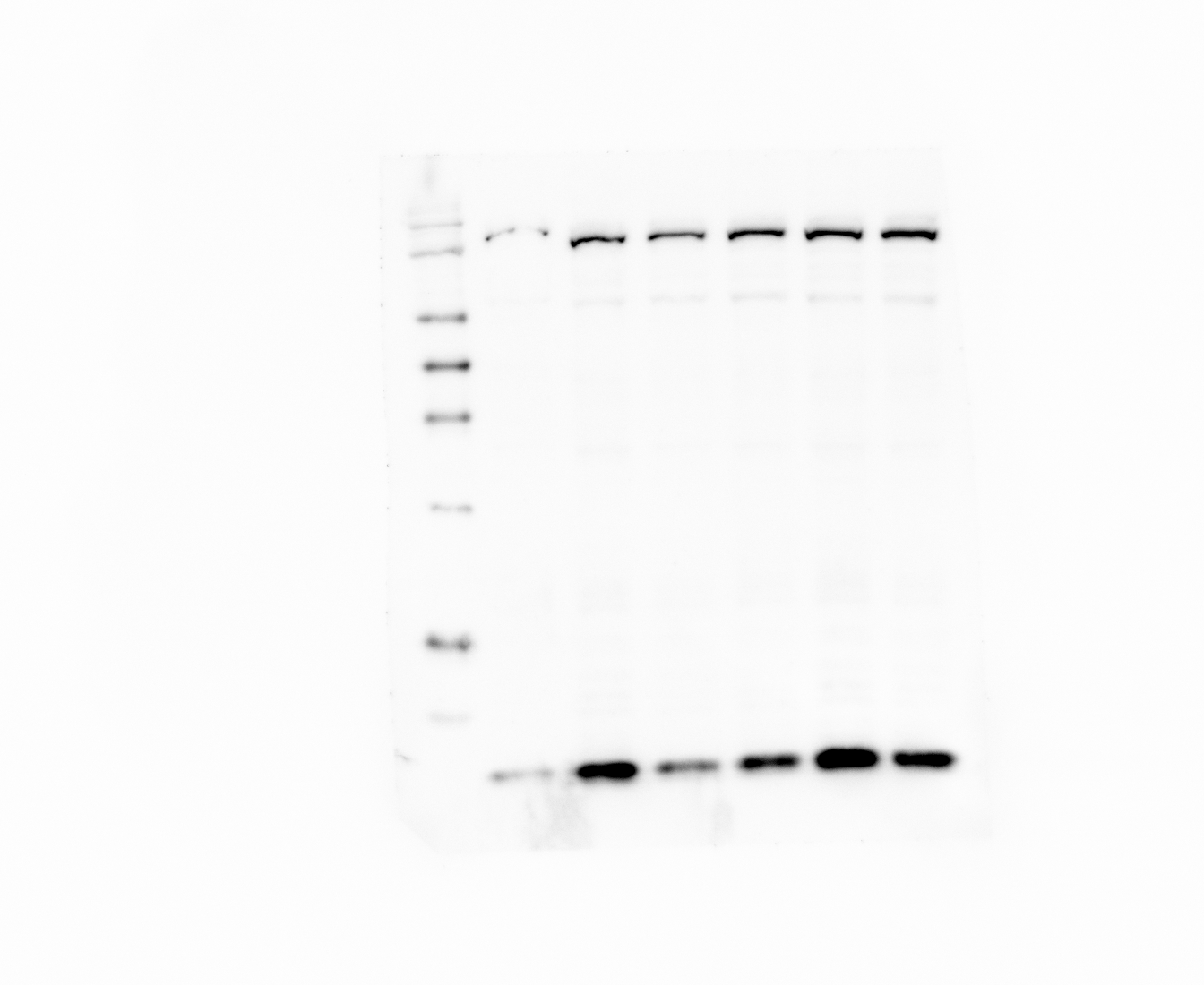

Supplement: Supplementary file 1 [file biomolecules-15-01188-s001.zip › File S1. Original Images for Blots/Figure5 C/5-IBA1-1 报告.tif]

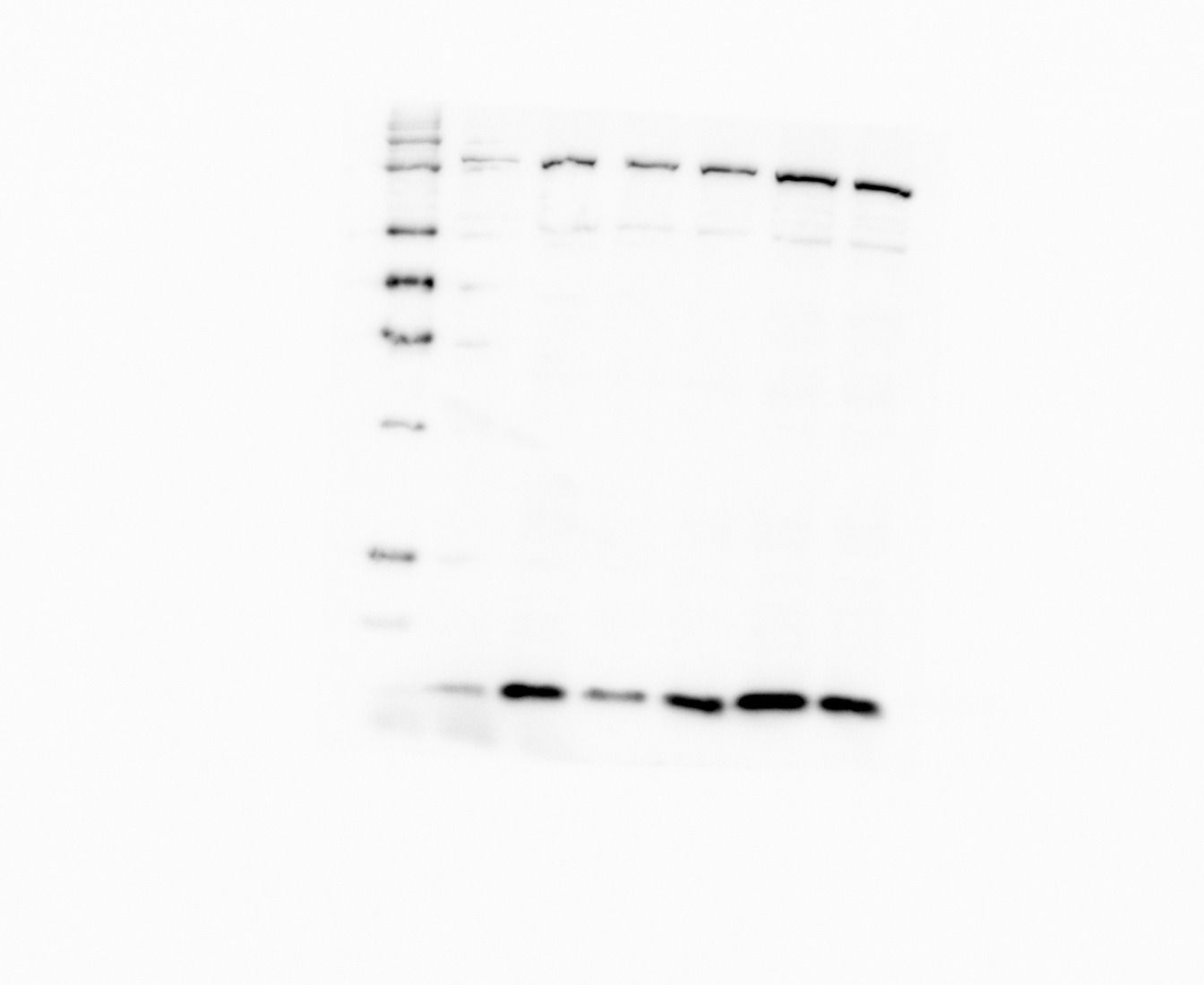

Supplement: Supplementary file 1 [file biomolecules-15-01188-s001.zip › File S1. Original Images for Blots/Figure5 C/5-IBA1-2.tif]

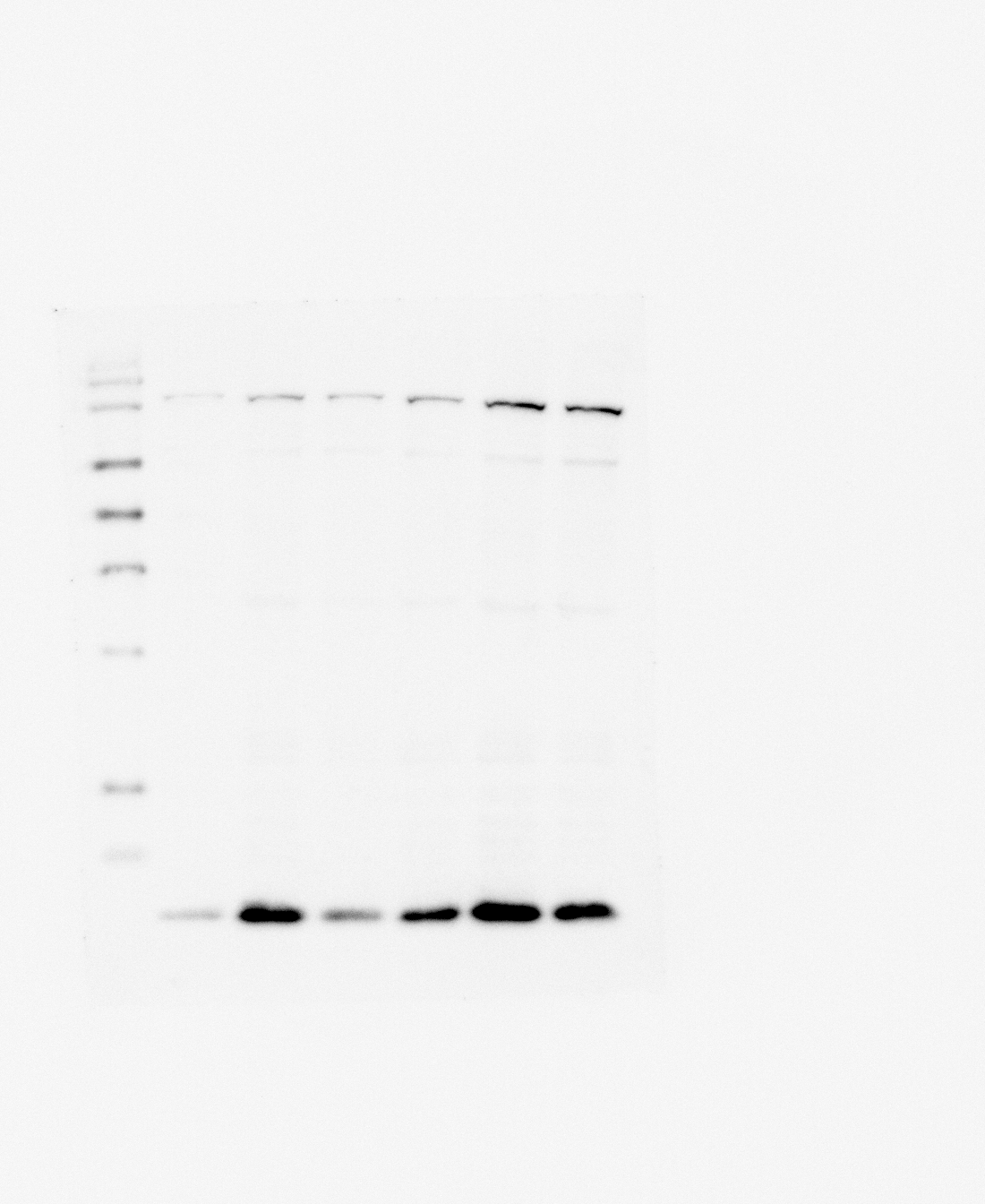

Supplement: Supplementary file 1 [file biomolecules-15-01188-s001.zip › File S1. Original Images for Blots/Figure5 C/5-IBA1-3.tif]

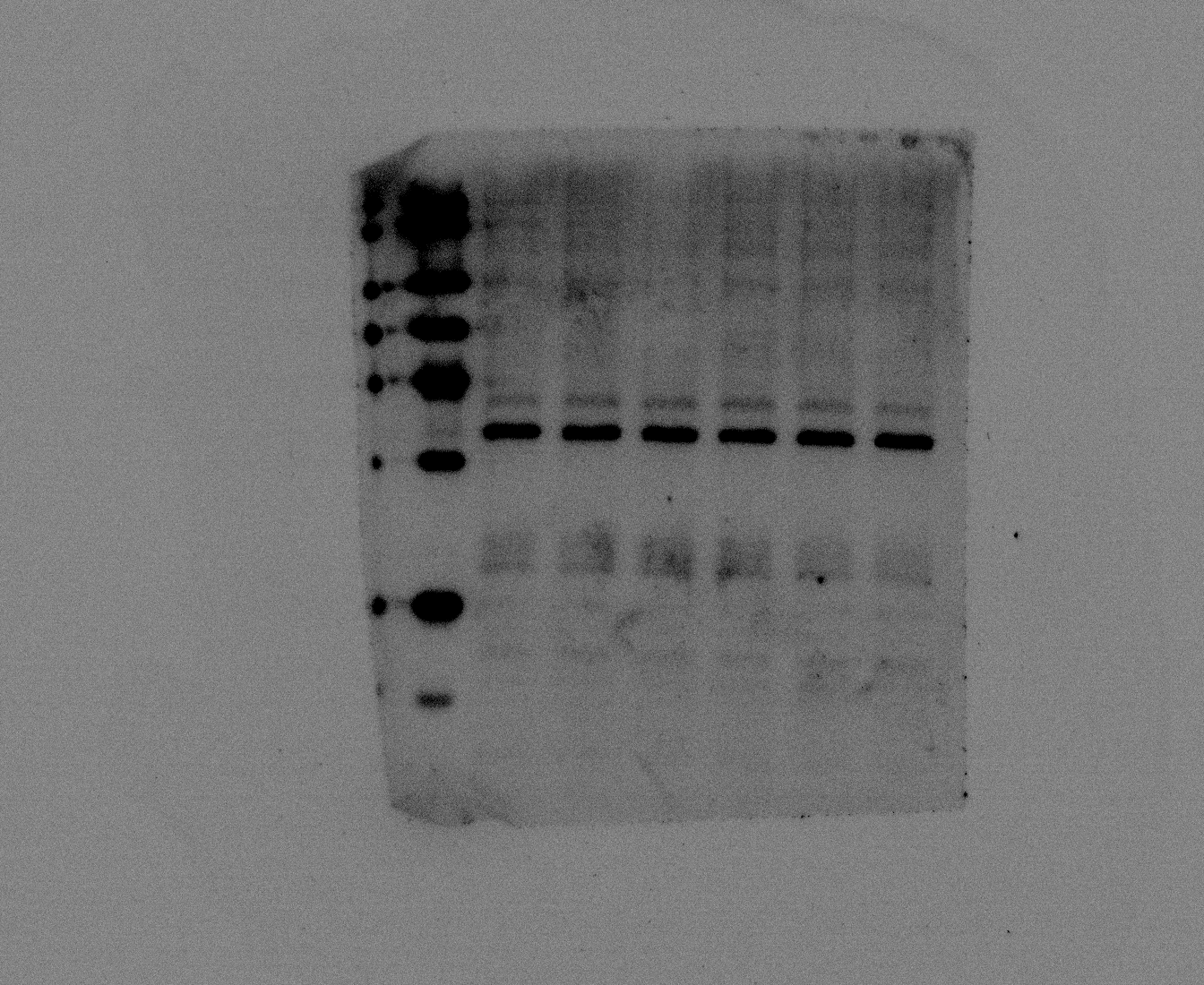

Supplement: Supplementary file 1 [file biomolecules-15-01188-s001.zip › File S1. Original Images for Blots/Figure5 C/5-β-actin-1.tif]

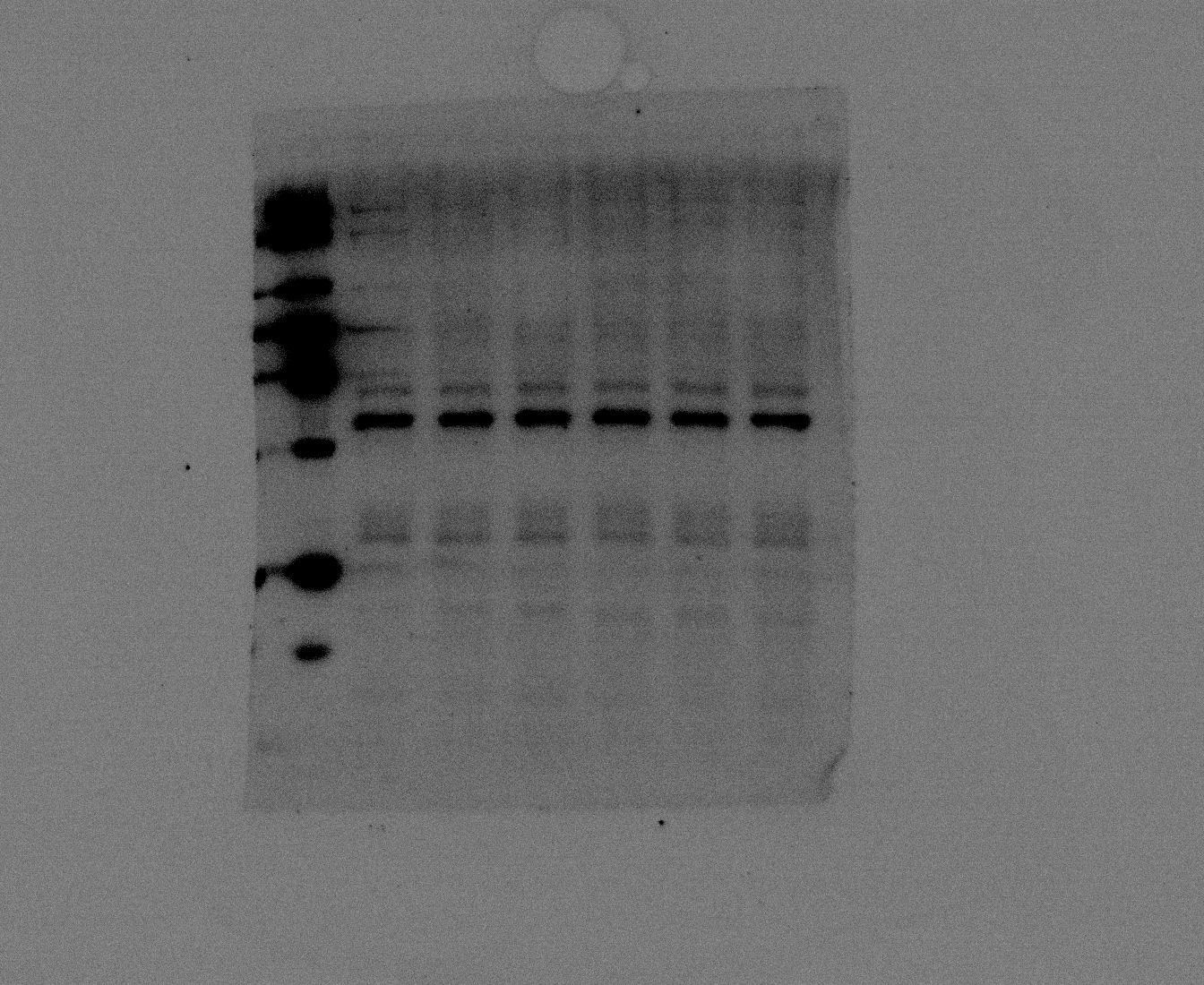

Supplement: Supplementary file 1 [file biomolecules-15-01188-s001.zip › File S1. Original Images for Blots/Figure5 C/5-β-actin-2 报告.tif]

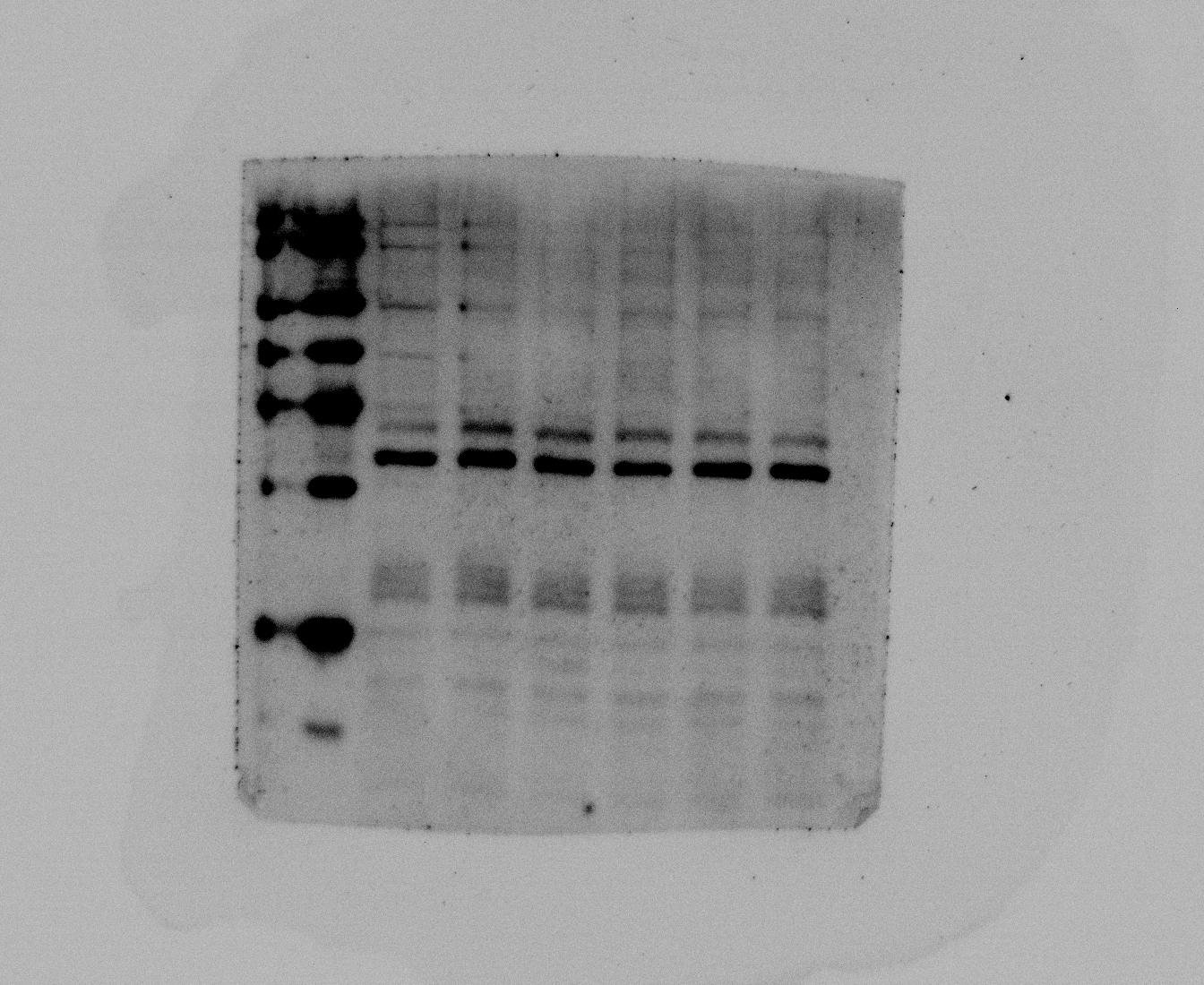

Supplement: Supplementary file 1 [file biomolecules-15-01188-s001.zip › File S1. Original Images for Blots/Figure5 C/5-β-actin-3.tif]

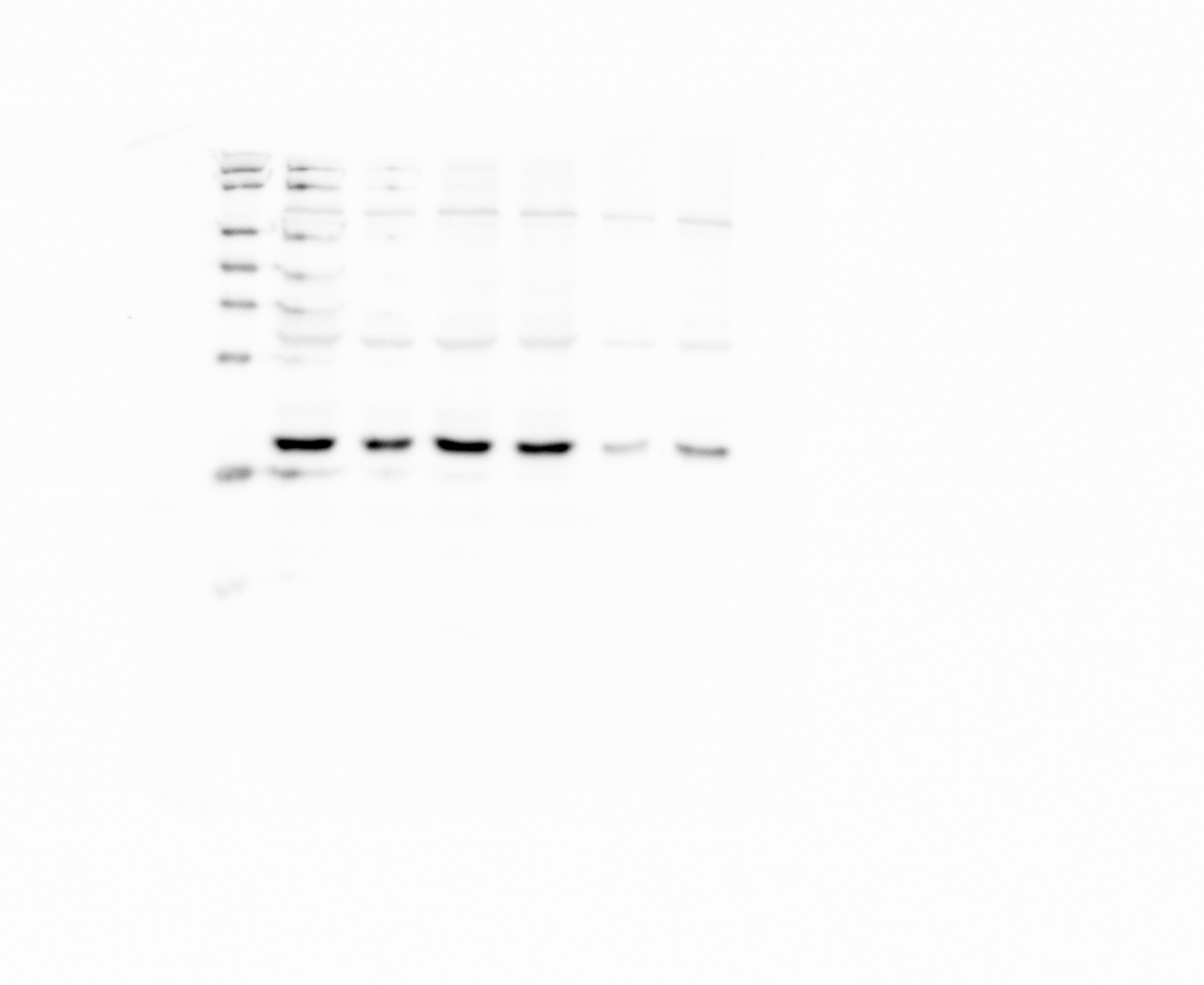

Supplement: Supplementary file 1 [file biomolecules-15-01188-s001.zip › File S1. Original Images for Blots/Figure6 C/6-BDNF-1.tif]

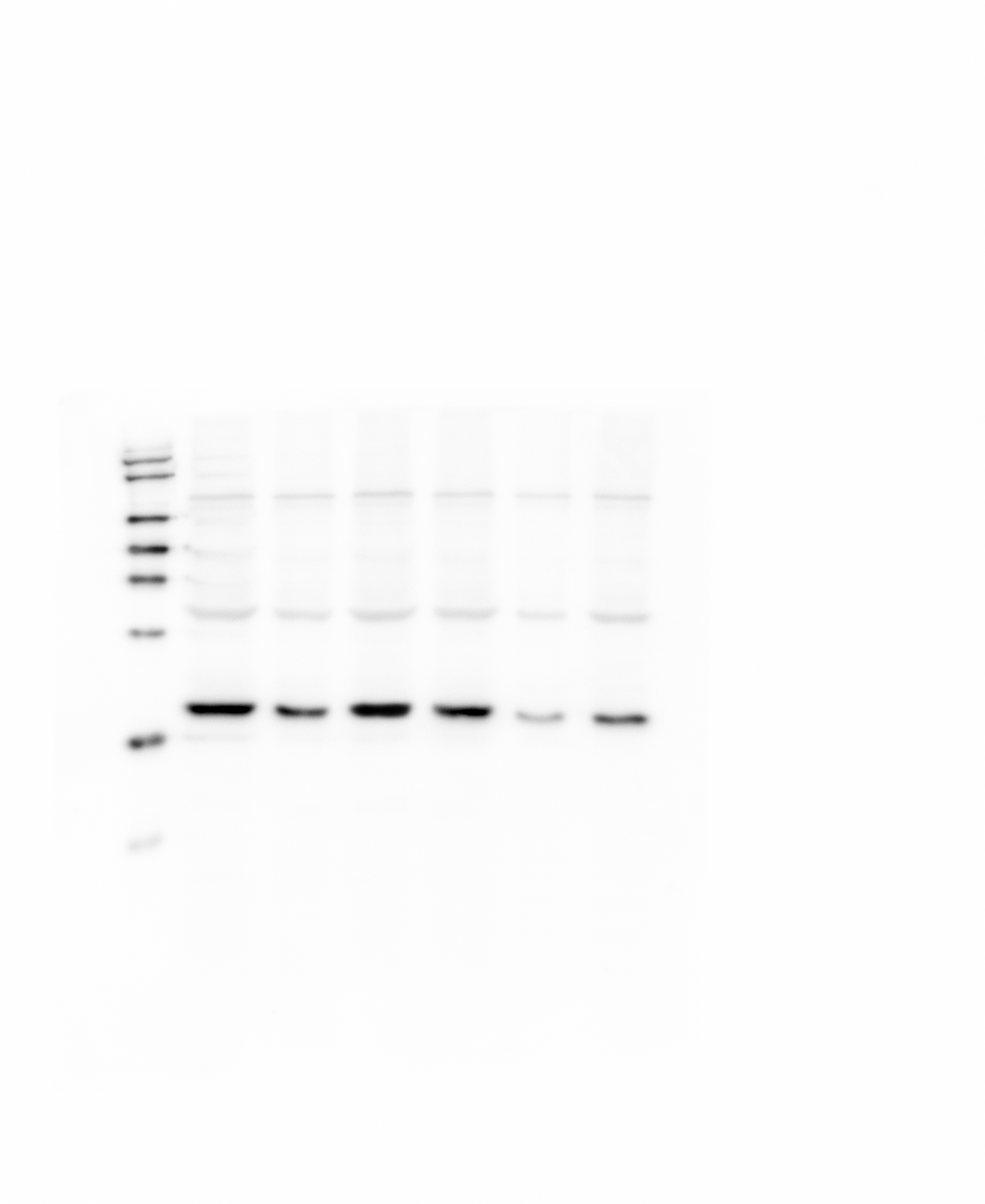

Supplement: Supplementary file 1 [file biomolecules-15-01188-s001.zip › File S1. Original Images for Blots/Figure6 C/6-BDNF-2 报告.tif]

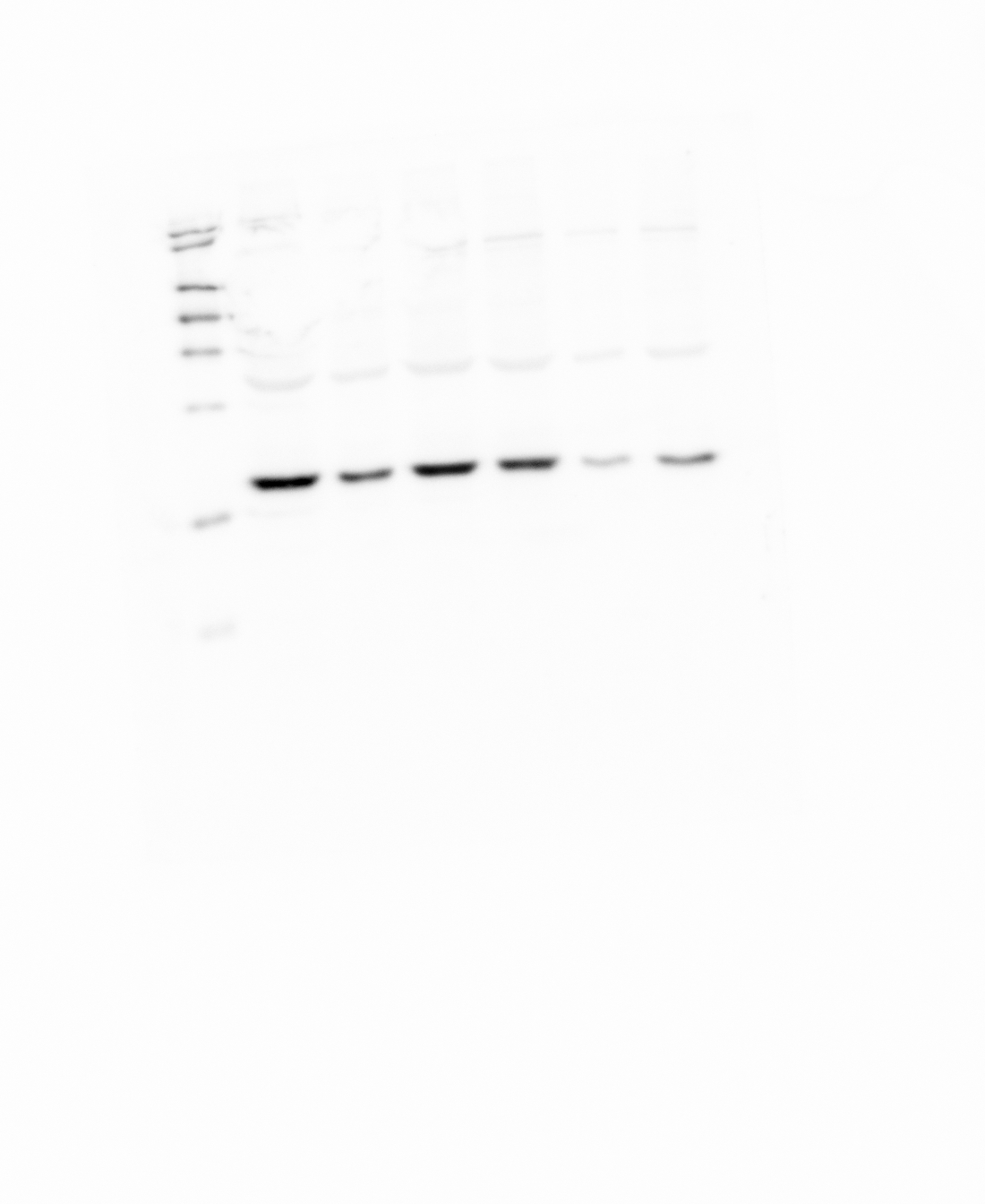

Supplement: Supplementary file 1 [file biomolecules-15-01188-s001.zip › File S1. Original Images for Blots/Figure6 C/6-BDNF-3.tif]

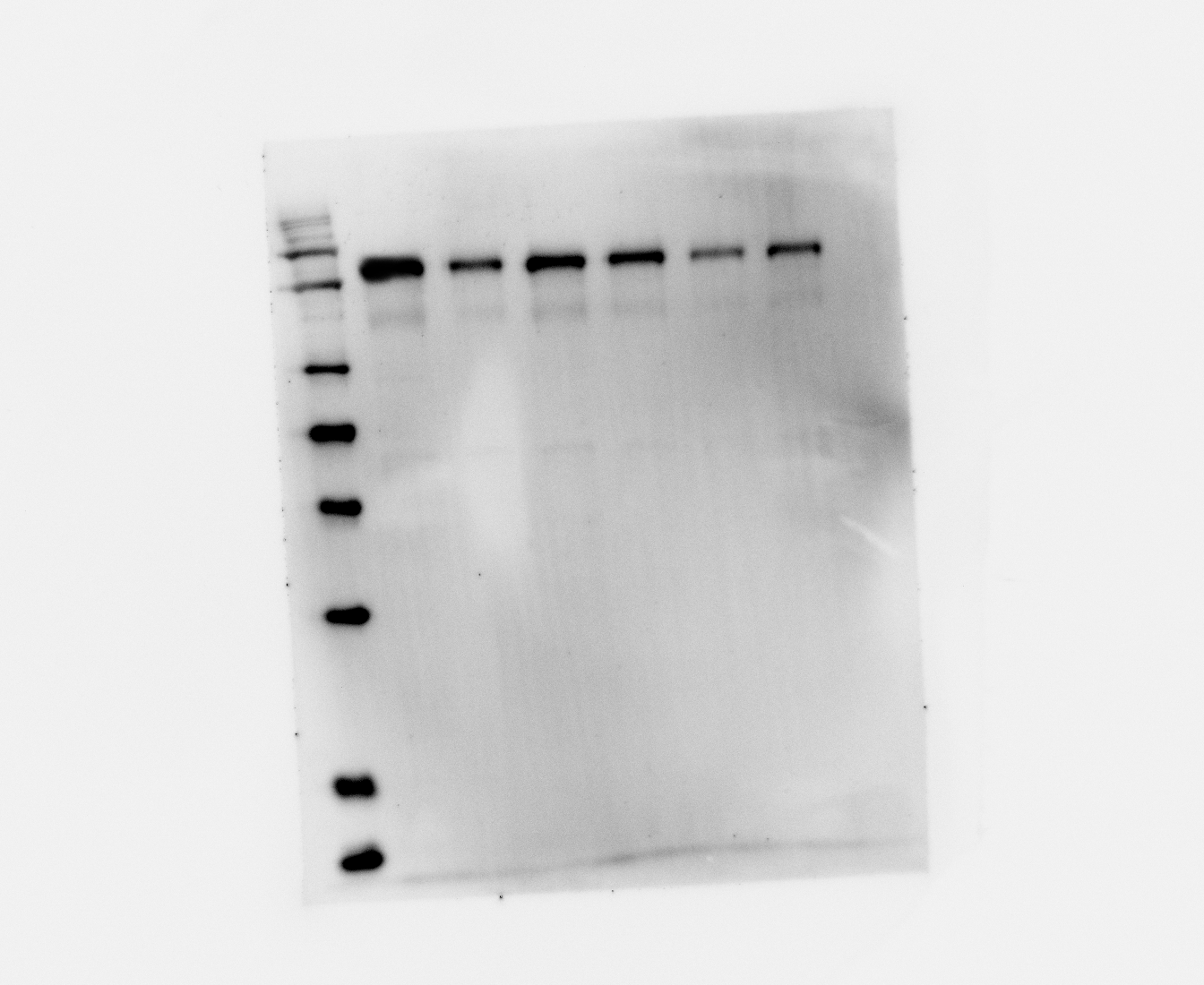

Supplement: Supplementary file 1 [file biomolecules-15-01188-s001.zip › File S1. Original Images for Blots/Figure6 C/6-p-TrkB-1.tif]

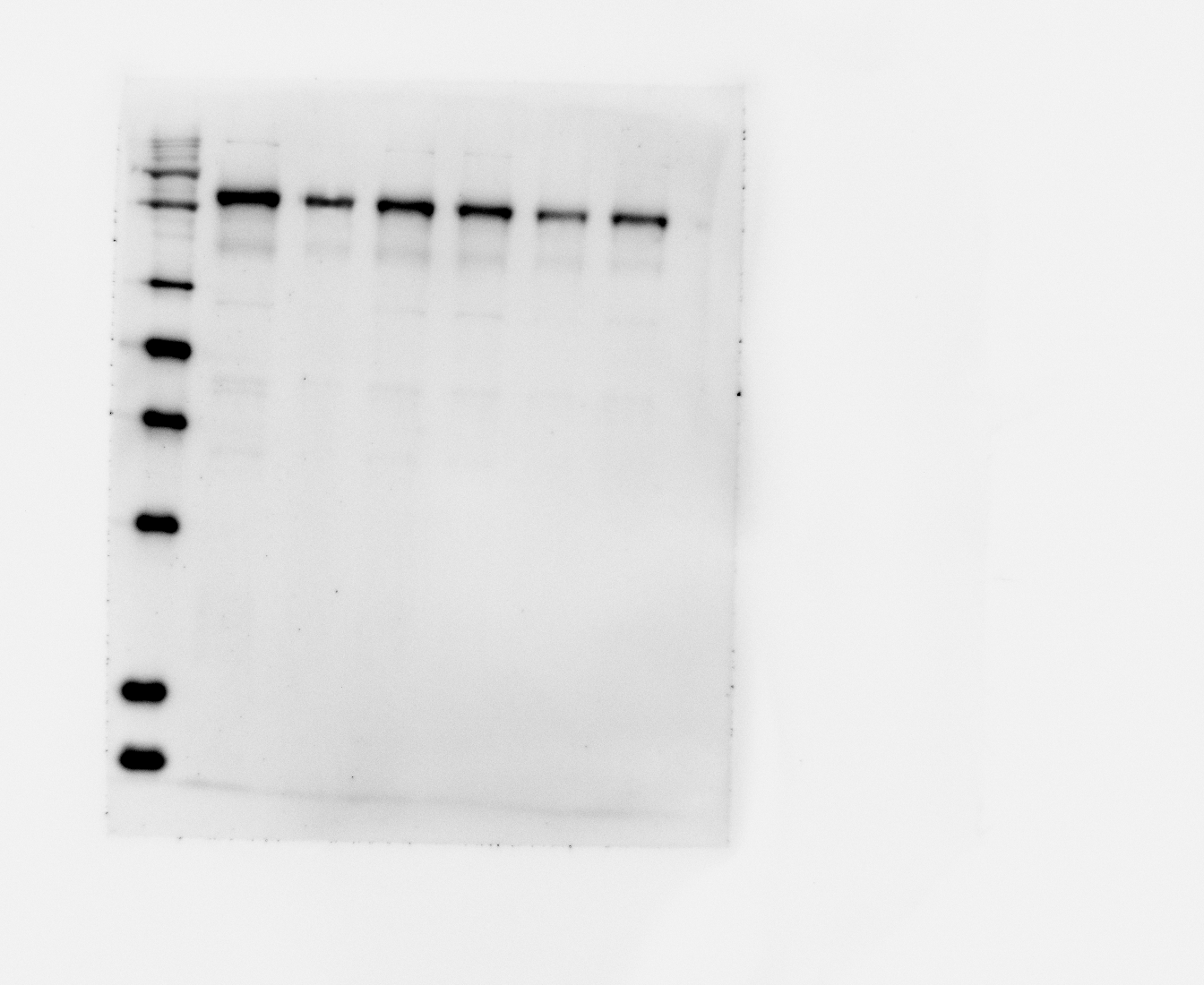

Supplement: Supplementary file 1 [file biomolecules-15-01188-s001.zip › File S1. Original Images for Blots/Figure6 C/6-p-TrkB-2 报告.tif]

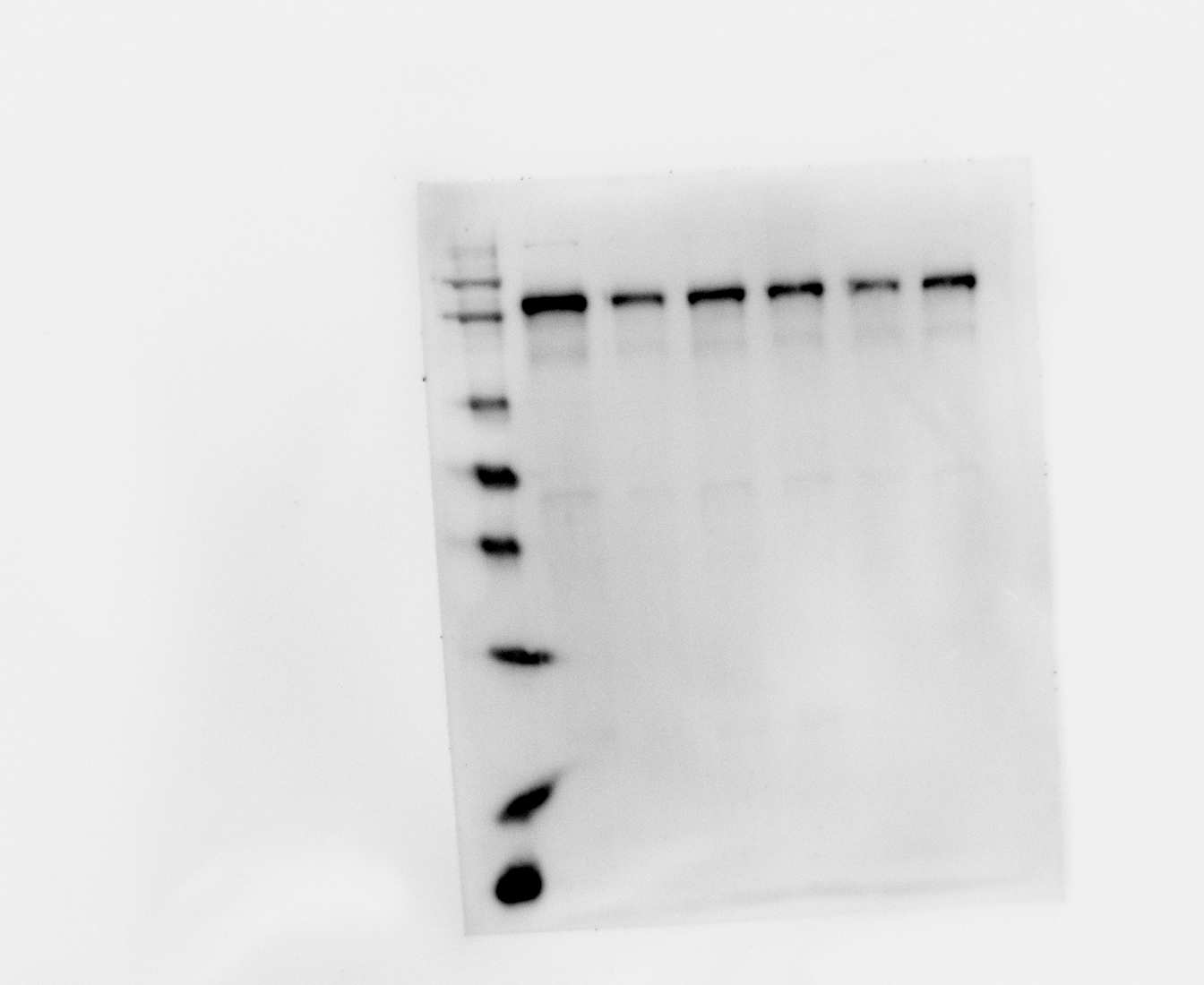

Supplement: Supplementary file 1 [file biomolecules-15-01188-s001.zip › File S1. Original Images for Blots/Figure6 C/6-p-TrkB-3.tif]

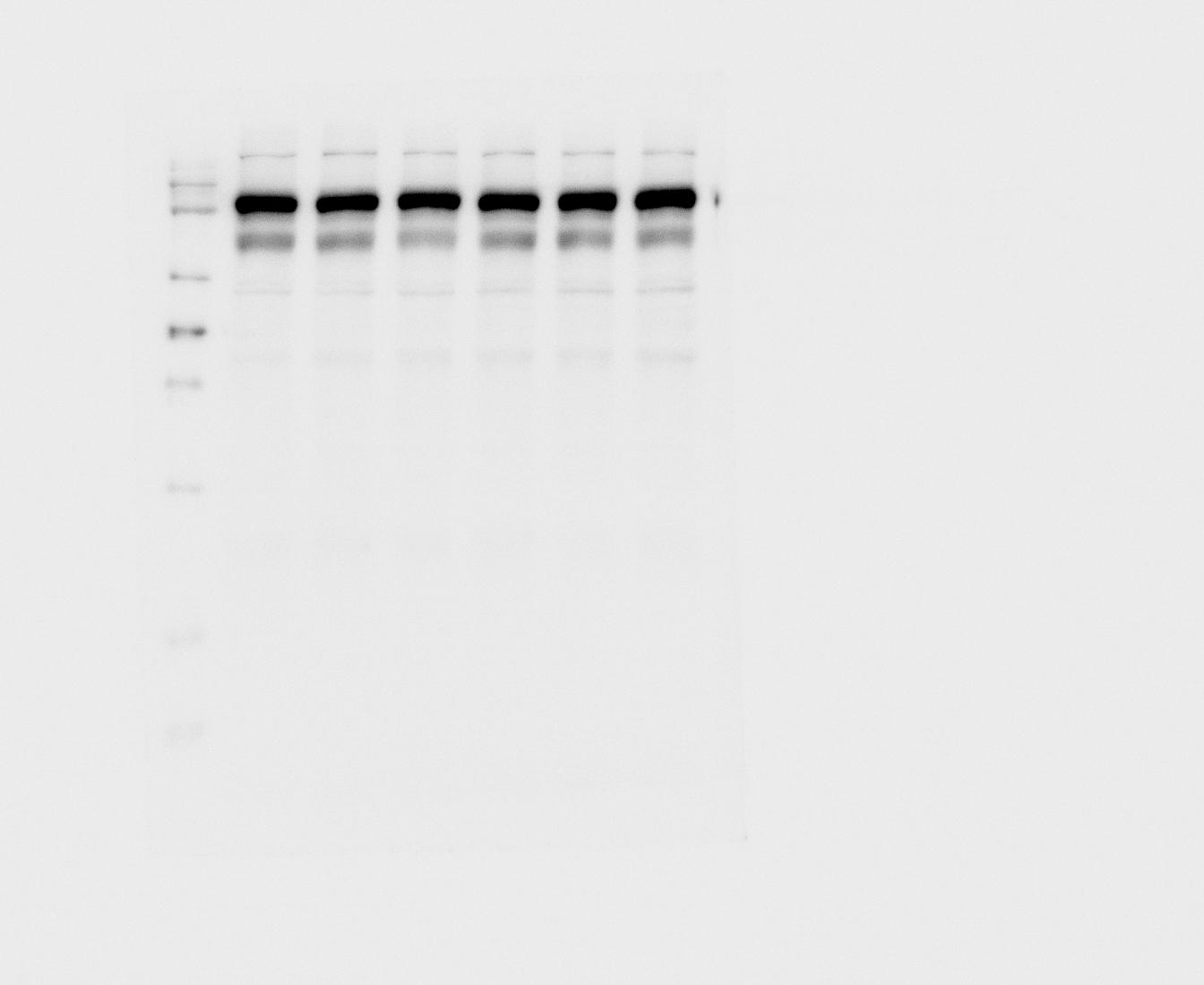

Supplement: Supplementary file 1 [file biomolecules-15-01188-s001.zip › File S1. Original Images for Blots/Figure6 C/6-TrkB-1.tif]

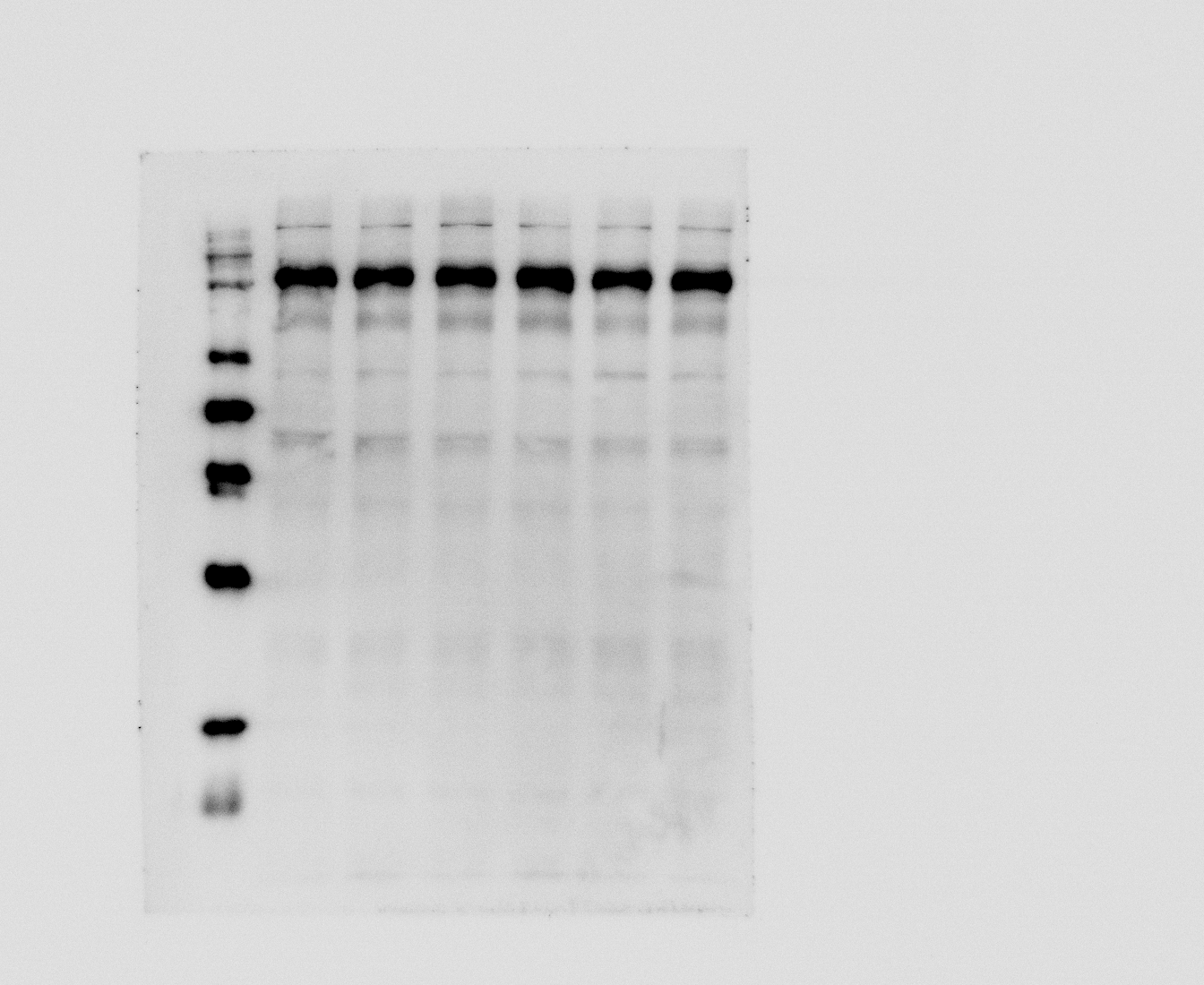

Supplement: Supplementary file 1 [file biomolecules-15-01188-s001.zip › File S1. Original Images for Blots/Figure6 C/6-TrkB-2 报告.tif]

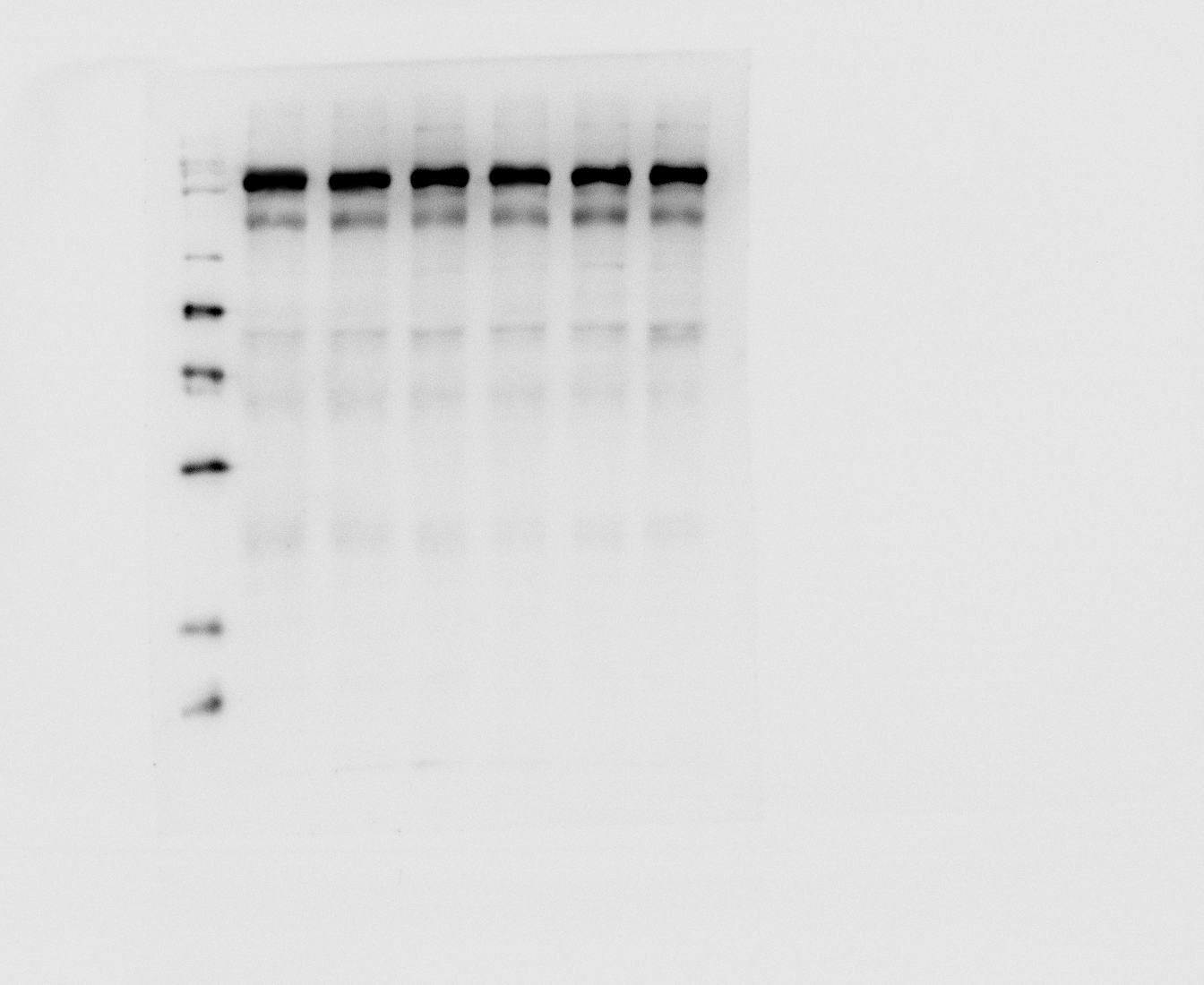

Supplement: Supplementary file 1 [file biomolecules-15-01188-s001.zip › File S1. Original Images for Blots/Figure6 C/6-TrkB-3.tif]

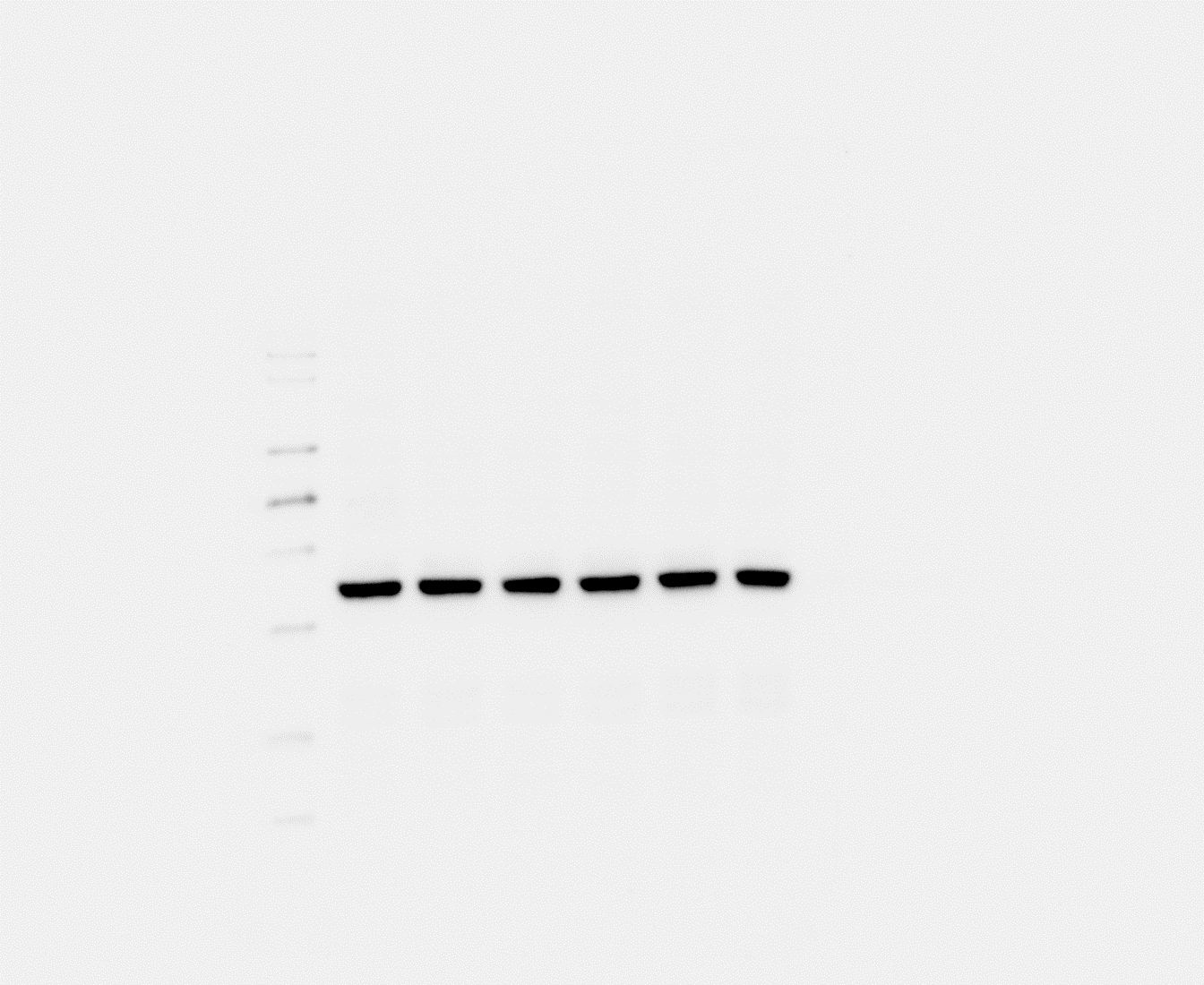

Supplement: Supplementary file 1 [file biomolecules-15-01188-s001.zip › File S1. Original Images for Blots/Figure6 C/6-β-actin-1 报告.tif]

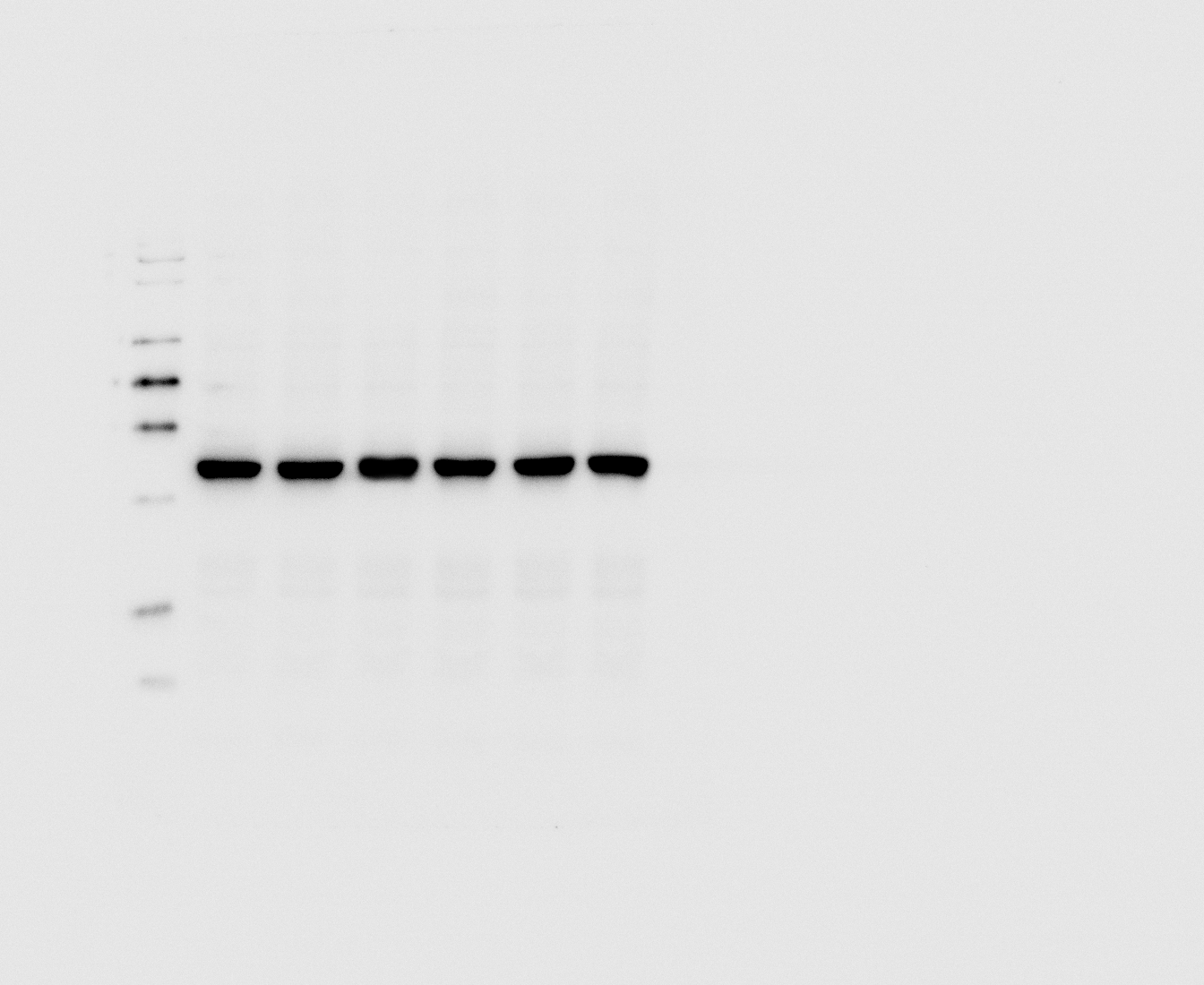

Supplement: Supplementary file 1 [file biomolecules-15-01188-s001.zip › File S1. Original Images for Blots/Figure6 C/6-β-actin-2.tif]

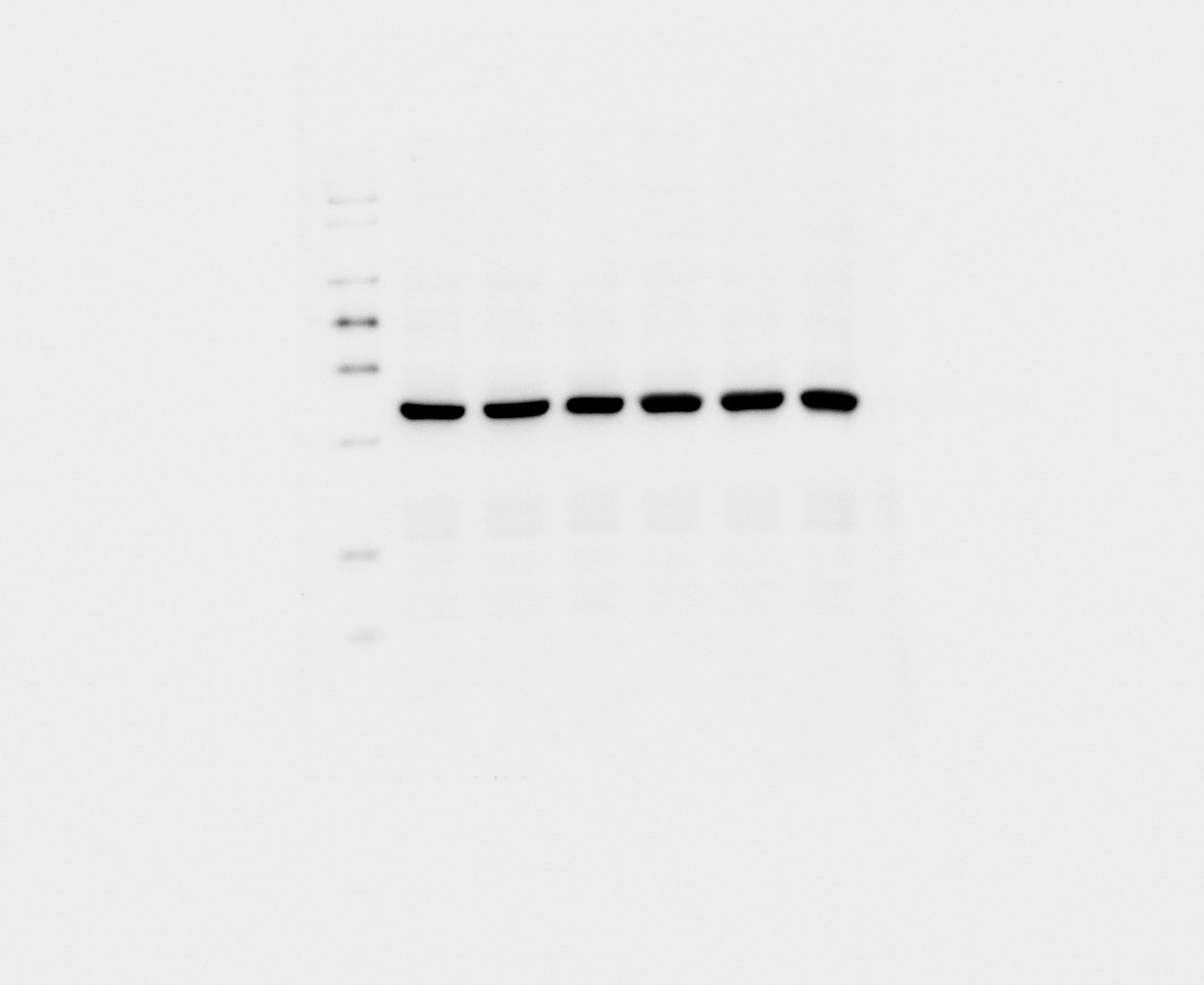

Supplement: Supplementary file 1 [file biomolecules-15-01188-s001.zip › File S1. Original Images for Blots/Figure6 C/6-β-actin-3.tif]

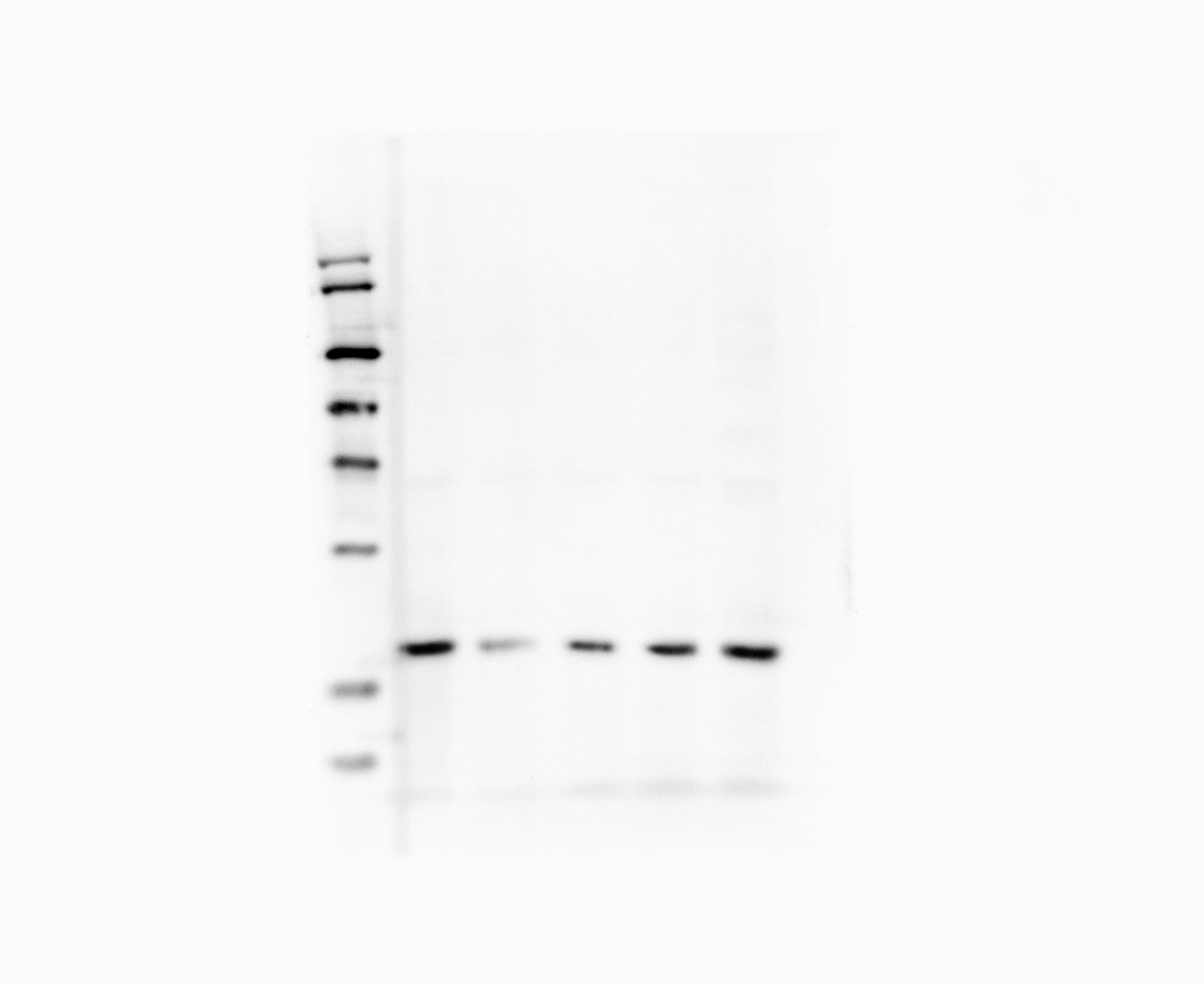

Supplement: Supplementary file 1 [file biomolecules-15-01188-s001.zip › File S1. Original Images for Blots/Figure7 C/BDNF-1.tif]

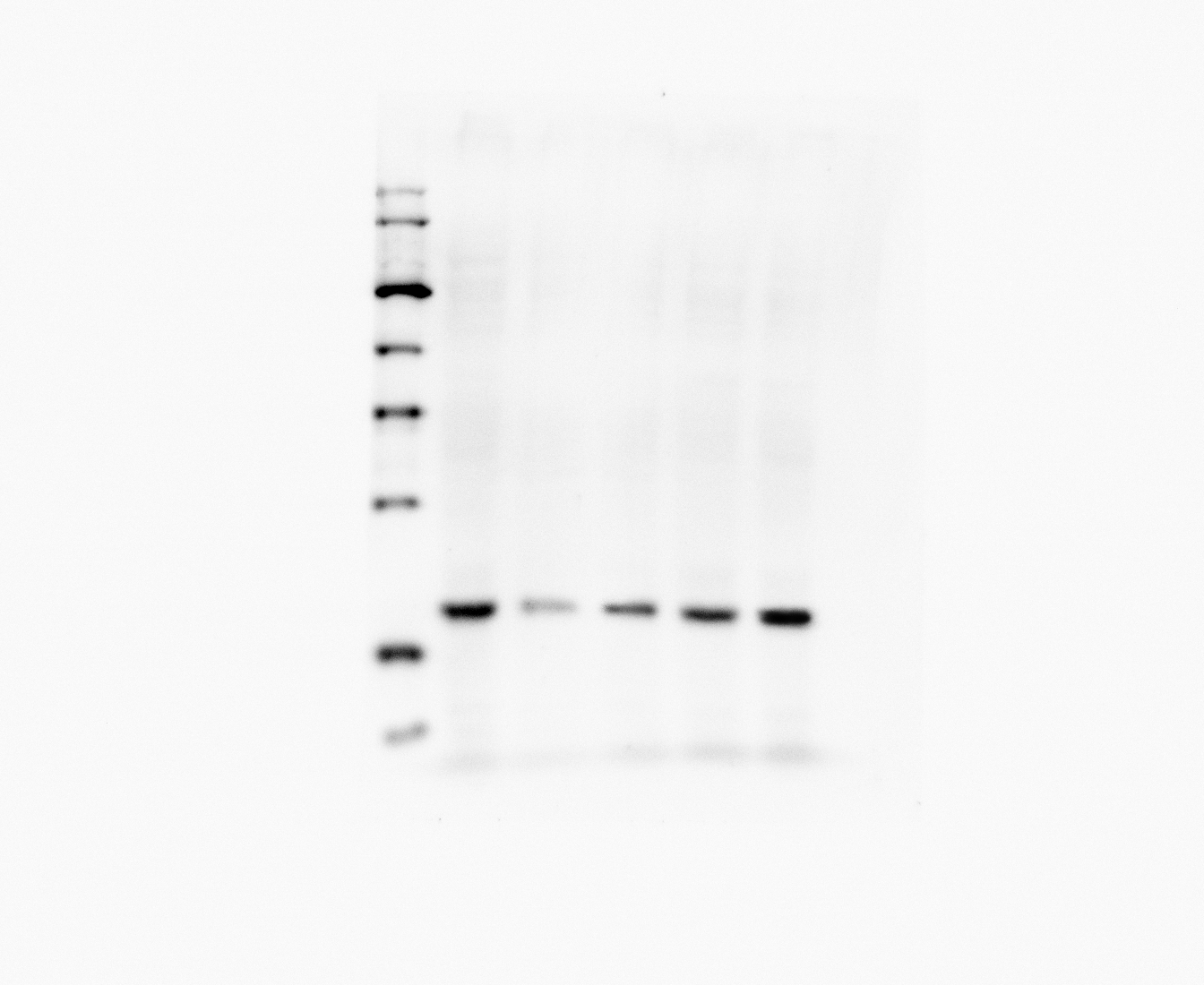

Supplement: Supplementary file 1 [file biomolecules-15-01188-s001.zip › File S1. Original Images for Blots/Figure7 C/BDNF-2.tif]

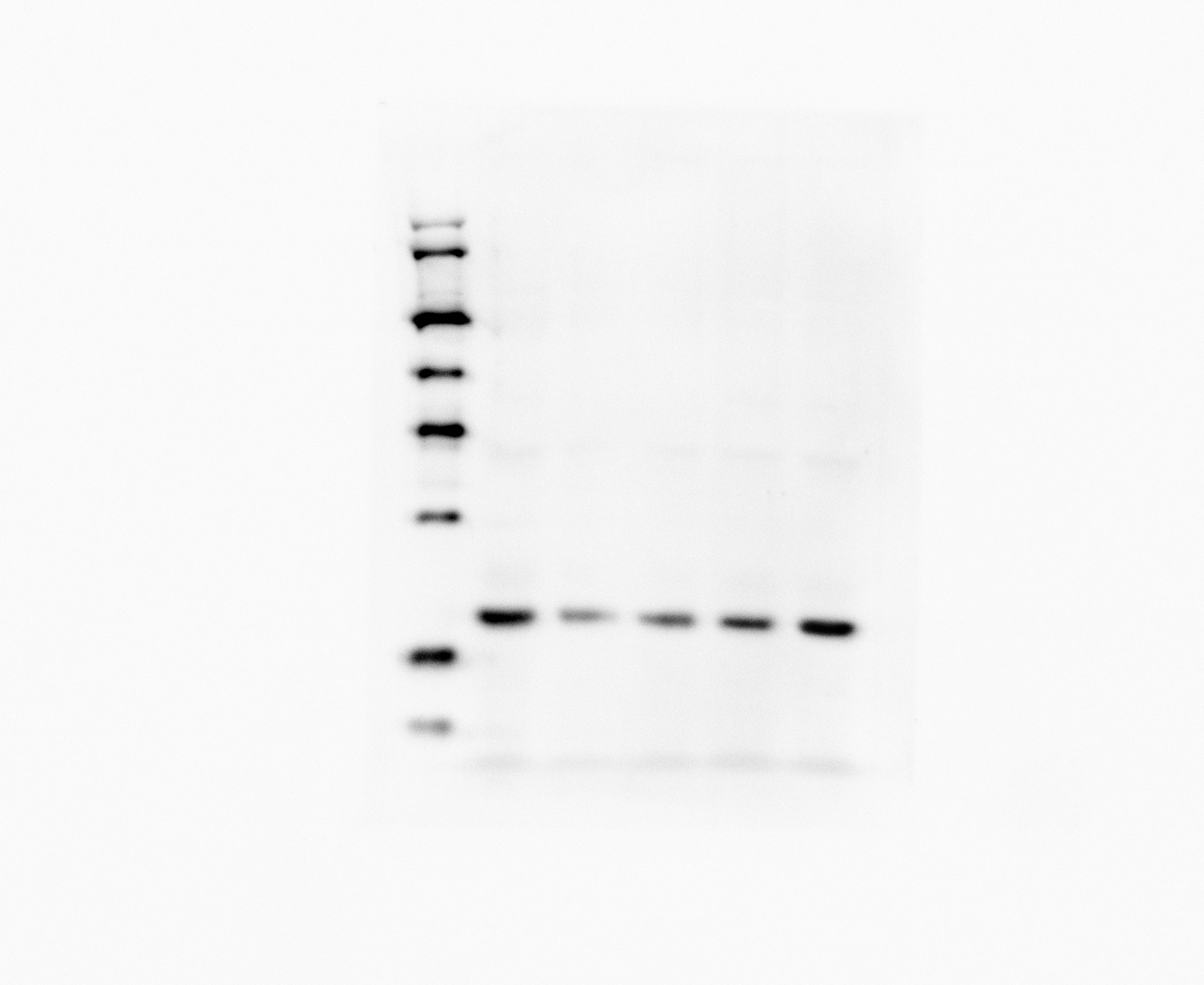

Supplement: Supplementary file 1 [file biomolecules-15-01188-s001.zip › File S1. Original Images for Blots/Figure7 C/BDNF-3.tif]

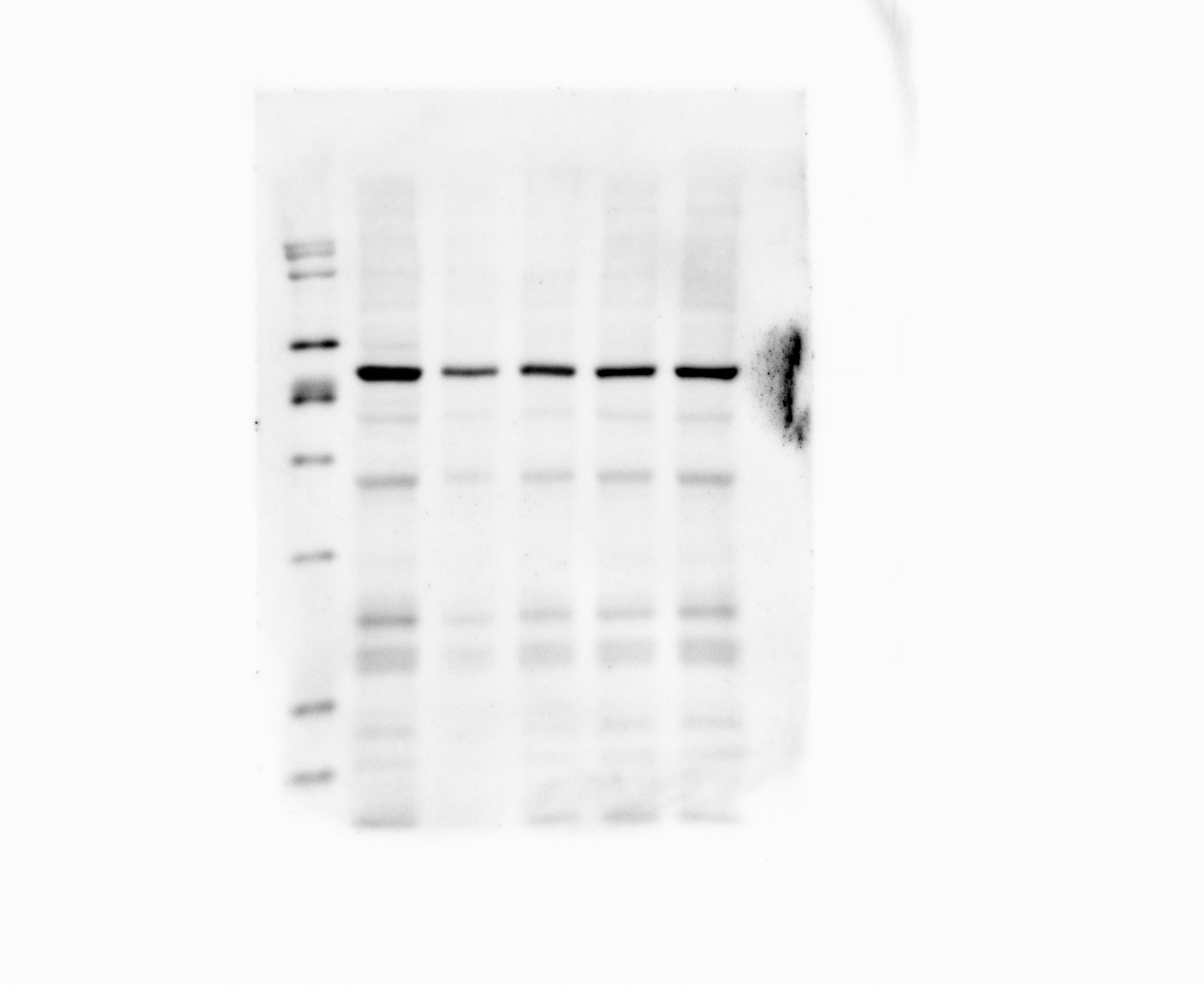

Supplement: Supplementary file 1 [file biomolecules-15-01188-s001.zip › File S1. Original Images for Blots/Figure7 C/METTL3-1.tif]

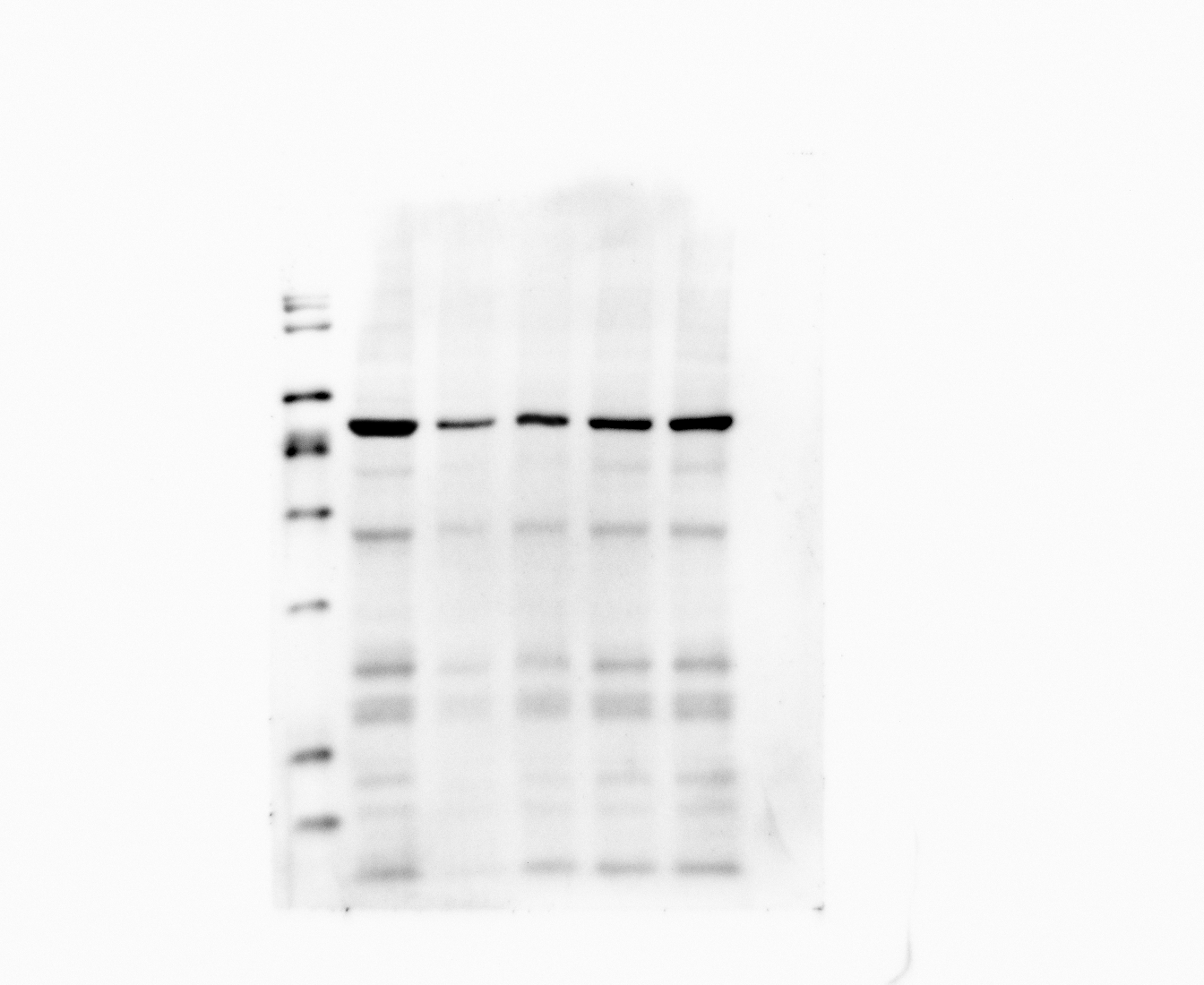

Supplement: Supplementary file 1 [file biomolecules-15-01188-s001.zip › File S1. Original Images for Blots/Figure7 C/METTL3-2.tif]

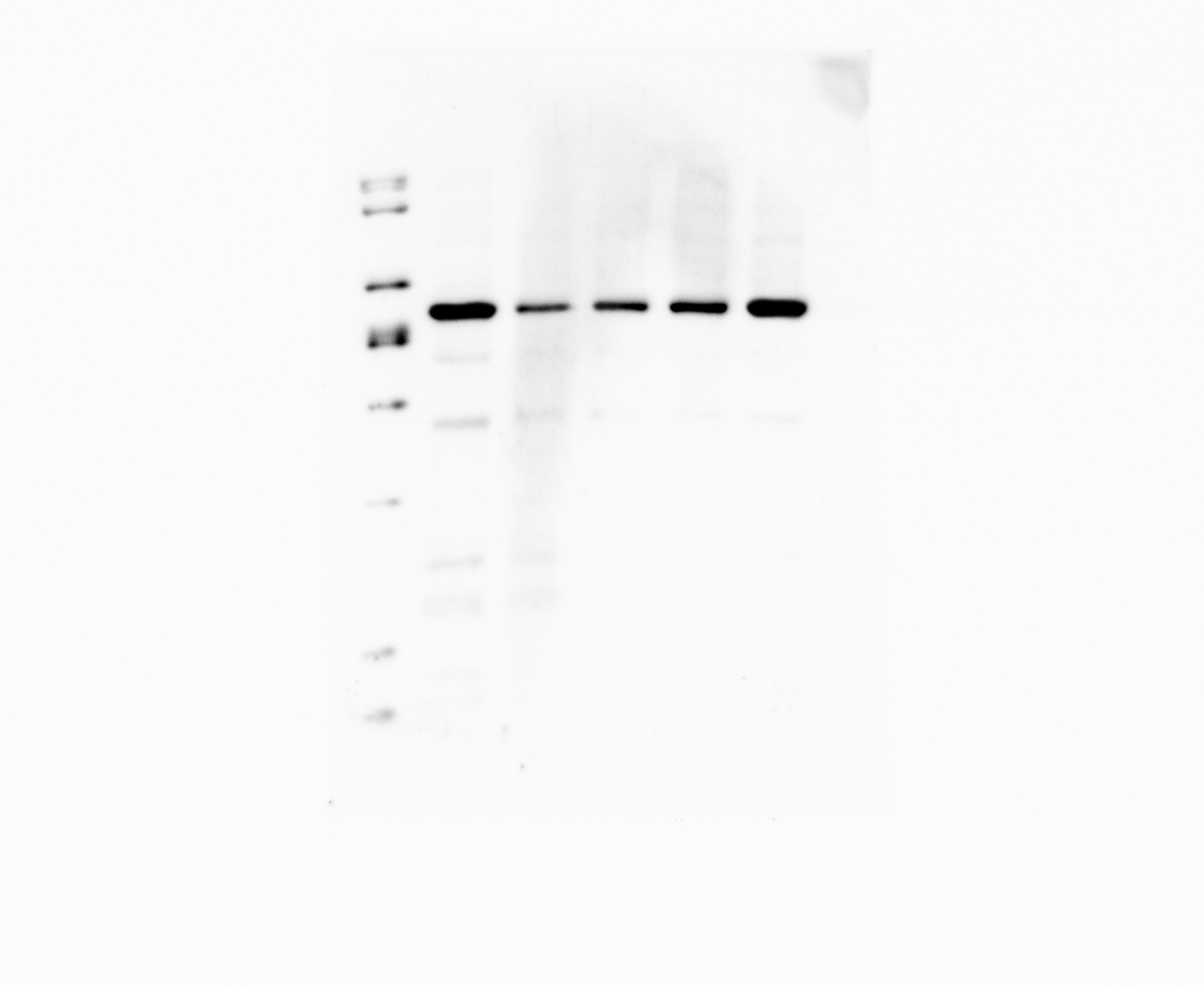

Supplement: Supplementary file 1 [file biomolecules-15-01188-s001.zip › File S1. Original Images for Blots/Figure7 C/METTL3-3.tif]

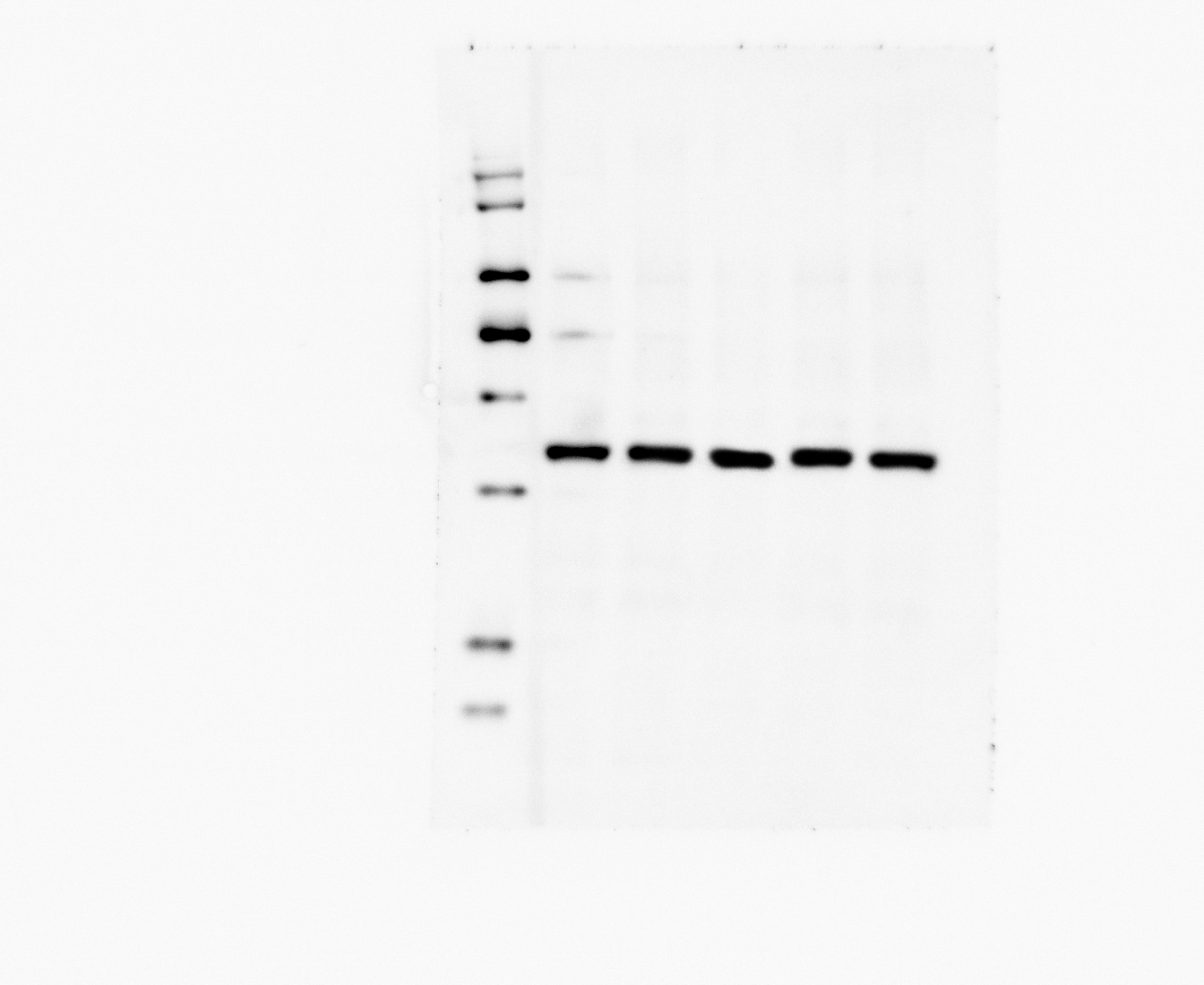

Supplement: Supplementary file 1 [file biomolecules-15-01188-s001.zip › File S1. Original Images for Blots/Figure7 C/β-actin-1.tif]

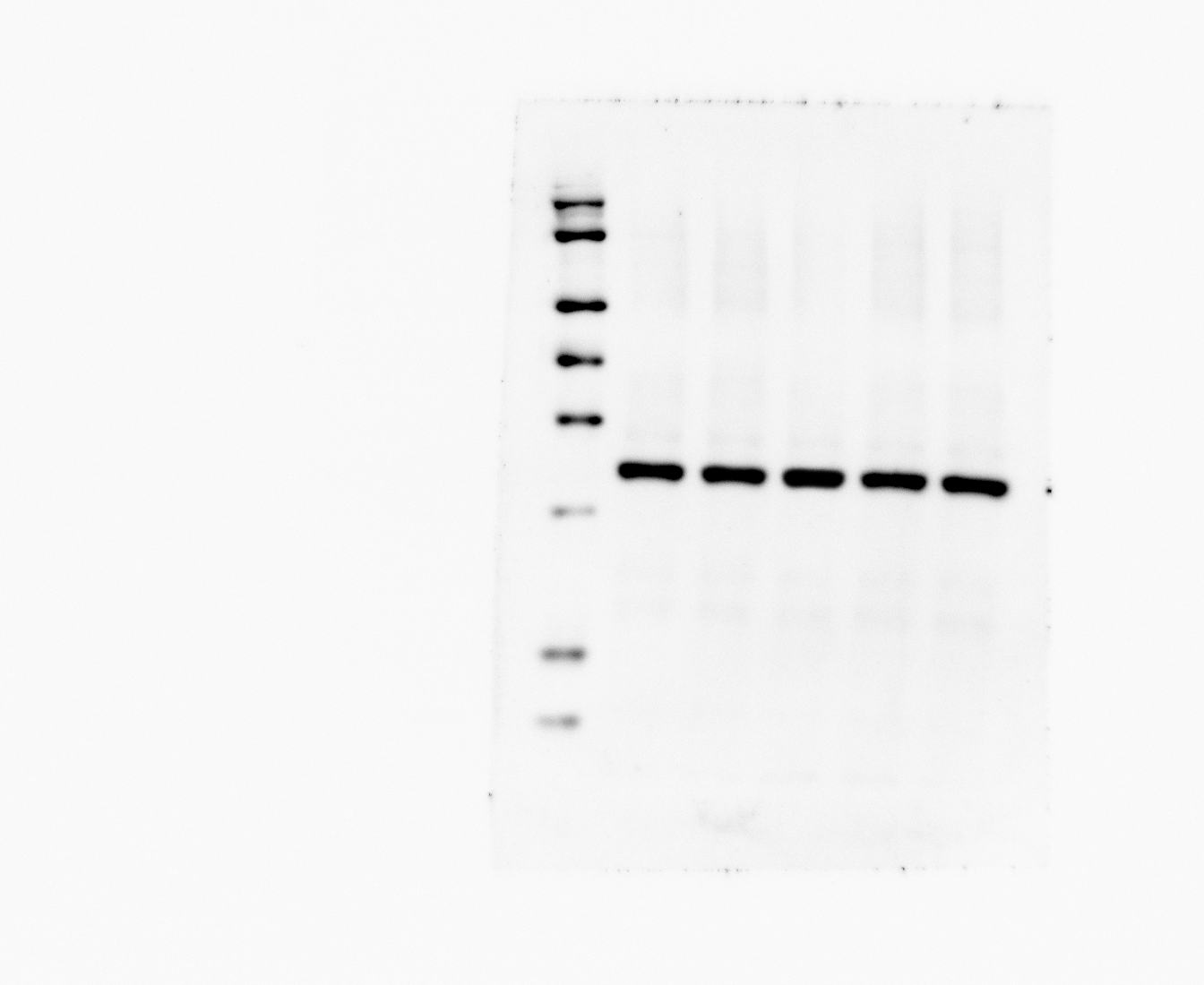

Supplement: Supplementary file 1 [file biomolecules-15-01188-s001.zip › File S1. Original Images for Blots/Figure7 C/β-actin-2.tif]

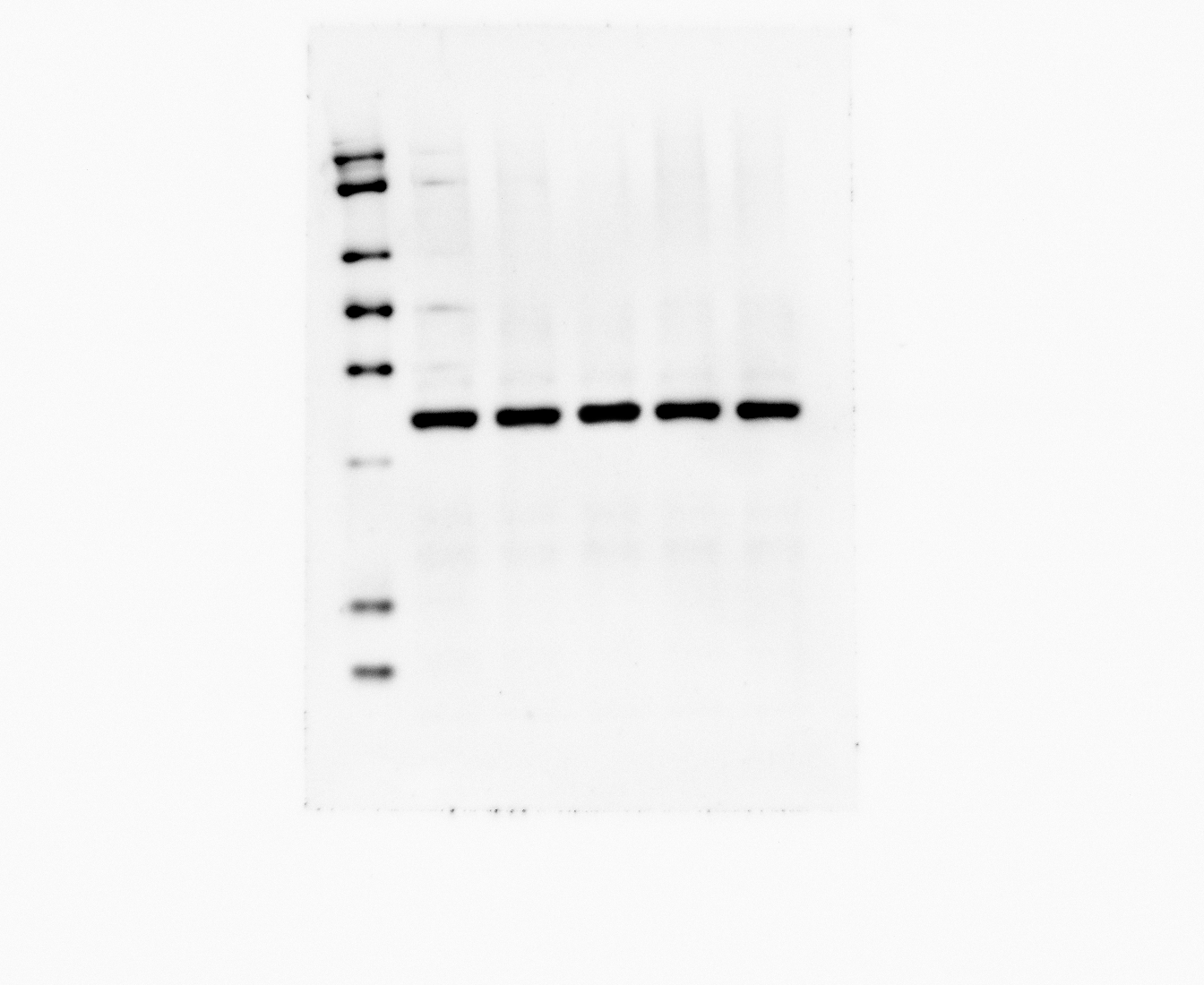

Supplement: Supplementary file 1 [file biomolecules-15-01188-s001.zip › File S1. Original Images for Blots/Figure7 C/β-actin-3.tif]
